# Novel Thiazole-Fused [4,5-*g*] or [5,4-*g*]Quinazolin-8-ones and Their Quinazoline Analogues: Synthesis and Biological Evaluation

**DOI:** 10.3390/ph17111452

**Published:** 2024-10-30

**Authors:** Nathan Broudic, Alexandra Pacheco-Benichou, Cécile Corbière, Blandine Baratte, Thomas Robert, Stéphane Bach, Hélène Solhi, Rémy Le Guével, Corinne Fruit, Thierry Besson

**Affiliations:** 1 Univ Rouen Normandie, INSA Rouen Normandie, CNRS, COBRA UMR 6014, F-76000 Rouen, France; nathan.broudic@univ-rouen.fr (N.B.); benichoualexandra@yahoo.fr (A.P.-B.); corinne.fruit@univ-rouen.fr (C.F.); 2 Univ Rouen Normandie, ABTE UR4651, F-76000 Rouen, France; cecile.corbiere@univ-rouen.fr; 3 Sorbonne Université, CNRS, UMR8227, Integrative Biology of Marine Models Laboratory (LBI2M), Station Biologique de Roscoff, F-29680 Roscoff, France; baratte@sb-roscoff.fr (B.B.); trobert@sb-roscoff.fr (T.R.); stephane.bach@sb-roscoff.fr (S.B.); 4 Sorbonne Université, CNRS, FR2424, Plateforme de Criblage KISSf (Kinase Inhibitor Specialized Screening Facility), Station Biologique de Roscoff, F-29680 Roscoff, France; 5 Univ Rennes, Plateform ImPACcell, BIOSIT, F-35000 Rennes, France; helene.solhi@univ-rennes.fr (H.S.); remy.leguevel@univ-rennes.fr (R.L.G.)

**Keywords:** thiazoloquinazolinones, thiazoloquinazolines, 4,5-dichloro-1,2,3-dithiazolium chloride, microwave-assisted chemistry, cytotoxicity, kinase inhibition, DYRK1A kinase

## Abstract

**Background/Objectives:** In connection with previous work on V-shaped polycyclic thiazolo[5,4-*f*]quinazolin-9-one and [5,4-*f*]quinazoline derivatives that can modulate the activity of various kinases, the synthesis of straight thiazole-fused [4,5-*g*] or [5,4-*g*]quinazolin-8-ones and quinazoline derivatives hitherto undescribed was envisioned. **Methods:** An innovative protocol allowed to obtain the target structures. The synthesis of inverted thiazolo[4,5-*h*] and [5,4-*h*]quinazolin-8-one derivatives was also explored with the aim of comparing biological results. The compounds obtained were tested against a representative panel of eight mammalian protein kinases of human origin: CDK9/CyclinT, Haspin, Pim-1, GSK-3β, CK-1ε, JAK3, CLK1 and DYRK1A. **Results and Conclusions:** The results obtained show that the novel linear thiazoloquinazolines are not kinase inhibitors. The cytotoxicity of these newly synthesized compounds was assessed against seven representative tumor cell lines (human cancers: Huh7-D12, Caco-2, HCT-116, MCF-7, MDA-MB-231, MDA-MB-468 and PC-3). The majority of these novel molecules proved capable of inhibiting the growth of the tested cells. The 7-Benzyl-8-oxo-7,8-dihydrothiazolo[5,4-*g*]quinazolinones **5b** and **6b** are the most potent, with IC_50_ values in the micromolar range. None of these compounds showed toxicity against normal cells. A larger program of investigations will be launched to investigate the real potential interest of such compounds in anticancer applications.

## 1. Introduction

**Figure 1:**
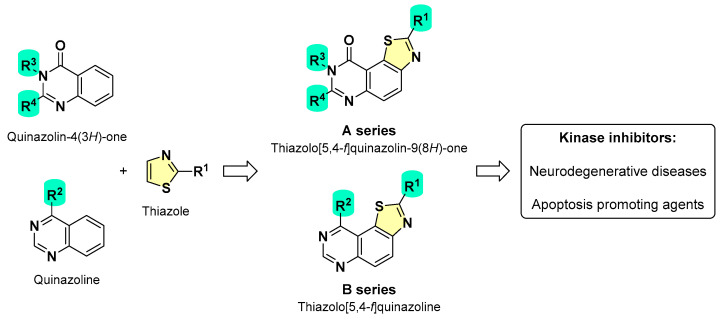
Thiazolo[5,4-*f*]quinazoline (A series) and thiazolo[5,4-*f*]quinazolin-9(8*H*)-one (B series) derivatives previously developed in our group.

For over 20 years, our group has worked on chemical methods allowing access to *N*,*S*-containing heteroarenes with the aim of creating molecules of therapeutic interest. In this context, we have developed thiazolo[5,4-*f*]quinazolin-9(8*H*)-ones (A series) [1,2,3,4] and thiazolo[5,4-*f*]quinazoline (B series) derivatives [5,6,7,8], a group of conjugated heterocyclic compounds that are effective inhibitors of kinases involved in neurodegenerative diseases (e.g., Alzheimer’s disease and Down’s syndrome) or in controlling the cell cycle (Figure 1). These studies suggested that the angular shape of these compounds allows the functional groups and parts of the molecule involved to be at a favorable distance from the amino acid residues of interest within the catalytic pocket [8,9].

These studies were also linked to chemical investigations on the use of 4,5-dichloro-1,2,3-dithiazolium chloride (commonly called Appel salt, AS), a versatile reagent capable of extending heterocyclic systems with a C2-functionalized thiazole unit [10,11]. Syntheses of these molecules are usually achieved in seven or eight steps and include some drawbacks such as a bromination step that is not completely regioselective, requiring tedious purification operations and often leading to low reaction yields [1,2,3,4,5,6].

**Figure 2:**
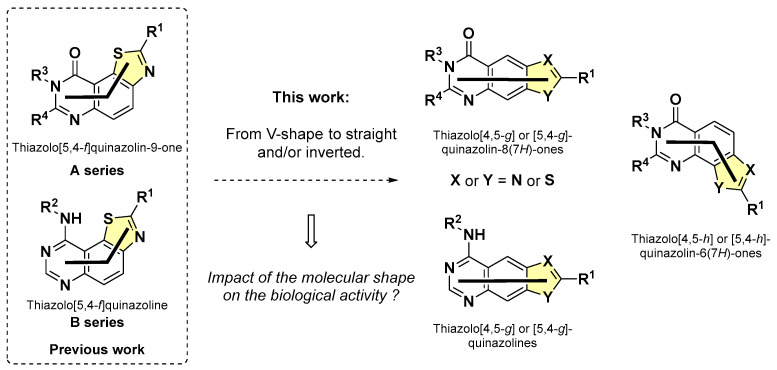
Straight thiazole-fused [4,5-*g*] or [5,4-*g*]quinazolin-8-ones and quinazolines and inverted thiazolo[4,5-*h*] or [5,4-*h*]quinazolin-8-one derivatives.

Recently, we described the regioselective cyclization of *N*-aryl cyanothioformanilide intermediates for the synthesis of novel polyfunctionalized benzothiazoles [12]. This innovative protocol may make it possible to obtain regioisomeric analogues of thiazolo[5,4-*f*]quinazolines and quinazolinones. In fact, the synthesis of straight thiazole-fused [4,5-*g*] or [5,4-*g*]quinazolin-8-one and quinazoline derivatives has hitherto not been reported. This motivated us to study the chemical access and biological properties of these novel heterocyclic systems and also to explore the synthesis of inverted thiazolo[4,5-*h*] or [5,4-*h*]quinazolin-8-one derivatives for SAR studies (Figure 2).

**Figure 3:**
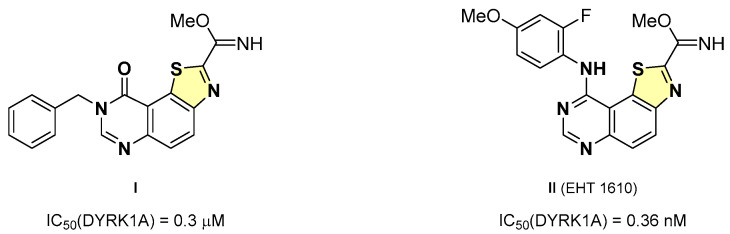
Structures of methyl 8-benzyl-9-oxo-8,9-dihydrothiazolo[5,4-*f*]quinazoline-2-carbimidate (**I**) and methyl 9-[(2-fluoro-4-methoxyphenyl)amino]thiazolo[5,4-*f*]quinazoline-2-carbimidate (**II**) used as references in this study.

Among the numerous compounds studied, the angular thiazolo[5,4-*f*]quinazolin-9-one **I** exhibits submicromolar IC_50_ values for DYRK1A kinase [12] (Figure 3). Thiazolo[5,4-*f*]quinazoline **II** (also known as EHT 1610) is particularly effective and shows excellent affinity for the DYRK family, with IC_50_ values in the nanomolar range [6,7,8]. It has therefore been used as a specific inhibitor of DYRK1A in various studies designed to demonstrate the impact of this kinase in various biological phenomena [13,14,15] (Figure 3).

These two compounds were the starting point for these new SAR investigations, which aimed to assess whether modifying the molecular form and position of certain atoms (e.g., *N* and *S* of the thiazole ring) of this type of compound may have a significant effect on their biological activity and therapeutic potential.

## 2. Results and Discussion

### 2.1. Chemistry

#### 2.1.1. Synthesis of Thiazole-Fused Quinazolinones (A Series)

**Scheme 1:**
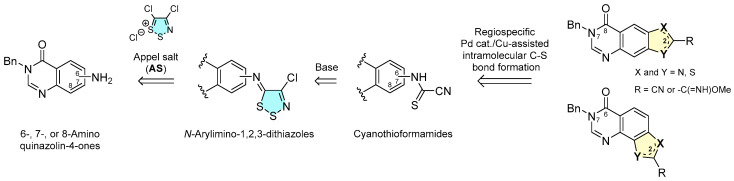
Retrosynthetic strategy for the synthesis of the novel thiazole-fused quinazolinones.

Across all retrosynthetic approaches, 6-, 7- or 8-amino-3-benzylquinazolin-4(3*H*)-ones, which can react with Appel salt, were designed as ideal starting materials. The resulting *N*-arylmino-1,2,3-dithiazoles can be converted into the corresponding *N*-aryl cyanothioformamides under basic conditions. Lastly, the target thiazole-fused quinazolinones may be obtained via intramolecular C-S bond formation as previously described for the synthesis of various 2-cyanobenzothiazoles (Scheme 1) [11].

**Scheme 2:**
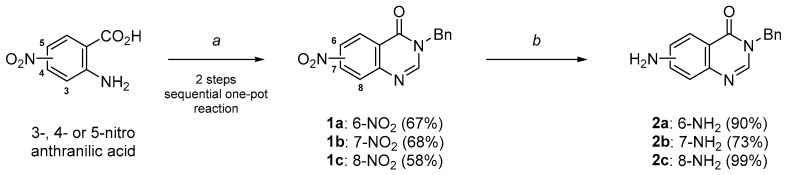
Synthesis of aminoquinazolinones **2a**, **2b** and **2c** (isolated yields)**.** Conditions: (a) 1. DMF-DMA (2.5 equiv) in EtOAc (1 M), 70 °C (Mw), 30 min; 2. Benzylamine (1.5 equiv) in AcOH (1 M), 118 °C (Mw), 30 min. (b) Pd/C (10% *w*/*w*), HCO_2_NH_4_ (5.0 equiv) in EtOH (0.2 M), reflux, 1 h.

The first part of this study then focused on the synthesis of both 7- and 8-amino-3-benzylquinazolin-4(3*H*)-ones **2b** and **2c**, according to the procedure already developed for **2a** [16] (Scheme 2).

In a sequential multi-component reaction (MCR) process, a solution of 3- or 4-nitroanthranilic acid (1.0 equiv) and DMF-DMA (5.0 equiv) in ethylacetate (EtOAc) (1 M) was heated for 30 min at reflux (77 °C), under microwave irradiation (Mw). After removing the solvent under reduced pressure, AcOH (0.1 M) and benzyl amine (1.5 equiv) were added and the resulting mixture was irradiated at reflux (118 °C) for 30 min. After workup, the nitro-3-benzylquinazolin-4(3*H*)-ones (**1b** and **1c**) were isolated in 58–68% yields. Reduction of the nitro group using ammonium formate (NH_4_CO_2_H, 5.0 equiv) in the presence of a catalytic amount of palladium on carbon (Pd/C) gave the amino-3-benzylquinazolin-4(3*H*)-ones **2a**, **2b** and **2c** with an overall yield of 60%, 50% and 57%, respectively, for the three steps (Scheme 2).

**Scheme 3:**
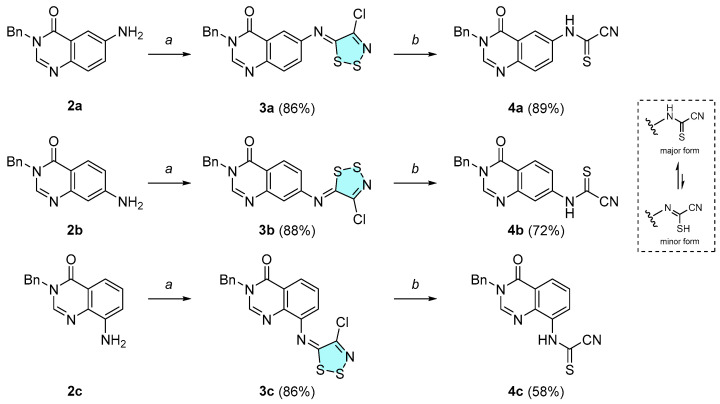
Conditions (isolated yields): (a) Appel salt (1.1 equiv), pyridine (2.0 equiv) in CH_2_Cl_2_ (0.3 M), rt, 1 h; (b) DBU (3.0 equiv) in dry CH_2_Cl_2_ (0.2 M), rt, 15 min. The ^1^H NMR spectra of compounds **4-c** show two tautomeric forms in which the thione is the most abundant (ratio major/minor = 90:10; for more details, see the Supplementary Material).

Due to their unique structures, synthetic challenges and potentially interesting biological properties, the synthesis of new isomers of fused heterocyclic compounds was studied using amino quinazolinones **2a**–**c**. Appel salt (1.1 equiv) and pyridine (2.0 equiv) were added to a solution of aromatic amine (**2a**, **2b** or **2c**) in dichloromethane (0.3 M), and the resulting mixture was stirred at room temperature (rt) for 1 h. After workup, the corresponding imino-1,2,3-dithiazoles **3a**, **3b** and **3c** were isolated in very good yields (86%, 88% and 86%, respectively). Next, these compounds were treated with 1,8-diazabicyclo[5.4.0]undec-7-ene (DBU) (3 equiv) [11,17] to provide the expected cyanothioformamides **4a**, **4b** and **4c** in excellent to good yields (89%, 72% and 58%, respectively) (Scheme 3).

**Scheme 4:**
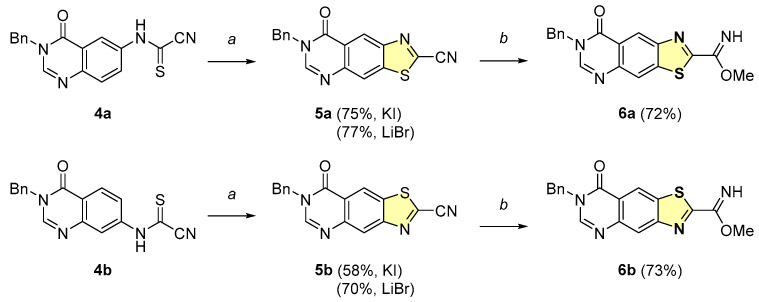
Synthesis of the regioisomeric thiazole-fused quinazolinones **6a** and **6b** (isolated yields). Conditions: (a) PdCl_2_ (20 mol%), CuI (50 mol%), KI or LiBr (2.0 equiv) in DMSO/DMF 1:1 (0.025 M), air, 120 °C, 4 h. (b) NaOMe (1.0 equiv) in MeOH (0.07 M), 50 °C (Mw), 1 h.

As part of our recent investigations on the synthesis of polyfunctionalized benzothiazoles [12], the starting cyanothioformamides (e.g., **4** series) were mixed with PdCl_2_ (20 mol%) and CuI (50 mol%) in DMSO/DMF (1:1, *v*/*v*) and heated in the presence of air (open vessel) at 120 °C for 4 h. In our previous work, it was demonstrated that the addition of an inorganic additive, such as KI or LiBr, was necessary to achieve regiospecific fusion of the thiazole ring with the aromatic moiety. While the presence of KI was particularly effective in the synthesis of mono-, di- or tri-alkylated or halogenated benzothiazoles [11], LiBr proved to be more efficient for the synthesis of anthranilic and anthranilonitrile derivatives (Scheme 4) [12].

Therefore, for the synthesis of thiazoloquinazolin-8-ones **5a** and **5b**, the same amount of KI or LiBr (2.0 equiv) was added to the reaction mixtures and their respective efficiency was evaluated. The results depicted in Scheme 4 confirm the effectiveness of LiBr when the starting cyanothioformamides have both nitrogen and carbonyl functional groups, even when integrated into a pyrimidinone-type structure. LiBr was particularly effective in the synthesis of **5b** with a 70% yield compared with 58% in the presence of KI. At the late stage of the synthetic process, the nitrile function of the two thiazole-fused quinazolinones **5a** and **5b** was transformed into methylcarbimidate after heating (50 °C) in a solution of sodium methoxide (1.0 equiv) in MeOH (0.07 M) [6]. The methyl 7-benzyl-thiazolo[4,5-*g*]quinazoline-2-carbimidate (**6a**) and its thiazolo[5,4-*g*]quinazoline analogue (**6b**) were isolated in good yields (72% and 73%, respectively) (Scheme 4).

**Scheme 5:**
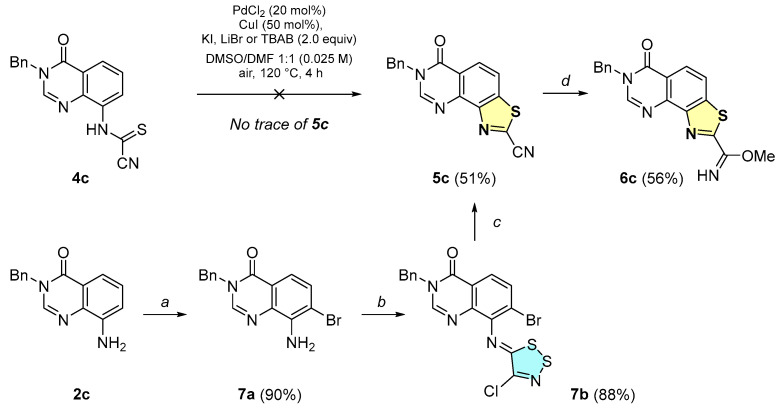
Conditions (isolated yields): (a) NBS (1.0 equiv), DMF (0.1 M), rt, 2 h. (b) Appel salt (1.1 equiv), pyridine (2.0 equiv) in CH_2_Cl_2_ (0.3 M), rt, 2 h. (c) CuI (2.0 equiv) in pyridine (0.25 M), 115 °C (Mw), 30 min. (d) NaOMe (1.0 equiv), MeOH (0.07 M), 50 °C (Mw), 1 h.

The procedure described for **5a** and **5b** was also applied to the synthesis of compound **5c** in the hope of yielding the angular isomer **6c**. Whatever the conditions considered, no trace of the desired 7-benzyl-6-oxo-6,7-dihydrothiazolo[5,4-*h*]quinazoline-2-carbonitrile (**5c**) was detected (Scheme 5), while performing the reaction under standard strategies gave **5c** in 40% overall yield. Compound **2c** was brominated to give **7a,** which was subsequently condensed with Appel salt to give the brominated imino-1,2,3-dithiazole **7b** in an overall yield of 79%. Cu-mediated cyclization in hot pyridine (115 °C) yielded **5c** in 51% yield. Thiazolo[5,4-*h*]quinazoline-2-carbonitrile **5c** was then heated at 50 °C in a solution of sodium methoxide (1.0 equiv) in MeOH (0.07 M), leading to the angular methylcarbimidate derivative **6c** in a moderate yield (56%).

**Scheme 6:**
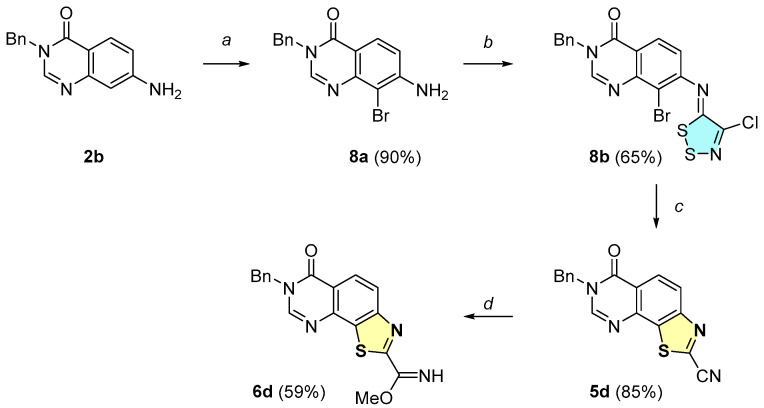
Conditions (isolated yields): (a) NBS (1.0 equiv), DMF (0.1 M), rt, 2 h. (b) Appel salt (1.1 equiv), pyridine (2.0 equiv) in CH_2_Cl_2_ (0.3 M), rt, 2 h. (c) CuI (2.0 equiv) in pyridine (0.25 M), 115 °C (Mw), 30 min. (d) NaOMe (1.0 equiv), MeOH (0.07 M), 50 °C (Mw), 1 h.

The same four-step sequence was successfully applied to the synthesis of compounds **5d** and **6d,** which were obtained in good yields. Here again, the preliminary introduction of a bromine in the *ortho*-position of the aromatic amine (**8a** and **8b**) allowed a regiocontrolled formation of the thiazole moiety (Scheme 6).

#### 2.1.2. Synthesis of Regioisomeric Analogues of **II** (EHT 1610)

**Scheme 7:**
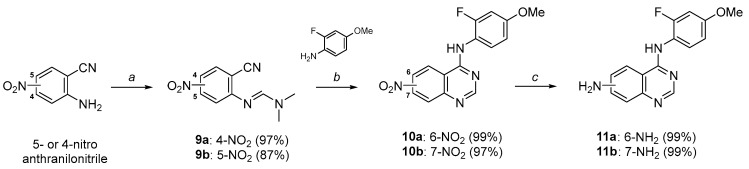
Synthesis of 6- and 7-aminoquinazolines **11a** and **11b** (isolated yields). Conditions: (a) DMFDMA (0.4 M), EtOAc (1 M), 70 °C (Mw), 30 min. (b) 2-fluoro-4-methyoxyaniline (1.5 equiv), AcOH (1 M), 118 °C (Mw), 30 min. (c) Pd/C (10% *w*/*w*), HCO_2_NH_4_ (5.0 equiv), EtOH (0.2 M), reflux, 1 h.

The second part of this work focused on the synthesis of regioisomers of EHT 1610 (**II** in Figure 3), a thiazolo[4,5-*g*]quinazoline derivative with a high affinity for kinases of the DYRK family [6,7,8]. The synthetic route envisioned was based on the process described above and suggested the synthesis of the quinazoline ring prior to its fusion with the thiazole unit. The synthesis of both angular analogues required a reliable procedure to prepare aminoquinazolines **11a** and **11b** from 4- or 5-nitroanthranilonitriles (Scheme 7). In contrast with anthranilic acid esters (Scheme 2), the first step was not a sequential one-pot procedure, and the intermediate amidines **9a** and **9b** were isolated in excellent yields (97% and 87%, respectively). Condensation of 2-fluoro-4-methoxyaniline and reduction of the 6- and 7-nitroquinazolines (**10a** and **10b**) provided the 6- and 7-aminoquinazolines, **11a** and **11b**, in excellent average yields (95% and 85%, respectively).

**Scheme 8:**
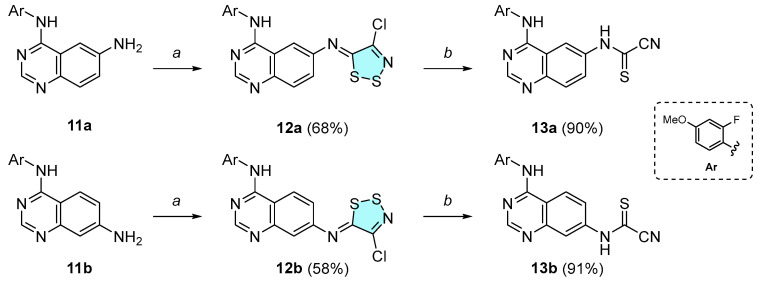
Synthesis of cyanothioformamides **13a** and **13b** (isolated yields). Conditions: (a) In CH_2_Cl_2_ (0.3 M): 1. Appel salt (1.1 equiv), rt, 1 h; 2. DIPEA (2.0 equiv), rt, 1 h. (b) DBU (3.0 equiv) in dry CH_2_Cl_2_ (0.2 M), rt, 15 min. Tautomeric ratio major/minor = 90:10 in favor of the thione form (estimated by ^1^H NMR).

The aminoquinazolines **11a** and **11b** were reacted with Appel salt in the presence of a base. It should be noted that the use of pyridine usually employed in this step gave worse results than in the preceding experiments with quinazolinones (Scheme 8), with yields of 43% and 27% for the synthesis of **12a** and **12b**, respectively. After a brief optimization of the experimental conditions with various bases (e.g., 2,6-lutidine, DABCO, NEt_3_, DBU and DIPEA), it was found that DIPEA allowed for the synthesis of the expected imino-1,2,3-dithiazoles **12a** and **12b** in higher yields (68% and 58%, respectively). Varying the temperature and/or reaction time did not improve the result of this reaction. Cyanothioformamides **13a** and **13b** were then obtained in excellent yields of 90% and 91%, respectively, under the operating conditions described above.

**Scheme 9:**
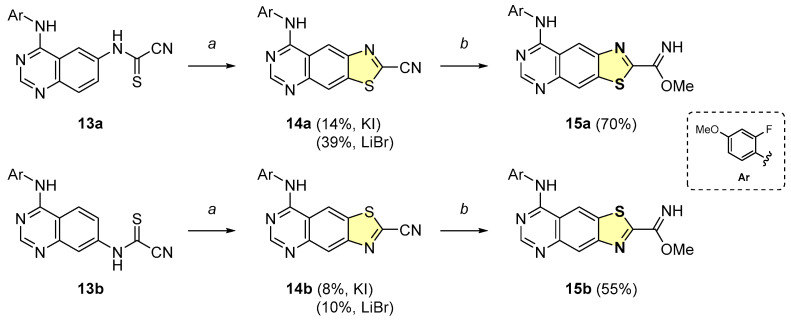
Synthesis of the thiazole-fused quinazolines, **15a** and **15b**, regioisomeric analogues of **EHT 1610** (isolated yields). Conditions: (a) PdCl_2_ (20 mol%), CuI (50 mol%), KI or LiBr (2.0 equiv), DMSO/DMF 1:1 (0.025 M), air, 120 °C, 4 h. (b) NaOMe (1.0 equiv), MeOH (0.07 M), 50 °C (Mw), 1 h.

Regioselective cyclization of **13a** and **13b** was achieved via C–H functionalization/intramolecular and C–S bond formation, catalyzed by PdCl_2_ (20 mol%) and assisted by CuI (50 mol%) in a mixture of DMSO/DMF (1:1; 0.025 M). Thiazole-fused [4,5-*g*] or [5,4-*g*]quinazolines **14a** and **14b** were isolated in 39% and 10% yields, respectively. Whatever the additive used (KI or LiBr), the yield of **14b** was significantly lower than those observed for the quinazolinone derivatives (**5a**, **5b** and **14a**). On the basis of the mechanism suggested in our previous work [11], the poor reactivity can be arguably attributed to a steric hindrance of the aromatic motif (2-fluro-4-methoxyphenyl) hampering the insertion of Pd in the *ortho*-position of the cyanothioformamide group. Since only small amounts of compounds are required for biological investigations, optimization of this step was no longer considered for products **14a** and **14b**. The late stage transformation of the carbonitrile into a methylimidate group was carried out by heating cyanothioformamides (**14a** and **14b**) with sodium methoxide in methanol at 50 °C for 1 h. Both isomers of linear methyl thiazoloquinazoline-2-carbimidates **15a** and **15b** were obtained in good yields (70% and 55%, respectively) (Scheme 9).

### 2.2. Biological Investigations

#### 2.2.1. Kinase Activity Profiling 

The synthesized methylimidate derivatives **6a**–**d**, **15a**–**b** and the reference compounds **I** and **II** were tested at two c*o*ncentrations (10 μM and 1 μM) in the same experimental conditions, against a representative panel of eight mammalian protein kinases of human origin: cyclin-dependent kinase 9 (CDK9/CyclinT), mitotic kinase (Haspin), proviral integration site for Moloney murine leukemia virus kinase (Pim-1), glycogen synthase kinase-3 beta (GSK-3β), casein kinase 1-epsilon (CK-1ε), janus kinase 3 (JAK3), CDC-like kinase 1 (CLK1) and dual specificity tyrosine phosphorylation regulated kinase 1A (DYRK1A) [18].

The results depicted in Table 1 show that compounds **6b** and **6d** had no inhibitory activity against any of the kinases tested, unlike compounds **6a** and **6c,** which had moderate inhibitory activity against JAK3 (41 to 64% depending on the compound and the concentration tested). Compound **6c** also significantly inhibited Haspin kinase activity (61% to 88%, depending on concentration). Compounds **15a** and **15b** moderately inhibited JAK3 kinase activity at all concentrations (around 38% inhibition). In addition to their effect on DYRK1A activity, compounds **I** and **II** (EHT 1610) also inhibited the activities of the other kinases tested. Compound **II** exhibited much greater inhibitory activity than compound **I**, particularly on Haspin (69% to 92% inhibition), Pim-1 (48 to 68% inhibition), GSK-3β (71% to 87 inhibition), CK-1ε (56% to 81% inhibition) and CLK1 (75 to 80% inhibition) kinases.

#### 2.2.2. Cytotoxic Activity Studies

As the potential cytotoxicity of model compounds (**I** and **II** in Figure 3) has never been investigated to date, we tested most of the array of new fused heterocyclic compounds synthesized in this study. This included the cyanated precursors **5a**–**d** as well as **I**-CN, the cyanated precursor of **I**. Thus, the potential in vitro cytotoxic profile of thiazoloquinazolinones (**I**, **I**-CN, **5a**–**d** and **6a**–**d**) and thiazoloquinazolines (**II**, **15a** and **15b**) was investigated on seven tumor cell lines, representative of various human cancers, including liver (Huh7-D12), colon (Caco-2 and HCT-116), breast (MCF-7, MDA-MB-231, MDA-MB-468), prostate (PC-3) and non-cancerous cells (human skin fibroblasts). In all these assays, wide-spectrum anticancer agents such as (*R*)-Roscovitine (Rosco), Doxorubicin (Doxo) and Taxol were used as positive controls (for details, see Supplementary Materials Section).

Firstly, the potential antiproliferative activity was preliminarily assessed in vitro at a single dose of 25 μM for 48 h. Compounds that showed significant inhibitory activity against at least one cell line with a survival percentage < 50%, when compared to DMSO control treatment (set at 100%), were then studied in more detail. Complete dose–response and survival curves were obtained at various concentrations (ranging from 0.1 μM to 25 μM). These data enabled the relative IC_50_ values, representing the concentration of compound that kills 50% of cells after 48 h of incubation [19], to be estimated. The results for all the compounds tested are reported in Table 2.

The results showed that all the thiazole-fused quinazolinones tested (**I**, **5** and **6** series) exhibited a broad spectrum of cytotoxicity against the selected cancer cell lines. The activities measured for compounds (**I**-CN, **5a**, **5c**, **5d** and **I**, **6a**, **6c**, **6d**) were lower than for their analogues **5b** and **6b**, respectively. More precisely, **5c** (IC_50_: 4 µM to 24 µM), **6c** (IC_50_: 3 µM to 13 µM), **5d** (IC_50_: 7 µM to 18 µM) and **6d** (IC_50_: 2 µM to 11 µM) had similar activities, while **5a** (IC_50_: 7 µM to 62 µM) and **6a** (IC_50_: 17 µM to 27 µM) were the less active of this series. Note that this is the sole case showing a marked difference in favor of the imidate compared with its cyanated congener. With IC_50_ values ranging from 12 µM (Caco-2 cells) to 21 µM (MDA-MB-231 and MDA-MB-461cells), **I** was slightly less active than its precursor **I**-CN (IC_50_: 7 µM to 13 µM). However, their activity remained similar to that of the above-mentioned products. Among the molecules tested, **5b** and **6b** were definitely the most active compounds, with IC_50_ values ranging from 1 µM to 3 μM, depending on the cell lines (bold values in Table 2) Interestingly, there was no real difference between cyanated compound **5b** and its methyl imidate derivative **6b**.

In the thiazoloquinazoline series, compound **15b** did not show detectable cytotoxicity within the studied concentration range in either of the cell lines studied. On the contrary, compound **II** (EHT 1610) exhibited cytotoxicity in all cell lines, while the linear derivative **15a** was noticeably active in only three of the lines tested. The highest antiproliferative activity toward the cancer cell lines was observed for compound **II** (IC_50_: 3 μM for Caco-2 cells, 4 µM for PC-3 and HCT-116 cells, 5 µM for Huh7-D12, MCF-7 and MDA-MB-231 and 9 µM for MDA-MB-468 cells). In all cases in which compound **15a** showed some activity, its effectiveness was lower than for **II** (IC_50_: 8 µM for Huh7-D12 and HCT-116 cells to 9 µM for MDA-MB-231 cells or 32 µM for MCF-7 cells). None of the products evaluated in this study showed significant activity against normal cells (fibroblasts). It may be noted the HCT-116 cancer cell line (colon) seemed to be more sensitive than other cells.

## 3. Materials and Methods

### 3.1. Chemistry Work

#### 3.1.1. General Information

All reagents were purchased from commercial suppliers and used without further purification. All reactions were monitored by thin-layer chromatography with aluminum plates (0.25 mm) precoated with silica gel 60 F254 (Merck KGaA, Darmstadt, Germany). Visualization was performed with UV light at a wavelength of 254 nm. Purifications were conducted with a flash column chromatography system (PuriFlash, Interchim, Montluçon, France) using stepwise gradients of petroleum ether (also called light petroleum) (PE) and dichloromethane (DCM) as the eluent. Melting points were measured with an SMP3 Melting Point instrument (STUART, Bibby Scientific Ltd., Roissy, France) with a precision of 1.5 °C. IR spectra were recorded with a Spectrum 100 Series FTIR spectrometer (PerkinElmer, Villebon S/Yvette, France). Liquids and solids were investigated with a single-reflection attenuated total reflectance (ATR) accessory; the absorption bands are given in cm^−1^. NMR spectra (^1^H, ^13^C and ^19^F) were acquired at 295 K using an AVANCE 300 MHz spectrometer (Bruker, Wissembourg, France) at 300, 75 and 282 MHz. Coupling constant J was in Hz and chemical shifts are given in ppm. Mass (ESI, EI and field desorption (FD)) were recorded with an LCP 1er XR spectrometer (WATERS, Guyancourt, France). Mass spectrometry was performed by the Mass Spectrometry Laboratory of the University of Rouen.

The purity of all tested compounds was determined by chromatographic analysis performed at 25 °C on Ultimate 3000 (Thermo Scientific, Les Ulis, France) with a quaternary pump equipped with a photodiode array detector (DAD) managed at 254 nm. Column was a Luna C18 (150 mm × 4.6 mm; 3 μm particle size) provided by Phenomenex (Le Pecq, France). The mobile phase was water (A) and acetonitrile (B) (*v*/*v*); starting condition was 90% A and 10% B in which the solvant B changed from 10% to 90% in 4% by minute. Flow rate was 0.5 mL/min and 5 μL was injected. The purity of all products was >96%.

Microwave (Mw)-assisted reactions were carried out in sealed tubes with a Biotage Initiator microwave synthesis instrument, and temperatures were measured by an IR sensor (Biotage, Uppsala, Sweden). The time indicated in the various protocols is the time measured when the mixtures were at the programmed temperature. The pressure measured in the sealed tubes at the end of the reactions performed never exceeded 10 bar. Some reactions were carried out in 10 mL sealed tubes with Monowave 50 (Anton Paar GmbH, Les Ulis, France). The temperature was monitored by an external contact sensor placed at the cavity bottom, measuring the surface temperature of the reaction vessel. The time indicated in the various protocols is the time measured when the mixtures were at the programmed temperature.

Aminoquinazolinones (**2a**–**c**) and **8a** were obtained using a two-step procedure as described in the main text (Scheme 2 and Scheme 6, respectively). Compounds **7b** and **8b** were synthesized from **7a** and **8a,** which were obtained from **2c** and **2b**, as depicted in Scheme 5 and Scheme 6. Nitro--2-fluoro-4-methoxyphenyl)-quinazolines **10a** and **10b** were synthesized in two steps from the corresponding nitro-anthranilic esters, as shown in Scheme 7. More details are available in Supplementary Materials. Detailed procedures and physicochemical characterization of new products of the series **3**–**6** and **10**–**15** are described below.

#### 3.1.2. Synthesis of [(4-Chloro-5H-1,2,3-dithiazol-5-ylidene]amino)quinazolin-4(3H)-ones **3a**–**c**

To a stirred solution of aminoquinazolinone (**2a**–**c**) (1.0 equiv, 4.0 mmol) in CH_2_Cl_2_ (13.3 mL, 0.3 M), Appel Salt (1.1 equiv, 0.917 g, 4.4 mmol) and pyridine (2.0 equiv, 0.644 mL, 8.0 mmol) were successively added. The resulting mixture was stirred at room temperature for 1 h, after which water (10 mL) was added. The resulting solution was then extracted with CH_2_Cl_2_. The organic phase was washed with brine, dried over MgSO_4_ and concentrated under reduced pressure. The crude product was purified on silica gel with petroleum ether (PE)/CH_2_Cl_2_ (100:0 to 0:100, *v*/*v*) as the eluent to afford the desired products.

3-Benzyl-6-[(4-chloro-5*H*-1,2,3-dithiazol-5-ylidene)amino]quinazolin-4(3*H*)-one *(***3a***)* [16]. Yellow solid (0.264 g, 86%); m.p. 165–166 °C. ^1^H NMR (300 MHz, CDCl_3_) δ 8.20 (d, *J* = 2.4 Hz, 1H), 8.11 (s, 1H), 7.80 (d, *J* = 8.7 Hz, 1H), 7.62 (dd, *J* = 8.7, 2.4 Hz, 1H), 7.41–7.32 (m, 5H), 5.21 (s, 2H).

3-Benzyl-7-[(4-chloro-5*H*-1,2,3-dithiazol-5-ylidene)amino]quinazolin-4(3*H*)-one *(***3b***)*. Yellow solid (1.80 g, 88%); m.p. 186–187 °C. IR (neat) ν_max_: 1670, 1596, 1465, 1430, 1363, 1168, 1153, 882, 861, 842, 744, 703, 691, 542 cm^−1^. ^1^H NMR (300 MHz, CDCl_3_) δ 8.41 (d, *J* = 8.5 Hz, 1H), 8.13 (s, 1H), 7.45 (d, *J* = 2.0 Hz, 1H), 7.38–7.30 (m, 5H), 7.29 (dd, *J* = 8.5, 2.0 Hz, 1H), 5.20 (s, 2H). ^13^C NMR (75 MHz, CDCl_3_) δ 161.1, 160.6, 156.8, 150.0, 147.9, 147.3, 135.7, 129.5, 129.2, 128.6, 128.2, 120.3, 120.1, 116.0, 49.8. HRMS (EI^+^) *m*/*z*, calcd for C_17_H_11_ClN_4_OS [M+H]^+^: 387.0141, found: 387.0135.

3-Benzyl-8-[(4-chloro-5*H*-1,2,3-dithiazol-5-ylidene)amino]quinazolin-4(3*H*)-one *(***3c***).* Brown-orange solid (0.980 g, 86%); m.p. 151–152 °C. IR (neat) ν_max_: 1650, 1603, 1565, 1440, 1322, 1144, 862, 770, 738, 700 cm^−1^. ^1^H NMR (300 MHz, DMSO-d6) δ 8.58 (s, 1H), 8.01 (dd, *J* = 7.8, 1.5 Hz, 1H), 7.60 (t, *J* = 7.8 Hz, 1H), 7.49 (dd, *J* = 7.8, 1.5 Hz, 1H), 7.43–7.24 (m, 5H), 5.20 (s, 2H). ^13^C NMR (75 MHz, DMSO-d6) δ 162.01, 159.92, 147.94, 147.58, 145.92, 137.96, 136.63, 128.66 (2C), 128.00, 127.74, 127.71 (2C), 123.70, 123.15, 123.02, 49.05. HRMS (EI^+^) *m*/*z*, calcd for C_17_H_12_N_4_OS_2_^35^Cl [M+H]^+^: 387.0141, found: 387.0156.

#### 3.1.3. Synthesis *N*-Arylcyanothioformamides **4a**–**c**

To a stirred solution of **3a**–**c** (1.0 equiv, 2.0 mmol) in dry CH_2_Cl_2_ (10 mL, 0.2 M), DBU (3.0 equiv, 0.896 mL, 6.0 mmol) was added. The resulting mixture was stirred under argon at room temperature for 15 min, after which NaHSO_4_ 1 M (10 mL) was added. The resulting solution was extracted with CH_2_Cl_2_. The organic phase was washed with brine, dried over MgSO_4_ and concentrated under reduced pressure. The crude product was purified on silica gel with PE/CH_2_Cl_2_ (50:50 to 0:100, *v*/*v*) as the eluent to afford the desired products **4a**–**c**.

(3-Benzyl-4-oxo-3,4-dihydroquinazolin-6-yl)carbamothioyl cyanide *(***4a***).* Pale orange solid (0.074 g, 89%, ratio major/minor 90:10); m.p. 212–213 °C. IR (neat) ν_max_: 3254, 2864, 2224 (CN), 1694, 1601, 1483, 1372, 1331, 1106, 947, 842, 732, 693, 608, 521 cm^−1^. ^1^H NMR (300 MHz, DMSO-d6), δ 13.75 (s, 1H, major and minor), 8.94 (d, *J* = 2.4 Hz, 0.9H, major), 8.64 (s, 0.1H, minor), 8.63 (s, 0.9H, major), 8.24 (d, *J* = 2.4 Hz, 0.1H, minor), 8.16 (dd, *J* = 8.8, 2.6 Hz, 0.1H, minor), 7.95 (dd, *J* = 8.7, 2.6 Hz, 0.9H, major), 7.81 (dd, *J* = 8.8, 5.0 Hz, 1H, major and minor), 7.40–7.25 (m, 5H, minor and major), 5.20 (s, 2H, major and minor). ^13^C NMR (75 MHz, DMSO-d6) δ 164.9 (minor), 161.5 (major), 159.6 (major and minor), 148.7 (major and minor), 147.1 (minor), 146.7 (major), 136.6 (major and minor), 136.5 (minor), 136.3 (major and minor), 129.2 (minor), 128.9 (major), 128.7 (major and minor), 128.4 (major), 127.8 (major and minor), 127.7 (major and minor), 122.2 (minor), 121.8 (major), 119.7 (minor), 119.0 (major), 114.6 (minor), 113.8 (major), 49.1 (major and minor). HRMS (EI^+^) *m*/*z*, calcd for C_17_H_13_N_4_OS [M+H]^+^: 321.0797, found: 321.0806.

(3-Benzyl-4-oxo-3,4-dihydroquinazolin-7-yl)carbamothioyl cyanide *(***4b***).* Pale orange solid (0.241 g, 72%, ratio major/minor 85:15); m.p. 207–208 °C. IR (neat) ν_max_: 2893, 1701, 1613, 1483, 1406, 1370, 1170, 1144, 848, 736, 699, 542 cm^−1^. ^1^H NMR (300 MHz, DMSO-d6) δ 13.81 (s, 1H, major and minor), 8.64 (s, 0.15H, minor), 8.62 (s, 0.85H, major), 8.46 (d, *J* = 1.9 Hz, 0.85H, major), 8.22 (d, *J* = 8.7 Hz, 1H, major and minor), 7.90 –7.84 (m, 1H, major and minor), 7.62 (dd, *J* = 8.6, 2.1 Hz, 0.15H, minor), 7.40 –7.26 (m, 5H, major and minor), 5.20 (s, 2H, major and minor). ^13^C NMR (75 MHz, DMSO-d6) δ 165.3 (minor), 162.5 (major), 159.5 (minor), 159.4 (major), 149.4 (minor), 149.2 (major), 148.8 (minor), 148.6 (major), 142.7 (minor), 142.5 (major), 136.7 (major and minor), 128.7 (2C, major and minor), 127.9 (minor), 127.7 (major), 127.7 (2C, major and minor), 127.5 (major and minor), 121.4 (major and minor), 120.5 (minor), 120.2 (major), 120.1 (minor), 119.4 (major), 113.7 (major), 112.7 (minor), 49.0 (major and minor). HRMS (EI^+^) *m*/*z*, calcd for C_17_H_13_N_4_OS [M+H]^+^: 321.0797, found: 321.0800.

(3-Benzyl-4-oxo-3,4-dihydroquinazolin-8-yl)carbamothioyl cyanide *(***4c***).* Orange solid (0.360 g, 58%, ratio major/minor 70:30); m.p. 153–154 °C. IR (neat) ν_max_: 3124, 1677, 1619, 1525, 1359, 1109, 763 cm^−1^. ^1^H NMR (300 MHz, DMSO-d6) δ 13.55 (s, 1H, major and minor), 8.77 (s, 0.3H, minor), 8.72 (s, 0.7H, major), 8.27–8.21 (m, 1H, major and minor), 8.18 (dd, *J* = 8.0, 1.4 Hz, 0.7H, major), 8.00 (dd, *J* = 7.7, 1.4 Hz, 0.3H, minor), 7.65 (q, *J* = 8.0 Hz, 1H, major and minor), 7.41–7.27 (m, 5H, major and minor), 5.23 (s, 2H, major and minor). ^13^C NMR (75 MHz, DMSO-d6) δ 167.86 (minor), 164.23 (major), 159.50 (major), 159.39 (minor), 149.01 (minor), 148.71 (major), 142.94 (minor), 142.18 (major), 136.52 (major), 136.44 (minor), 134.04 (minor), 132.41 (major), 131.00 (major), 130.81 (minor), 128.67 (major, 2C), 127.78 (minor, 2C), 127.72 (major, 2C), 127.28 (minor), 126.90 (minor, 2C), 126.16 (major), 122.79 (minor), 122.65 (major), 113.76 (major), 49.27 (minor), 49.17 (major). HRMS (EI^+^) *m*/*z*, calcd for C_17_H_13_N_4_OS [M+H]^+^: 312.0810, found: 312.0807.

#### 3.1.4. Synthesis of Thiazoloquinazolinones **5a** and **5b**

PdCl_2_ (10 mol %, 17.7 mg, 0.1 mmol), CuI (0.5 equiv, 47.6 mg, 0.25 mmol), KI (2.0 equiv, 166 mg, 1.0 mmol) and carbamothioyl cyanide **4a** or **4b** (1.0 equiv, 0.5 mmol) were suspended in a mixture of anhydrous DMF/DMSO 1:1 (20 mL, 0.025 M). The resulting mixture was stirred at 120 °C for 4 h. The resulting solution was then diluted with AcOEt and washed 3 times with water, washed with brine, dried over MgSO_4_ and concentrated under reduced pressure. The crude product was purified on silica gel (PE/CH_2_Cl_2_ 50:50 to 0:100, *v*/*v*) to afford the desired cyanated products **5a** or **5b**.

7-Benzyl-8-oxo-7,8-dihydrothiazolo[4,5-g]quinazoline-2-carbonitrile *(***5a***)*. White solid (0.076 g, 77%); m.p. 237–238 °C. IR (neat) ν_max_: 3100, 2238 (CN), 1671, 1607, 1402, 1326, 1267, 1118, 881, 842, 756, 715, 702, 524, 420 cm^−1^. ^1^H NMR (300 MHz, CDCl_3_) δ 9.19 (s, 1H), 8.31 (s, 1H), 8.18 (s, 1H), 7.42–7.33 (m, 5H), 5.25 (s, 2H). ^13^C NMR (75 MHz, CDCl_3_) δ 160.9, 150.9, 147.5, 146.8, 141.1, 138.8, 135.4, 129.3 (2C), 128.8, 128.3 (2C), 124.7, 122.7, 120.7, 112.6, 50.0HRMS (EI^+^) *m*/*z*, calcd for C_17_H_11_N_4_OS [M+H]^+^: 319.0654, found: 319.0663.

7-Benzyl-8-oxo-7,8-dihydrothiazolo[5,4-g]quinazoline-2-carbonitrile *(***5b***).* White solid (0.165 g, 70%); m.p. 217–218 °C. IR (neat) ν_max_: 3100, 2238 (CN), 1671, 1607, 1402, 1326, 1133, 1118, 1075, 842, 702, 524, 420 cm^−1^. ^1^H NMR (300 MHz, CDCl_3_) δ 8.99 (s, 1H), 8.56 (s, 1H), 8.18 (s, 1H), 7.41 –7.33 (m, 5H), 5.24 (s, 2H). ^13^C NMR (75 MHz, CDCl_3_) δ160.5, 155.8, 146.9, 146.8, 141.6, 135.4, 133.9, 129.3 (2C), 128.7, 128.2 (2C), 123.8, 122.7, 121.7, 112.5, 50.0. HRMS (EI^+^) *m*/*z*, calcd for C_17_H_11_N_4_OS [M+H]^+^: 319.0654, found: 319.0663.

#### 3.1.5. Synthesis of Thiazoloquinazolinones **5c** and **5d**

A suspension of brominated iminodithiazole **7b** or **8b** (1.0 equiv), copper(I) iodide (2.0 equiv) in pyridine (0.3 M) was heated at 115 °C (Mw) for 30 min. After cooling, the resulting solution was dissolved in EtOAc and washed with a sodium thiosulfate solution. The organic layer was dried over MgSO_4_, and the solvent was removed in vacuo. The crude product was purified by flash chromatography (DCM-EtOAc, 9:1) to afford the desired products **5c** and **5d**.

7-Benzyl-6-oxo-6,7-dihydrothiazolo[5,4-*h*]quinazoline-2-carbonitrile (**5c**). Beige powder (0.024 g, 51%); m.p. 273–274 °C. IR (neat) ν_max_: 3067, 2227, 1661, 1593, 1453, 1366, 1131, 798, 742, 699 cm^−1^. ^1^H NMR (300 MHz, DMSO-d6) δ 8.89 (s, 1H), 8.44–8.32 (m, 2H), 7.43–7.26 (m, 5H), 5.29 (s, 2H). ^13^C NMR (75 MHz, DMSO-d6) δ 159.63, 150.22, 147.52, 143.89, 141.85, 137.84, 136.49, 128.69, 127.81, 127.77, 125.67, 121.21, 120.50, 113.41, 49.43. HRMS (EI^+^) *m*/*z*, calcd for C_17_H_11_N_4_OS [M+H]^+^: 319.0654, found: 319.0658.

*7-Benzyl-6-oxo-6,7-dihydrothiazolo[4,5-h]quinazoline-2-carbonitrile (***5d***)* was obtained as previously described [16]. Beige solid (85%). ^1^H NMR (300 MHz, DMSO-d6) δ 8.49 (d, *J* = 8.8 Hz, 1H), 8.28 (s, 1H), 8.23 (d, *J* = 8.8 Hz, 1H), 7.39–7.38 (m, 5H), 5.27 (s, 2H).

#### 3.1.6. Synthesis of Imidates **6a**–**d**

The cyanated quinazolinone **5a**–**d** (1.0 equiv, 50.0 mg, 0.14 mmol) was added to a solution of sodium methoxide (1.2 equiv, 9.2 mg, 0.17 mmol) in MeOH (2.0 mL, 0.07 M). The resulting mixture was heated at 50 °C (Mw) for 1 h. The resulting solution was then concentrated and Et_2_O was added. The precipitate was filtered and purified by column chromatography (DCM/MeOH 100 to 95:5) to afford the desired products **6a**–**d**.

Methyl 7-benzyl-8-oxo-7,8-dihydrothiazolo[4,5-*g*]quinazoline-2-carbimidate *(***6a***).* White powder (0.079 g, 72%); m.p. 213–214 °C. IR (neat) ν_max_: 3288, 2941, 1686, 1645, 1595, 1520, 1349, 1206, 1137, 1070, 818, 754, 697 cm^−1^. ^1^H NMR (300 MHz, DMSO-d6) δ 9.59 (s, 1H), 8.81 (s, 1H), 8.65 (s, 1H), 8.60 (s, 1H), 7.46–7.21 (m, 6H), 5.24 (s, 2H), 3.96 (s, 3H). ^13^C NMR (75 MHz, DMSO-d6) δ 160.74, 160.32, 159.31, 150.75, 148.21, 145.25, 141.89, 136.74, 128.71 (2C), 127.79 (3C), 121.64, 121.28, 121.19, 54.27, 49.05. HRMS (EI^+^) *m*/*z*, calcd for C_18_H_15_N_4_O_2_S [M+H]^+^: 351.0916, found: 351.0917.

Methyl 7-benzyl-8-oxo-7,8-dihydrothiazolo[5,4-*g*]quinazoline-2-carbimidate *(***6b***).* White powder (0.065 g, 73%); m.p. 244–245 °C. IR (neat) ν_max_: 3221, 3093, 2942, 1673, 1650, 1607, 1431, 1323, 1276, 1129, 1067, 853, 741 cm^−1^. ^1^H NMR (300 MHz, DMSO-d6) δ 9.60 (s, 1H), 9.10 (d, *J* = 0.6 Hz, 1H), 8.63 (s, 1H), 8.39 (d, *J* = 0.6 Hz, 1H), 7.39–7.30 (m, 5H), 5.25 (s, 2H), 3.96 (s, 3H). ^13^C NMR (75 MHz, DMSO-d6) δ 159.39, 148.43, 147.96, 146.08, 136.81, 136.68, 134.57, 128.70, 127.73, 127.70, 127.65, 122.86, 122.51, 122.04, 121.10, 54.29, 48.93HRMS (EI^+^) *m*/*z*, calcd for C_18_H_15_N_4_O_2_S [M+H]^+^: 351.0916, found: 351.0916.

Methyl 7-benzyl-6-oxo-6,7-dihydrothiazolo[5,4-*h*]quinazoline-2-carbimidate *(***6c***).* White powder (0.062 g, 56%); m.p. 213–214 °C. IR (neat) ν_max_: 3296, 1669, 1642, 1601, 1501, 1436, 1337, 1125, 1076, 740, 695, 636 cm^−1^. ^1^H NMR (300 MHz, DMSO-d6) δ 9.44 (s, 1H), 8.84 (s, 1H), 8.32 (d, *J* = 8.7 Hz, 1H), 8.25 (d, *J* = 8.7 Hz, 1H), 7.42–7.26 (m, 5H), 5.29 (s, 2H), 3.98 (s, 3H). ^13^C NMR (75 MHz, DMSO-d6) δ 159.85, 159.72, 158.89, 149.51, 147.92, 143.55, 141.70, 136.64, 128.69 (2C), 127.76, 127.68 (2C), 123.98, 121.45, 120.02, 54.22, 49.30. HRMS (EI^+^) *m*/*z*, calcd for C_18_H_15_N_4_O_2_S [M]^+^: 351.0916, found: 351.0930.

Methyl 7-benzyl-6-oxo-6,7-dihydrothiazolo[4,5-*h*]quinazoline-2-carbimidate *(***6d***).* White powder (0.065 g, 59%); m.p. 205–206 °C. IR (neat) ν_max_: 3271, 2953, 1669, 1603, 1453, 1326, 1149, 1062, 896, 794, 520 cm^−1^. ^1^H NMR (300 MHz, DMSO-d6) δ 9.57 (s, 1H), 8.85 (s, 1H), 8.28–8.19 (m, 2H), 7.43–7.24 (m, 5H), 5.27 (s, 2H), 3.97 (s, 3H). ^13^C NMR (75 MHz, DMSO-d6) δ 161.71, 159.64, 159.25, 155.81, 150.33, 144.44, 136.50, 132.75, 128.68 (2C), 127.78, 127.73 (2C), 124.97, 122.43, 118.81, 54.26, 49.44. HRMS (EI^+^) *m*/*z*, calcd for C_18_H_15_N_4_O_2_S [M+H]^+^: 351.0916, found: 351.0919.

#### 3.1.7. Synthesis of Compounds **10a** and **10b**

The 2-cyano-4- or 5-nitrophenyl)-*N,N*-dimethylformimidamide **9a** or **9b** (1.0 equiv) was dissolved in AcOH (1 M) and 2-fluoro-4-methoxyaniline (1.5 equiv) was added. The reaction mixture was heated at 118 °C (Mw) for 10 min. The resulting solution was diluted with CH_2_Cl_2_ and neutralized with a saturated solution of NaHCO_3_ and Na_2_CO_3_ (to pH = 8–9). The organic layer was washed twice with water (150 mL) and brine, dried over MgSO_4_ and concentrated under reduced pressure. The crude product was purified on silica gel by column chromatography (DCM 100: 0 to DCM/EtOAc 50:50, *v*/*v*) to afford the desired products **10a** or **10b**.

N-(2-Fluoro-4-methoxyphenyl)-6-nitroquinazolin-4-amine *(***10a***)*.^.^ Orange powder (99%, 1.60 g); m.p. 186–187 °C. IR (neat) ν_max_: 3413, 3401, 3099, 1620, 1586, 1527, 1512, 1362, 1342, 1285, 1114, 1024, 829, 746, 523, 448 cm^−1^. ^1^H NMR (300 MHz, CDCl_3_) δ 9.02 (d, *J* = 2.3 Hz, 1H), 8.83 (s, 1H), 8.56 (dd, *J* = 9.2, 2.4 Hz), 8.16 (s, 1H), 8.01 (d, *J* = 9.2 Hz, 1H), 7.92 (t, *J* = 9.2 Hz, 1H), 6.82–6.73 (m, 2H), 3.83 (s, 3H). ^13^C NMR (75 MHz, CDCl_3_) δ 159.4, 158.8 (d, *J* = 10.5 Hz), 158.2, 156.3 (d, *J* = 246.8 Hz), 153.3, 145.0, 130.5, 126.9 (d, *J* = 2.9 Hz), 126.8, 118.6, 117.8 (d, *J* = 11.9 Hz), 114.3, 110.1 (d, *J* = 3.1 H), 102.3 (d, *J* = 23.2 Hz), 55.9. HRMS (EI^+^) *m*/*z*, calcd for C_15_H_12_N_4_O_3_F [M+H]^+^: 315.0893, found: 315.0883.

N-(2-Fluoro-4-methoxyphenyl)-7-nitroquinazolin-4-amine *(***10b***).* Orange powder (97%, 1.31 g); m.p. 217–218 °C. IR (neat) ν_max_: 3442, 2974, 2901, 1607, 1546, 1525, 1504, 1357, 1280, 1203, 1156, 1104, 1060, 808, 740, 523 cm^−1^_._ ^1^H NMR (300 MHz, DMSO-d6) δ 10.13 (s, 1H), 8.71 (d, *J* = 9.1 Hz, 1H), 8.59 (s, 1H), 8.51 (d, *J* = 2.4 Hz, 1H), 8.36 (dd, *J* = 9.1, 2.4 Hz, 1H), 7.42 (t, *J* = 8.9 Hz, 1H), 6.99 (dd, *J* = 12.2, 2.4 Hz, 1H), 6.86 (ddd, *J* = 8.8, 2.8, 1.1 Hz, 1H), 3.82 (s, 3H). ^13^C NMR (75 MHz, DMSO-d6) δ 159.02, 159.00 (d, *J* = 10.0 Hz), 157.91 (d, *J* = 162.4 Hz), 156.04, 150.13, 149.56, 129.33, 125.67, 122.98, 119.62, 118.27, 117.96 (d, *J* = 12.9 Hz), 110.21 (d, *J* = 3.0 Hz), 102.22 (d, *J* = 23.8 Hz), 55.83. HRMS (EI^+^) *m*/*z*, calcd for C_15_H_12_N_4_O_3_F [M+H]^+^: 315.0893, found: 315.0899.

#### 3.1.8. Compounds **11a** and **11b**

##### Synthetized from **10a** and **10b**, Following the General Procedure Described for the Cyanothioformamides **4a**–**c**

N^4^-(2-Fluoro-4-methoxyphenyl)quinazoline-4,6-diamine *(***11a***).* White powder (99%, 1.15 g); m.p. 166–167 °C. IR (neat) ν_max_: 3352, 1625, 1576, 1513, 1422, 1216, 1152, 1026, 846, 833, 466, 391 cm^−1^. ^1^H NMR (300 MHz, CDCl_3_) δ 8.59 (s, 1H), 8.29 (t, *J* = 9.3 Hz, 1H), 7.75 (d, *J* = 8.9 Hz, 1H), 7.22 (dd, *J* = 8.9, 2.4 Hz, 1H), 7.12 (s, 1H), 6.93 (d, *J* = 2.4 Hz, 1H), 6.81–6.73 (m, 2H), 3.82 (s, 3H). ^13^C NMR (75 MHz, CDCl_3_) δ 156.8, 156.6 (d, *J* = 59.4 Hz), 154.9 (d, *J* = 243.9 Hz), 151.9, 145.3, 144.2, 130.4, 125.0 (d, *J* = 2.3 Hz), 123.9, 120.0 (d, *J* = 10.6 Hz), 116.4, 109.7 (d, *J* = 3.2 Hz), 102.1 (d, *J* = 23.3 Hz), 101.0, 55.9. HRMS (EI^+^) *m*/*z*, calcd for C_15_H_14_FN_4_O_3_, [M+H]^+^: 285.1152, found: 285.1145.

N^4^-(2-Fluoro-4-methoxyphenyl)quinazoline-4,7-diamine *(***11b***)*. White powder (99%, 1.15 g); m.p. 280–281 °C. IR (neat) ν_max_: 3675, 2988, 2901, 1511, 1405, 1148, 1066, 1056, 850, 562 cm^−1^. ^1^H NMR (300 MHz, DMSO-d6) δ 9.16 (s, 1H), 8.15 (s, 1H), 8.06 (d, *J* = 8.9 Hz, 1H), 7.34 (t, *J* = 8.9 Hz, 1H), 6.92 (dd, *J* = 12.2, 2.9 Hz, 1H), 6.86 (dd, *J* = 8.9, 2.3 Hz, 1H), 6.80 (ddd, *J* = 8.8, 2.9, 1.1 Hz, 1H), 6.68 (d, *J* = 2.2 Hz, 1H), 5.99 (s, 2H), 3.79 (s, 3H). ^13^C NMR (75 MHz, DMSO-d6) δ 159.42, 158.31, 157.93, 154.84, 152.89, 151.82, 129.50, 124.02, 119.36, 116.46, 109.81, 105.61, 102.16, 101.84, 55.73. HRMS (EI^+^) *m*/*z*, calcd for C_15_H_14_FN_4_O_3_, [M+H]^+^: 285.1152, found: 285.1156.

#### 3.1.9. Compounds **12a** and **12b**

##### Synthetized from **11a** and **11b**, Following the General Procedure Described for Quinazolines **3a**–**c**, with DIPEA Instead of Pyridine

N^6^-(4-Chloro-5*H*-1,2,3-dithiazol-5-ylidene)-N^4^-(2-fluoro-4-methoxyphenyl)quinazoline-4,6-diamine *(***12a***).* Orange powder (0.354 g, 68%); m.p. 181–182 °C. IR (neat) ν_max_: 3675, 3464, 2971, 2901, 2206, 1566, 1531, 1409, 1277, 1202, 1064, 1034, 871, 750, 651, 501, 454 cm^−1^. ^1^H NMR (300 MHz, CDCl_3_) δ 8.76 (s, 1H), 8.24 (t, *J* = 9.3 Hz, 1H), 8.02 (d, *J* = 8.8 Hz, 1H), 7.74–7.65 (m, 2H), 7.33 (s, 1H), 6.84–6.73 (m, 2H), 3.83 (s, 3H). ^13^C NMR (75 MHz, CDCl_3_) δ 166.4, 160.4, 157.6, 157.6, 157.4, 155.3 (d, *J* = 244.0 Hz), 154.8, 149.2, 131.2, 125.7, 125.5 (d, *J* = 1.9 Hz), 119.1 (d, *J* = 10.8 Hz), 116.0, 110.6, 109.8 (d, *J* = 3.1 Hz), 102.1 (d, *J* = 23.1 Hz), 55.9. HRMS (EI^+^) *m*/*z*, calcd for C_17_H_15_ClFN_5_OS_2_ [M+H]^+^: 420.0156, found: 420.0148.

N^6^-(4-Chloro-5*H*-1,2,3-dithiazol-5-ylidene)-N^4^-(2-fluoro-4-methoxyphenyl)quinazoline-4,7-diamine *(***12b***).* Yellow powder (0.372 g, 58%); m.p. 185–186 °C. IR (neat) ν_max_: 1583, 1535, 1493, 1418, 1307, 1207, 1027, 882, 838, 652, 540 cm^−1^. ^1^H NMR (300 MHz, DMSO-d6) δ 9.73 (s, 1H), 8.54 (d, *J* = 8.8 Hz, 1H), 8.42 (s, 1H), 7.51–7.34 (m, 3H), 6.97 (dd, *J* = 12.1, 2.8 Hz, 1H), 6.89–6.81 (m, 1H), 3.81 (s, 3H). ^13^C NMR (75 MHz, DMSO-d6) δ 161.89, 159.42, 158.80, 157.39 (d, *J* = 187.9 Hz), 155.56, 155.41, 151.04, 146.66, 129.47, 125.61, 119.97, 118.50 (d, *J* = 13.3 Hz), 114.59, 112.43, 110.05 (d, *J* = 2.9 Hz), 102.14 (d, *J* = 24.0 Hz), 55.79. HRMS (EI^+^) *m*/*z*, calcd for C_17_H_12_N_5_OFS_2_^35^Cl [M+H]^+^: 420.0156, found: 420.0156.

#### 3.1.10. Compounds **13a** and **13b**

##### Synthetized from **12a** and **12b**, Following the General Procedure Described for Quinazolines **4a**–**c**

(4-[(2-Fluoro-4-methoxyphenyl)amino]quinazolin-6-yl)carbamothioyl cyanide *(***13a***).* Red powder (0.380 g, 90%); m.p. 210–211 °C. IR (neat) ν_max_: 3677, 2972, 2904, 1600, 1575, 1511, 1392, 1263, 1066, 832, 574 cm^−1^. ^1^H NMR (300 MHz, DMSO-d6) δ 10.33 (s, 1H), 8.67–8.45 (m, 2H), 7.98 (dd, *J* = 8.9, 2.1 Hz, 0.85H, major), 7.90 (s, 0.15H, minor), 7.82 (d, *J* = 8.9 Hz, 1H), 7.41 (t, *J* = 8.9 Hz, 1H), 6.99 (dd, *J* = 12.1, 2.7 Hz, 1H), 6.86 (dd, *J* = 8.8, 2.7 Hz, 1H), 3.81 (s, 3H). ^13^C NMR (75 MHz, DMSO-d6) δ 161.82, 159.43, 159.17, 157.46 (d, *J* = 236.8 Hz), 152.82, 130.66, 129.20, 125.07, 118.23, 117.78, 116.40, 115.81, 114.31, 110.22 (d, *J* = 3.0 Hz), 102.20 (d, *J* = 23.6 Hz), 55.84. ^19^F NMR (282 MHz, DMSO-d6) δ −116.79 (major), −116.89 (minor). HRMS (EI^+^) *m*/*z*, calcd for C_17_H_13_N_5_OFS [M+H]^+^: 354.0825, found: 354.0824.

(4-[(2-Fluoro-4-methoxyphenyl)amino]quinazolin-7-yl)carbamothioyl cyanide *(***13b***).* Red powder (0.267 g, 91%); m.p. 199–200 °C. IR (neat) ν_max_: 3675, 2988, 2901, 1603, 1561, 1539, 1401, 1359, 1228, 1066, 906 cm^−1^. ^1^H NMR (300 MHz, DMSO-d6) δ 14.17 (s, 1H), 10.70 (s, 1H), 8.65 (s, 1H), 8.49 (d, *J* = 8.9 Hz, 1H), 7.93 (s, 1H), 7.64 (d, *J* = 8.9 Hz, 1H), 7.43 (t, *J* = 8.9 Hz, 1H), 7.03 (dd, *J* = 12.2, 2.8 Hz, 1H), 6.89 (dd, *J* = 8.9, 2.8 Hz, 1H), 3.82 (s, 3H). ^13^C NMR (75 MHz, DMSO-d6) δ 161.81, 159.66, 159.50, 159.36, 157.58 (d, *J* = 247.3 Hz), 152.48, 143.14, 129.40, 129.36, 124.36, 117.29 (d, *J* = 13.0 Hz), 112.85, 110.34 (d, *J* = 3.0 Hz), 109.73, 102.23 (d, *J* = 23.6 Hz), 55.89 (major), 54.93 (minor). ^19^F NMR (282 MHz, DMSO-d6) δ −116.92 (minor), −116.98 (major). HRMS (EI^+^) *m*/*z*, calcd for C_17_H_13_N_5_OFS [M+H]^+^: 354.0825, found: 354.0827.

#### 3.1.11. Compounds **14a** and **14b** Were Synthetized from **13a** and **13b**, Following the General Procedure Described for Quinazolines **5a**–**c**, Using LiBr Instead of KI as an Additive

8-[(2-Fluoro-4-methoxyphenyl)amino]thiazolo[4,5-*g*]quinazoline-2-carbonitrile *(***14a***).* Yellow powder (0.068 g, 39%); m.p. 259–260 °C. IR (neat) ν_max_: 2925, 1560, 1535, 1415, 1198, 1035, 842, 792 cm^−1^. ^1^H NMR (300 MHz, DMSO-d6) δ 10.18 (s, 1H), 9.48 (s, 1H), 8.76 (s, 1H), 8.53 (s, 1H), 7.45 (t, *J* = 8.9 Hz, 1H), 7.00 (dd, *J* = 12.2, 2.7 Hz, 1H), 6.91–6.83 (m, 1H), 3.82 (s, 3H). ^13^C NMR (75 MHz, DMSO-d6) δ 159.97, 159.34, 159.02, 157.70 (d, *J* = 247.1 Hz), 155.86, 149.36, 147.60, 139.72, 129.43 (d, *J* = 2.8 Hz), 121.84, 119.63, 118.10 (d, *J* = 12.8 Hz), 114.90, 113.29, 110.18 (d, *J* = 2.8 Hz), 102.20 (d, *J* = 23.8 Hz), 55.82. HRMS (EI^+^) *m*/*z*, calcd for C_17_H_11_N_5_OFS [M+H]^+^: 352.0668, found: 352.0670.

8-[(2-Fluoro-4-methoxyphenyl)amino]thiazolo[5,4-*g*]quinazoline-2-carbonitrile *(***14b***).* Yellow powder (0.040 g, 10%); m.p. 255–256 °C. IR (neat) ν_max_: 2922, 1557, 1532, 1459, 1409, 1350, 1282, 1122, 1028, 849, 796 cm^−1^. ^1^H NMR (300 MHz, DMSO-d6) δ 10.08 (s, 1H), 9.36 (s, 1H), 8.67 (s, 1H), 8.55 (s, 1H), 7.45 (t, *J* = 8.8 Hz, 1H), 7.05–6.96 (m, 1H), 6.87 (d, *J* = 8.8 Hz, 1H), 3.82 (s, 3H). ^13^C NMR (75 MHz, DMSO-d6) δ 159.20, 158.91, 157.34 (d, *J* = 214.2 Hz), 155.42, 155.36 (3C), 154.02, 141.95, 132.58, 129.11 (d, *J* = 3.2 Hz), 122.48, 118.28, 118.27 (d, *J* = 13.0 Hz), 112.99, 110.18 (d, *J* = 2.9 Hz), 102.21 (d, *J* = 23.6 Hz), 55.81. HRMS (EI^+^) *m*/*z*, calcd for C_17_H_11_N_5_OFS [M+H]^+^: 352.0668, found: 352.0676.

#### 3.1.12. Compounds **15a** and **15b** Were Synthetized from **14a** and **14b**, Following the General Procedure Described for Quinazolines **6a**–**c**

Methyl 8-[(2-fluoro-4-methoxyphenyl)amino]thiazolo[4,5-*g*]quinazoline-2-carbimidate *(***15a***).* Pale yellow powder (0.038 g, 70%); m.p. 246–247 °C. IR (neat) ν_max_: 3444, 3270, 1653, 1601, 1556, 1530, 1410, 1348, 1146, 1036, 948, 856, 847, 539 cm^−1^. ^1^H NMR (300 MHz, DMSO-d6) δ 10.06 (s, 1H), 9.52 (s, 1H), 9.34 (s, 1H), 8.63 (s, 1H), 8.49 (s, 1H), 7.45 (m, 1H), 6.99 (m, 1H), 6.87 (dd, *J* = 8.9, 2.7 Hz, 1H), 3.99 (s, 3H), 3.82 (s, 3H). ^13^C NMR (75 MHz, DMSO-d6) δ 160.46, 159.84, 159.60, 158.92, 157.44 (d, *J* = 201.5 Hz), 155.16, 150.37, 146.84, 140.73, 129.49 (d, *J* = 2.9 Hz), 121.51, 118.44, 118.29, 114.31, 110.10 (d, *J* = 2.7 Hz), 102.19 (d, *J* = 23.8 Hz), 55.81, 54.31. ^19^F NMR (282 MHz, DMSO-d6) δ −116.67. HRMS (EI^+^) *m*/*z*, calcd for C_18_H_15_N_5_O_2_FS [M+H]^+^: 384.0930, found: 384.0924.

Methyl 8-[(2-fluoro-4-methoxyphenyl)amino]thiazolo[5,4-*g*]quinazoline-2-carbimidate *(***15b***)*. Pale yellow powder (0.030 g, 55%); m.p. 239–240 °C. IR (neat) ν_max_: 3296, 1647, 1600, 1546, 1511, 1464, 1391, 1325, 1269, 1147, 1095, 949, 847, 832, 539 cm^−1^. ^1^H NMR (300 MHz, DMSO-d6) δ 9.97 (s, 1H), 9.69 (s, 1H), 9.30 (s, 1H), 8.51 (s, 2H), 7.44 (t, *J* = 9.0 Hz, 1H), 7.03–6.95 (m, 1H), 6.86 (d, *J* = 9.0 Hz, 1H), 3.98 (s, 3H), 3.82 (s, 3H). ^13^C NMR (75 MHz, DMSO-d6) δ 162.80, 159.21, 158.76, 155.10, 133.57, 129.14, 121.46, 118.10, 110.14, 102.36, 102.05 55.81, 54.33. HRMS (EI^+^) *m*/*z*, calcd for C_18_H_15_N_5_O_2_FS [M+H]^+^: 384.0930, found: 384.0945.

### 3.2. Kinase Inhibition Assays

Kinase enzymatic activities were assayed in 384-well plates using the ADP-Glo^TM^ assay kit (Promega, Madison, WI, USA) according to the recommendations of the manufacturer. This luminescent ADP detection assay was described by Zegzouti and co-workers and provides a homogeneous and high-throughput screening method to measure kinase activity by quantifying the amount of ADP produced during a kinase reaction [19]. Briefly, the reactions were carried out in a final volume of 6 µL for 30 min at 30 °C in an appropriate kinase buffer (10 mM MgCl_2_, 1 mM EGTA, 1 mM DTT, 25 mM Tris-HCl pH 7.5, 50 µg/mL heparin), with peptide or protein as substrate in the presence of 10 µM ATP. Peptide substrates were obtained from ProteoGenix (Schiltigheim, France) or Sigma for Histone H1, Poly (L-glutamic acid–L-tyrosine) sodium salt, Casein and MBP. After that, 6 µL of ADP-Glo^TM^ Kinase Reagent was added to stop the kinase reaction. After an incubation time of 50 min at room temperature (rt), 12 µL of Kinase Detection Reagent was added for one hour at rt. The transmitted signal was measured using the Envision (PerkinElmer, Waltham, MA, USA) microplate luminometer and expressed as Relative Light Units (RLUs). Kinase activities are expressed as percentages of remaining kinase activities detected after treatment with 1 or 10 µM of the tested compounds. The maximum kinase activity was measured in the absence of inhibitor but with an equivalent dose of DMSO (solvent of the tested compounds).

### 3.3. Cytotoxic Activity

#### 3.3.1. Cell Culture

Skin normal fibroblastic cells were purchased from Lonza (Basel, Switzerland). Huh7-D12, Caco-2, HCT-116, MDA-MB-231, MDA-MB-468, MCF-7 and PC-3 cancer cell lines were obtained from ATCC (American Type Culture Collection). Cells were grown at 37 °C, 5% CO_2_ in ATCC recommended media: DMEM for Huh7-D12, MDA-MB-231, MDA-MB-468 and fibroblastic cells, EMEM for MCF-7 and Caco-2, McCoy’s for HCT-116 and RPMI for PC-3 cells. All culture media were supplemented by 10% of FBS, 1% of penicillin–streptomycin and 2 mM glutamine.

#### 3.3.2. Cytotoxic Assay

Chemicals were solubilized in DMSO at a concentration of 10 mM (stock solution) and diluted in culture medium to the desired final concentrations. The dose effect cytotoxic assays (IC_50_ determination) were performed by increasing the concentrations of each chemical (final well concentrations: 0.1 μM, 0.3 μM, 0.9 μM, 3 μM, 9 μM and 25 μM). Cells were plated in 96-well plates (4000 cells/well). Twenty-four hours after seeding, the cells were exposed to chemicals. After 48 h of treatment, the cells were washed in PBS and fixed in cooled 90% ethanol/5% acetic acid for 20 min and the nuclei were stained with Hoechst 33342 (B2261 Merck KGaA, Darmstadt, Germany). Image acquisition and analysis were performed using a Cellomics ArrayScan VTI/HCS Reader (ThermoScientific, Les Ulis, France). The survival percentages were calculated as the percentage of cell number after compound treatment over cell number after DMSO treatment. The relative IC_50_ values were calculated using the curve fitting XLfit 5.5.0.5 (idbs) integrated in Microsoft Excel as an add-on. The four-parameter logistic model or sigmoidal dose–response model describing the sigmoid-shaped response pattern was used (fit = (A + [(B − A)/(1 + [(C/x)^D])]), where fit is the response and X is the tested concentration of the compound. The lower asymptote is A (also referred to as the bottom of the curve or lower plateau or the min) and the upper asymptote is B (also referred to as the top of the curve or upper plateau or the max). The slope of the linear part of the curve is described by the factor D. The parameter C is the concentration corresponding to the response midway between A and B.

## 4. Conclusions

**Figure 4:**
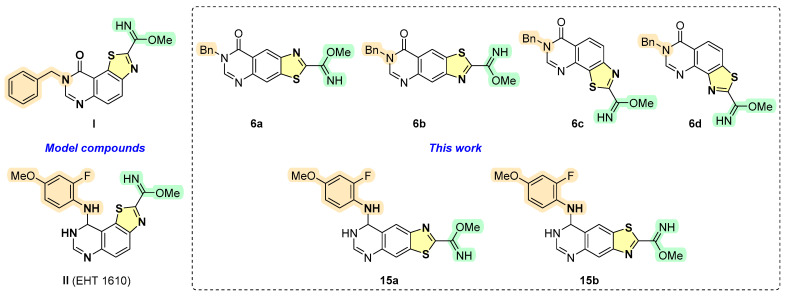
Model compounds **I** and **II** and the synthesized regioisomers **6a**–**d**, **15a** and **15b** hitherto undescribed.

The syntheses of four regioisomers of the model compound **I**, and two of **II** (EHT 1610) were accomplished with the aim of comparing their biological activities (Figure 4). Except for the synthesis of compound **15b**, for which the last two steps need to be optimized, the overall yields are encouraging and suggest the possibility of extending this chemical library.

The results of the biological evaluations carried out during this project confirm that straight (or linear) thiazoloquinazolines are not inhibitors of kinases, and in particular of DYRK1A kinase, which was mainly targeted by our group. Despite these results, we still observed moderate inhibition of JAK3 kinase activity with the thiazoloquinazolinone derivatives **6a** and **6c**, and with the thiazoloquinazoline derivatives **15a** and **15b**.

For the first time, a study of the potential cytotoxicity of such polycyclic compounds was carried out, showing that the majority of them can inhibit the growth of seven cancer cell lines (colon, breast, liver and prostate), with IC_50_ values in the micromolar range. Among the tested thiazoloquinazolinone derivatives (**5** and **6** series), the 7-Benzyl-8-oxo-7,8-dihydrothiazolo[5,4-*g*]quinazolinones **5b** and **6b** proved to be the most potent in stopping the growth of the cancer cell lines tested, with IC_50_ values in the micromolar range. Since these compounds showed no toxicity against normal cells, a larger program of investigations will be launched to investigate the real potential interest of such compounds in anticancer applications.

## Figures and Tables

**Table 1:** Residual activity on a panel of 8 protein kinases ^1,2,3^.

Compound	[C] μM	CDK9/CyclinT	Haspin	Pim-1	GSK-3β	CK-1ε	JAK3	CLK1	DYRK1A
**I**	10	41	45	77	23	70	52	27	21
	1	91	60	79	73	92	48	75	62
**6a**	10	101	105	133	76	69	36	96	103
	1	103	89	80	94	100	55	97	100
**6b**	10	104	82	78	107	103	73	102	109
	1	107	95	90	101	111	92	100	103
**6c**	10	101	12	86	91	87	44	84	68
	1	105	39	74	81	112	59	95	100
**6d**	10	94	51	94	105	98	92	73	63
	1	106	82	92	94	92	80	89	83
**II**	10	59	8	32	13	19	54	20	15
	1	86	31	52	29	44	69	25	16
**15a**	10	100	66	113	70	72	58	52	27
	1	101	83	71	90	95	64	89	72
**15b**	10	110	88	105	104	108	60	105	101
	1	98	80	74	101	98	68	102	95

^1^ For each compound, the residual kinase activity was determined at 10 µM and 1 µM. ^2^ The data mean (n = 2) is expressed as percentage of maximum activity of the DMSO control. ^3^ Values ≥ 100 or close to 100 indicate that the compound cannot inhibit the enzymatic activity at the tested concentrations.

**Table 2:** Results of in vitro cytotoxic activity on a panel of seven cancer cell lines and fibroblasts (IC_50_ μM) ^1,2^.

Compound	Huh7-D12	Caco-2	HCT-116	MCF-7	MBA-MB-231	MBA-MB4-68	PC-3	Fibroblasts
**I-**CN	13	10	7	13	10	10	11	NA
**I**	15	12	18	17	21	21	20	NA ^3^
**5a**	62	10	7	8	40	11	9	NA
**6a**	27	17	20	17	20	19	20	NA
**5b**	3	1	2	2	3	1	2	NA
**6b**	1	2	1	1	3	1	2	NA
**5c**	17	11	4	16	24	10	12	NA
**6c**	10	6	3	7	13	13	8	NA
**5d**	18	9	7	12	14	11	11	NA
**6d**	9	10	2	8	11	5	10	NA
**II**	5	3	4	5	5	9	4	NA
**15a**	8	NA	8	32	9	NA	NA	NA
**15b**	NA	NA	NA	NA	NA	NA	NA	NA
**Rosco** ^4^	11	10	6	7	11	11	10	NA
**Doxo** ^4^	0.03	0.03	0.05	0.05	0.02	0.04	0.08	NA
**Taxol** ^4^	0.016	0.02	0.003	0.006	0.01	0.003	0.004	NA
**DMSO** ^4^	NA	NA	NA	NA	NA	NA	NA	NA

^1^ Relative IC_50_ values are the average of 3 assays. ^2^ For details, see Supplementary Materials (Section S35). ^3^ NA means no significant activity. ^4^ Control experiments.

## Data Availability

Data are contained within the article and Supplementary Materials.

## Supplementary Materials

The following supporting information can be downloaded at: https://www.mdpi.com/article/10.3390/ph17111452/s1: Synthesis of detailed procedures and physicochemical characterization of **1** and **2** series (Sections S3–S4), **7** and **8** series (Sections S4–S5) and **9** series (Section S6). ^1^H NMR and ^13^C NMR spectra of all new compounds (Sections S7–S33). Kinase inhibition assays and cytotoxic evaluation (Section S34–S35).
