# Supplementary material for: Novel Thiazole-Fused [4,5-g] or [5,4-g]Quinazolin-8-ones and Their Quinazoline Analogues: Synthesis and Biological Evaluation
Source: Pharmaceuticals (Basel). 2024 Oct 30;17(11):1452. doi: 10.3390/ph17111452 (PMC11597202; doi:10.3390/ph17111452)
Supplement: Supplementary file 1 [file pharmaceuticals-17-01452-s001.zip › pharmaceuticals-3285900-supplementary.pdf]

pharmaceuticals

# Supplementary Materials

# Novel thiazole-fused [4,5-*g*] or [5,4-*g*]quinazolin-8-ones and their quinazoline analogues: synthesis and biological evaluation

**Nathan Broudic [1], Alexandra Pacheco-Benichou [1], Cécile Corbière[2], Blandine Baratte [3,4], Thomas Robert [3,4], Stéphane Bach [3,4], Hélène Solhi [5], Rémy Le Guével [5], Corinne Fruit [1] and Thierry Besson [1]**

[1] Univ Rouen Normandie, INSA Rouen Normandie, CNRS, COBRA UMR 6014, F-76000 Rouen, France; nathan.broudic@univ-rouen.fr (N.B.); benichoualexandra@yahoo.fr (A.P.-B.); corinne.fruit@univ-rouen.fr (C.F.)

[2] Univ Rouen Normandie, ABTE UR4651, F- 76000 Rouen, France; cecile.corbiere@univ-rouen.fr (C.C.)

[3] Sorbonne Université, CNRS, UMR8227, Integrative Biology of Marine Models Laboratory (LBI2M), Station Biologique de Roscoff, 29680 Roscoff, France

[4] Sorbonne Université, CNRS, FR2424, Plateforme de criblage KISSf (Kinase Inhibitor Specialized Screening facility), Station Biologique de Roscoff, 29680 Roscoff, France ; baratte@sb-roscoff.fr (B.B.); trobert@sb-roscoff.fr (T.R.); stephane.bach@sb-roscoff.fr (S.B.)

[5] Univ Rennes, Plateform ImPACcell, BIOSIT, F-35000 Rennes, France; Helene.solhi@univ-rennes.fr (H.S.); remy.leguevel@univ-rennes.fr (R.LG.)

## Table of contents


| | Pages |
|---|---|
| **General information** | **S2** |
| **General procedures** | **S3-S6** |
| **1H and 13C of new compounds** | **S7-S33** |
| **Kinase inhibition assays** | **S34** |
| **Cytotoxic evaluation** | **S35** |

1. **General information**

All reagents were purchased from commercial suppliers and used without further purification.

All reactions were monitored by thin-layer chromatography with aluminum plates (0.25 mm) precoated with silica gel 60 F254 (Merck KGaA, Darmstadt, Germany). Visualization was performed with UV light at a wavelength of 254 nm.

Purifications were conducted with a flash column chromatography system (PuriFlash, Interchim, Montluçon, France) using stepwise gradients of petroleum ether (also called light petroleum) (PE) and dichloromethane (DCM) as the eluent.

Melting points were measured with an SMP3 Melting Point instrument (STUART, Bibby Scientific Ltd., Roissy, France) with a precision of 1.5 °C.

IR spectra were recorded with a Spectrum 100 Series FTIR spectrometer (PerkinElmer, Villebon S/Yvette, France). Liquids and solids were investigated with a single-reflection attenuated total reflectance (ATR) accessory; the absorption bands are given in $cm^{-1}$.

NMR spectra ($^{1}H$, $^{13}C$ and $^{19}F$) were acquired at 295 K using an AVANCE 300 MHz spectrometer (Bruker, Wissembourg, France) at 300, 75 and 282 MHz. Coupling constant J was in Hz and chemical shifts were given in ppm.

Mass (ESI, EI and field desorption (FD) were recorded with an LCP 1er XR spectrometer (WATERS, Guyancourt, France). Mass spectrometry was performed by the Mass Spectrometry Laboratory of the University of Rouen.

The purity of all tested compounds was determined by chromatographic analysis performed at 25 °C on Ultimate 3000 (Thermo Scientific, Les Ulis, France) with a quaternary pump equipped with a photodiode array detector (DAD) managed at 254 nm. Column was a Luna C18 (150 mm × 4.6 mm; 3 µm particle size) provided by Phenomenex (Le Pecq, France). The mobile phase was water (A) and acetonitrile (B) (v/v); starting condition is 90% A and 10% B in which the solvant B changed to 10% to 90% in 4% by minute. Flow rate was 0.5 mL/min and 5 µL were injected. The percentage of purity of all products was more than 96%.

Microwave-assisted reactions were carried out in sealed tubes with a Biotage Initiator microwave synthesis instrument, and temperatures were measured by an IR sensor (Biotage, Uppsala, Sweden). The time indicated in the various protocols is the time measured when the mixtures were at the programmed temperature. The pressure measured in the sealed tubes at the end of the reactions performed never exceeded 10 bar. Some reactions were carried out in 10 mL sealed tubes with Monowave 50 (Anton Paar GmbH). The temperature was monitored by an external contact sensor placed at the cavity bottom, measuring the surface temperature of the reaction vessel. Time indicated in the various protocols is the time measured when the mixtures were at the programmed temperature.

## 2. General procedures

2.1. Synthesis of N[3]-benzylquinazolinones **1a-c** and **2a-c**

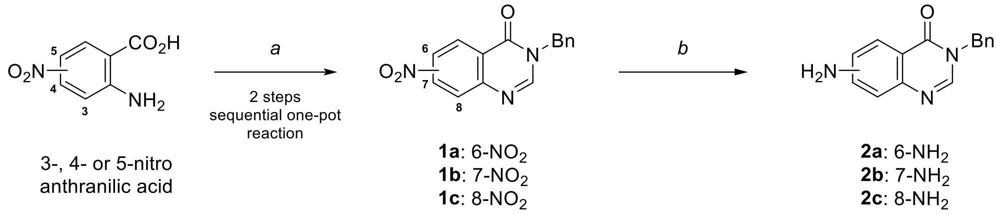


*Method a*) To a solution of 2-amino-3-, 4- or 5-nitrobenzoic acid (1.0 equiv) in EtOAc (1 M) was added DMF-DMA (2.5 equiv for **1a** and **1b**; 5.0 equiv for **1c**). The resulting mixture was heated for 30 min at reflux under microwaves (MW). After removing the solvent, AcOH (1 M) was added, following by benzylamine (1.5 equiv). The resulting mixture was heated for 30 min at reflux (MW) and the solvent was removed partially. The residue was diluted in $CH_2Cl_2$ and water was added. The aqueous phase was then neutralized with a saturated solution of $NaHCO_3$ and solid $Na_2CO_3$ (until pH 8-9). The organic layer was washed twice with brine, dried over $MgSO_4$ and concentrated. The crude product was triturated in EtOAc and the obtained solid was filtrated to afford the expected products **1a**-**c**.

*N[3]-Benzyl-6-nitroquinazolin-4(3H)-one (**1a**)* [16].

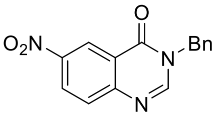

Pale-yellow solid (10.4 g, 67%). **$^{1}$H NMR** (300 MHz, $CDCl_3$) δ 9.23 (d, *J* = 2.6 Hz, 1H), 8.50 (dd, *J* = 9.0, 2.6 Hz, 1H), 8.26 (s, 1H), 7.89 (d, *J* = 9.0 Hz, 1H), 7.39 –7.26 (m, 5H), 5.33 (s, 2H).

*N[3]-Benzyl-7-nitroquinazolin-4(3H)-one (**1b**)* [16].

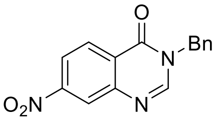

Yellow solid (2.10 g, 68%). **$^{1}$H NMR** (300 MHz, $CDCl_3$) δ 8.54 (d, *J* = 2.2 Hz, 1H), 8.48 (d, *J* = 8.8 Hz, 1H), 8.26 (dd, *J* = 8.8, 2.2 Hz, 1H), 8.21 (s, 1H), 7.77–7.35 (m, 5H), 5.22 (s, 2H).

*N[3]-Benzyl-8-nitroquinazolin-4(3H)-one (**1c**).*

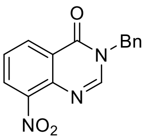

Use of 5.0 equiv of DMA-DMF. Yellow solid (0.526 g, 58%); m.p.: 195 – 196 °C. **IR** (neat) $\nu_{max}$: 1688, 1599, 1526, 1354, 767, 720, 698 $cm^{-1}$. **$^{1}$H NMR** (300 MHz, DMSO-d6) δ 8.74 (s, 1H), 8.38 (dd, J = 7.9, 1.5 Hz, 1H), 8.32 (dd, J = 7.9, 1.5 Hz, 1H), 7.70 (t, J = 7.9 Hz, 1H), 7.42 – 7.27 (m, 5H), 5.22 (s, 2H). **$^{13}$C NMR** (75 MHz, DMSO-d6) δ 158.81, 151.18, 146.52, 139.65, 136.77, 130.76, 128.66 (2C), 127.92, 127.81, 127.76 (2C), 126.95, 123.06, 49.40. HRMS ($EI^+$) m/z, calcd for $C_{15}H_{11}N_3O_3$ $[M+H]^+$: 282.0879, found: 282.0869.

*Method b:* A solution of nitro-quinazolinone **1a-c** (1.0 equiv), ammonium formiate (5.0 eq) and palladium over charcoal (10% wt/wt) in EtOH was heated at reflux for 30 min. The mixture was then filtered over Celite® and the solvent was evaporated. The crude mixture was diluted with EtOAc and neutralised with a solution of $NaHCO_3$ sat. The aqueous phase was extracted 3 times with EtOAc. The organic phases were washed with brine, dried over $MgSO_4$, filtered and concentrated under vaccum to obtain the desired products **2a-c**.

*6-Amino-3-benzylquinazolin-4(3H)-one (**2a**)* [16].

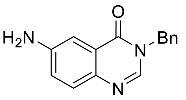


Pale yellow solid (6.81 g, 90%). **$^1$H NMR** (300 MHz, $CDCl_3$) δ 7.92 (s, 1H), 7.53 (d, *J* = 8.7 Hz, 1H), 7.49 (d, *J* = 2.7 Hz, 1H), 7.37-7.27 (m, 5H), 7.10 (dd, J= 8.7, 2.7 Hz, 1H), 5.18 (s, 2H), 3.99, 3.89 (s, 2H).

*7-Amino-3-benzylquinazolin-4(3H)-one (**2b**)* [16].

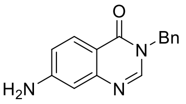


White solid (1.62 g, 73%).**$^1$H NMR** (300 MHz, $CDCl_3$) δ 8.11 (d, *J* = 7.3 Hz, 1H), 8.00 (s, 1H), 7.36 –7.28 (m, 5H), 6.85 –6.77 (m, 2H), 5.15 (s, 2H), 4.23 (s, 2H).

*3-Benzyl-8-amino-quinazolin-4(3H)-one (**2c**).*

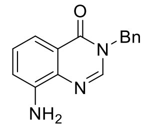


Pale white solid (0.442 g, 99%); m.p. 105 – 106 °C. IR (neat) $\nu_{max}$: 3439, 3341, 1657, 1614, 1353, 757, 741, 701 $cm^{-1}$.$^1$H NMR (300 MHz, DMSO-d6) δ 8.44 (s, 1H), 7.38 – 7.26 (m, 5H), 7.24 (d, *J* = 1.9 Hz, 1H), 7.21 (t, *J* = 7.3 Hz, 1H), 6.98 (dd, *J* = 7.3, 1.9 Hz, 1H), 5.72 (s, 2H), 5.18 (s, 2H). $^{13}$C NMR (75 MHz, DMSO-d6) δ 160.38, 145.00, 144.90, 137.06, 134.77, 128.62 (2C), 127.73, 127.60 (2C), 127.57, 121.93, 115.64, 111.69, 48.72. HRMS (EI$^+$) m/z, calcd for $C_{15}H_{14}N_3O$ [M+H]$^+$: 252.1137, found: 252.1138.

2.2. Synthesis of brominated aminoquinazolinones **7a** and **8a**

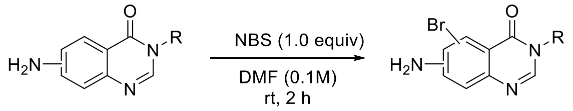


To a solution of amino-quinazolinone (1.0 equiv) in DMF (0.1 M) at rt, *N*-bromosuccinimide (1.0 eq) was added portionwise. The resulting mixture was stirred until reaction completion (monitoring by TLC, 2-4 h). After removal of the solvent, the resulting solution was diluted in $CH_2Cl_2$, the organic layer was washed twice with water and once with brine, dried over $MgSO_4$ and concentrated under reduced pressure to afford the desired product, which is engaged directly into the next step.

To a solution of amino compound (1.0 eq) in DMF (0.1 M) at rt, *N*-bromosuccinimide (1.0 eq) was added portionwise. The resulting mixture was stirred until reaction completion (monitoring by TLC, 2-4 h). After removal of the solvent, the resulting solution was diluted in $CH_2Cl_2$, the organic layer was washed twice with water and once with brine, dried over MgSO4 and concentrated under reduced pressure to afford the desired product which can be engaged directly into the next step.

*8-Amino-3-benzyl-7-bromoquinazolin-4(3H)-one (**7a**).*

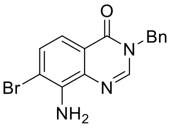

Red solid (0.150 g, 46%); m.p. 121 – 122 °C. IR (neat) $\nu_{max}$: 3360, 1662, 1608, 1588, 1468, 1442, 1405,1356, 1256, 1219, 698 $cm^{-1}$. $^{1}H$ NMR (300 MHz, DMSO-d6) δ 8.56 (s, 1H), 7.51 (d, *J* = 8.6 Hz, 1H), 7.38 – 7.27 (m, 5H), 7.21 (d, *J* = 8.6 Hz, 1H), 5.80 (s, 2H), 5.19 (s, 2H). $^{13}C$ NMR (75 MHz, DMSO-d6) δ 158.17, 147.37, 142.39, 137.04, 136.53, 135.04, 128.64 (2C), 127.70 (3C), 118.37, 108.57, 103.04, 49.33. HRMS ($EI^+$) m/z, calcd for $C_{15}H_{13}N_3O^{79}Br$ $[M+H]^+$: 330.0242, found: 330.0238.

*7-Amino-3-benzyl-8-bromoquinazolin-4(3H)-one (**8a**)* [16]

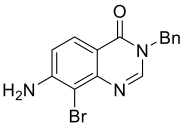

Red solid (0.150 g, 90%). **$^{1}$H NMR** ($CDCl_3$, 300 MHz) δ 8.16 (s, 1H), 8.08 (d, *J* = 8.8 Hz, 1H), 7.35 – 7.34 (m, 5H), 6.89 (d, *J* = 8.8 Hz, 1H), 5.16 (s, 2H), 4.78 (s, 2H).

2.3. Synthesis of [(4-chloro-5*H*-1,2,3-dithiazol-5-ylidene)amino]quinazolinones **7b** and **8b**

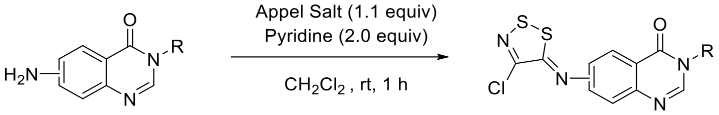


To a solution of aminoquinazolinone **7a** or **8a** (1.0 equiv) in $CH_2Cl_2$ was added Appel Salt (1.1 equiv) and pyridine (2.0 eq). The solution was stirred at rt for 1h. The crude was diluted with water, extracted 3 times with $CH_2Cl_2$. The organic phases were washed with brine, dried over $MgSO_4$, filtered and concentrated under vacuum. The crude product was purified on silica gel by column chromatography (DCM 100:0 to DCM/EtOAc 0:100, v/v) to afford the desired product.

*3-Benzyl-7-bromo-8-[(4-chloro-5H-1,2,3-dithiazol-5-ylidene)amino]quinazolin-4(3H)-one( **7b**).*

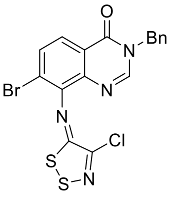

Yellow solid (0.129 g, 61%). **m.p.**: 134 – 135 °C. **IR** (neat) $\nu_{max}$: 3360, 3370, 2200, 1675, 1590, 1442, 1352, 1218, 956, 747, 698, 524 $cm^{-1}$. **$^{1}$H NMR** (300 MHz, DMSO-d6) δ 8.62 (s, 1H), 7.88 (m, 2H), 7.39 – 7.28 (m, 5H), 5.18 (s, 2H). **$^{13}$C NMR** (75 MHz, DMSO-d6) δ 163.83, 160.17, 149.29, 146.47, 145.94, 136.88, 131.80, 129.16, 128.29, 128.26, 124.04, 122.80, 119.48, 49.67. **HRMS** ($EI^+$) m/z, calcd for $C_{17}H_{11}N_4OS_2{}^{35}Cl^{79}Br$ $[M+H]^+$: 465.9246, found: 465.9256.

*3-Benzyl-8-bromo-7-[(4-chloro-5H-1,2,3-dithiazol-5-ylidene)amino]quinazolin-4(3H)-one (**8b**)* [16].

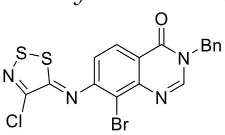

Orange solid (65%). **$^{1}$H NMR** (300 MHz, $CDCl_3$) δ 8.35 (1H, d, *J* = 8.8 Hz), 8.26 (1H, s), 7.38 – 7.33 (m, 5H), 7.18 (d, *J* = 8.8 Hz, 1H), 5.21 (s, 2H).

2.4. Synthesis of *N'*-(2-Cyano-4 or 5-nitrophenyl)-*N,N*-dimethylformimidamides **9a** and **9b.**

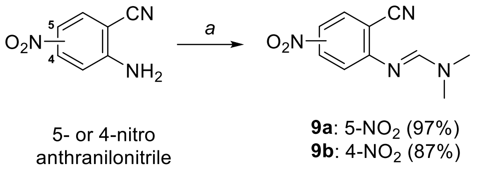


A solution of 2-amino-4- or 5-nitrobenzonitrile (1.0 equiv) in *N,N*-dimethylformamide dimethyl acetal (DMFDMA (0.4 M) was heated for 15 min at 100 °C under microwave irradiations. After cooling the mixture, the precipitate was filtered and washed with $Et_2O$ to afford the desired intermediate. After drying over MgSO4, evaporation of the solvent gave the expected product.

*N'-(2-Cyano-4-nitrophenyl)-N,N-dimethylformimidamide (**9a**)* [5]

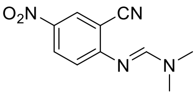


Yellow solid (1.34 g, 97%). **¹H NMR** (300 MHz, $CDCl_3$) δ 8.42 (d, *J* = 2.7 Hz, 1H), 8.24 (dd, *J* = 9.1, 2.7 Hz, 1H), 7.76 (s, 1H), 7.01 (d, *J* = 9.1 Hz, 1H), 3.17 (s, 6H).

*N'-(2-Cyano-5-nitrophenyl)-N,N-dimethylformimidamide (**9b**).*

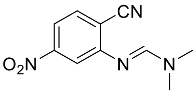


Yellow solid (1.16 g, 87%). **¹H NMR** (300 MHz, DMSO-d6) δ 8.24 (s, 1H), 8.03 (d, *J* = 2.1 Hz, 1H), 7.88 (dd, *J* = 8.5, 0.4 Hz, 1H), 7.73 (dd, *J* = 8.5, 2.2 Hz, 1H), 3.13 (s, 3H), 3.04 (d, *J* = 0.6 Hz, 3H). **¹³C NMR** (75 MHz, DMSO-d6) δ 156.48, 156.33, 150.90, 134.54, 117.27, 115.10, 112.57, 111.38, 34.26 (2C). **HRMS** ($EI^+$) m/z, calcd for $C_{10}H_{11}N_4O_2$ $[M+H]^+$: 219.0882, found: 219.0883.

## 3. $^{1}H$ and $^{13}C$ NMR spectra of new compounds

*N*3-Benzyl-8-nitroquinazolin-4(3H)-one (**1c**).

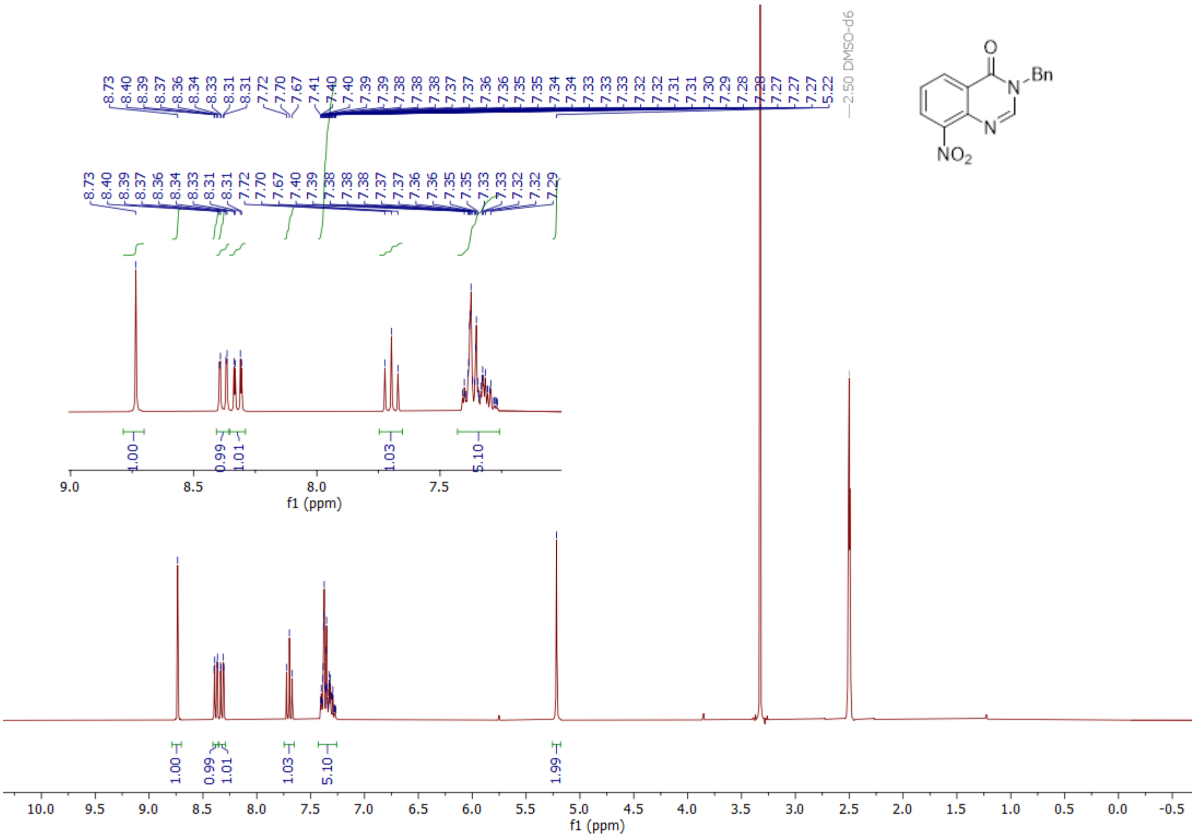

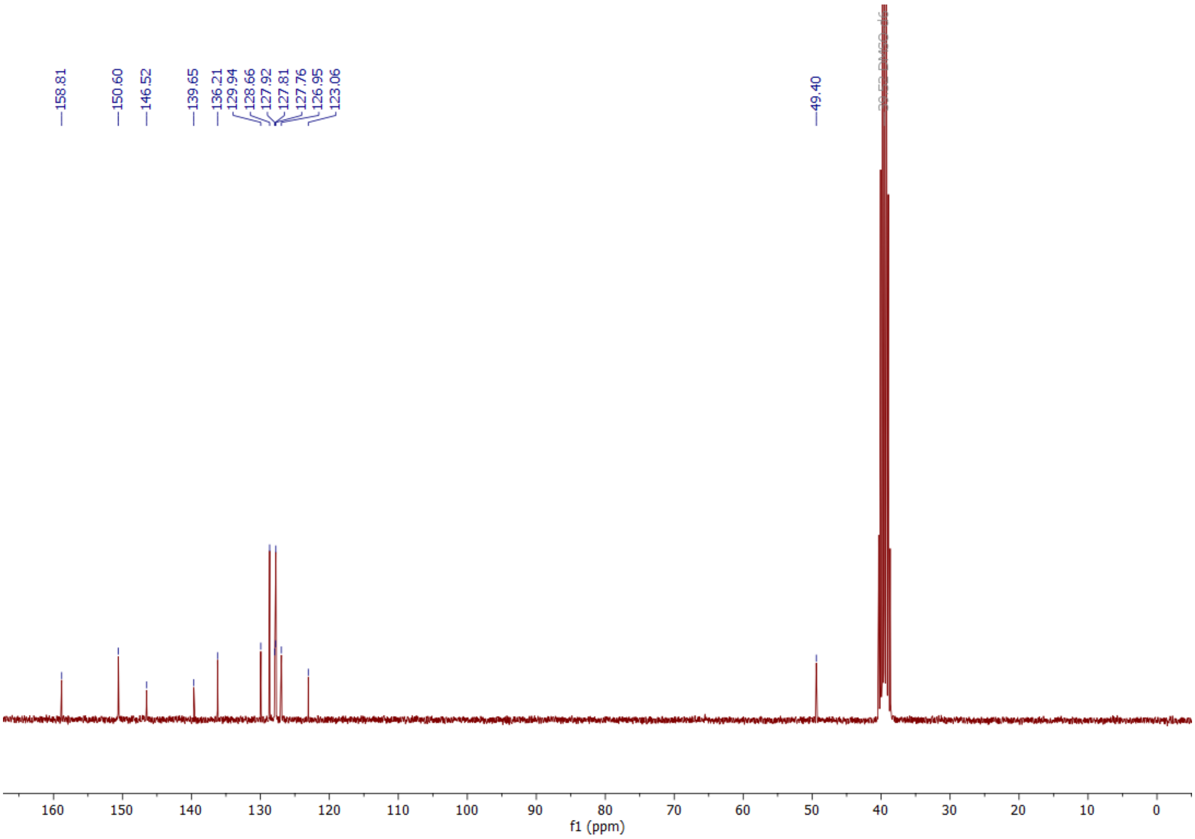

*3-Benzyl-8-amino-quinazolin-4(3H)-one (**2c**).*

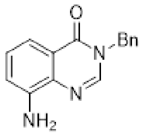


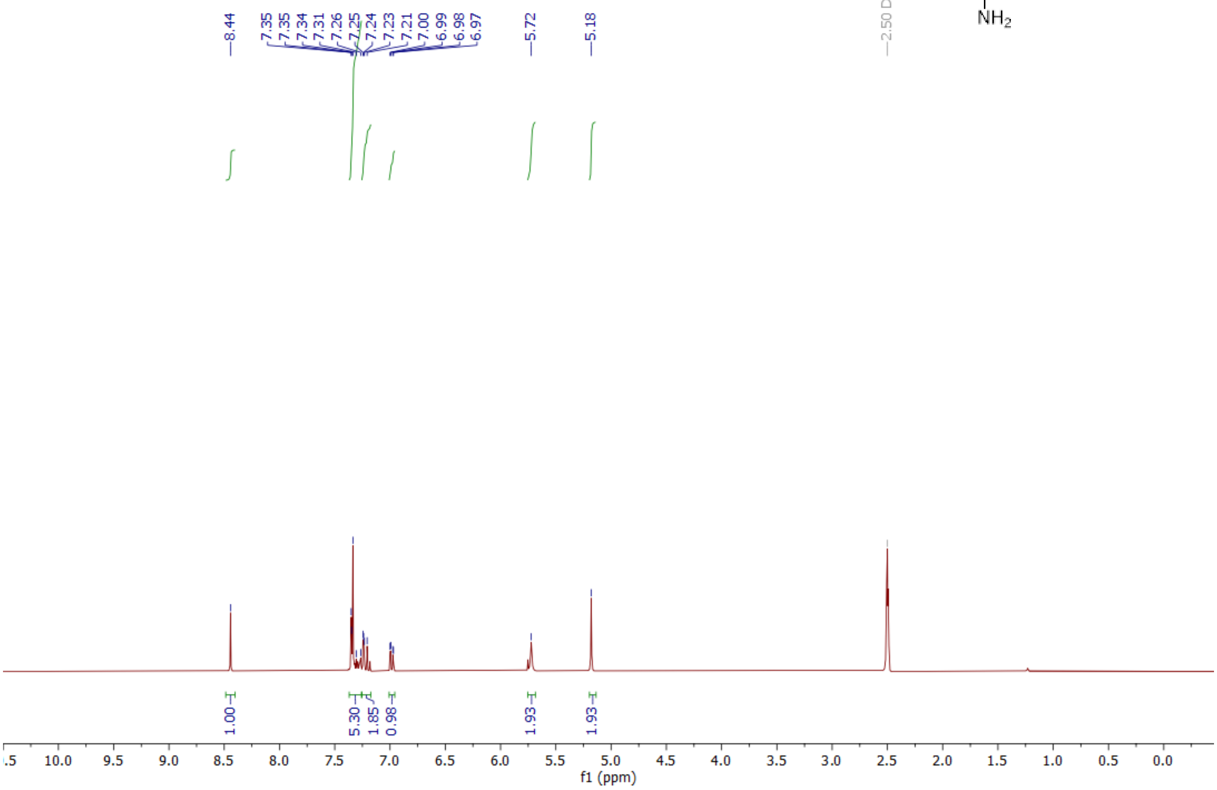


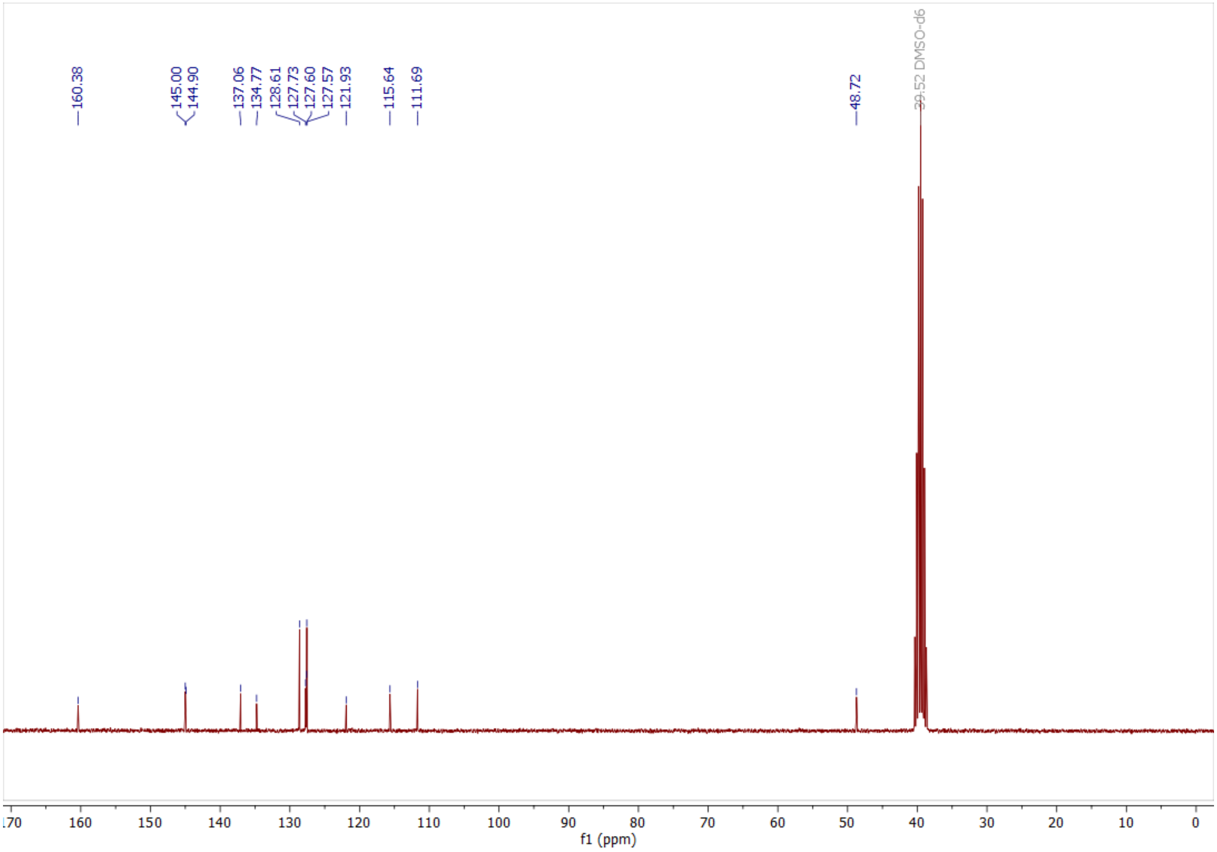

*3-Benzyl-7-[(4-chloro-5H-1,2,3-dithiazol-5-ylidene)amino]quinazolin-4(3H)-one (**3b**).*

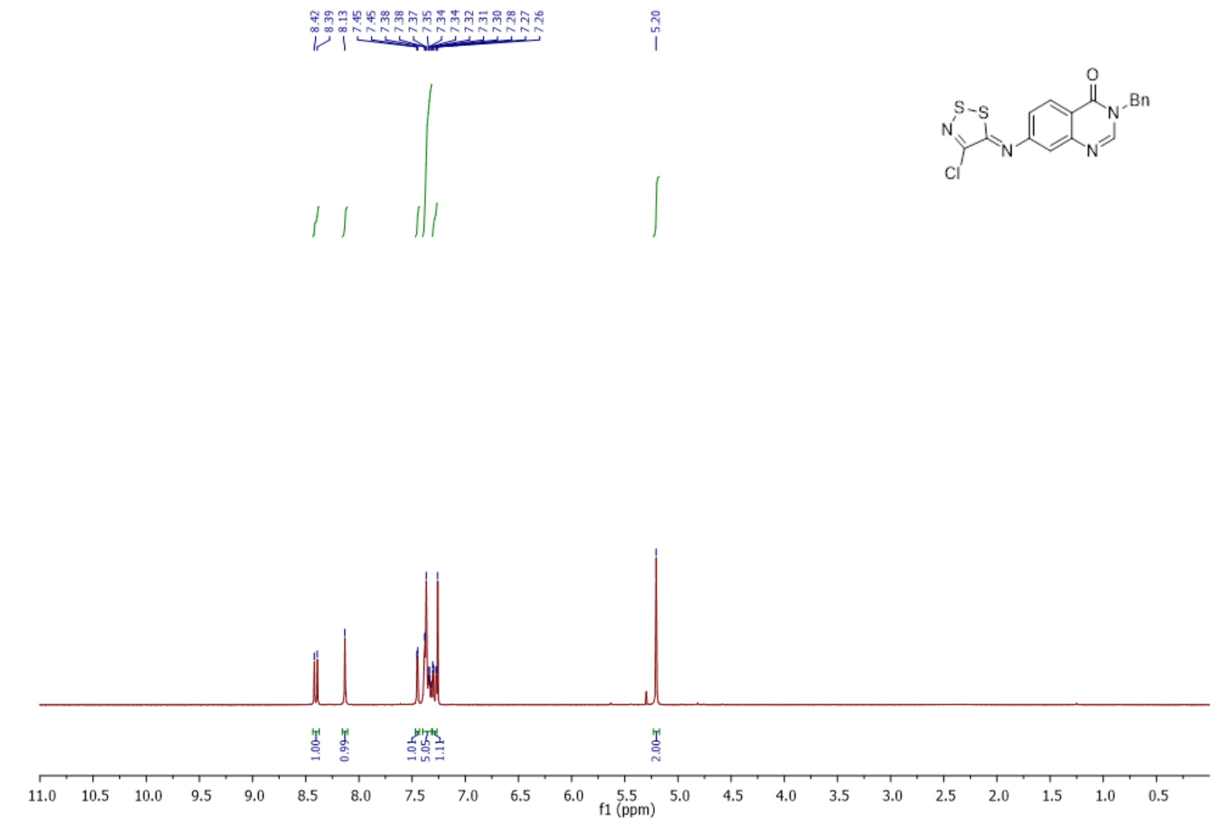


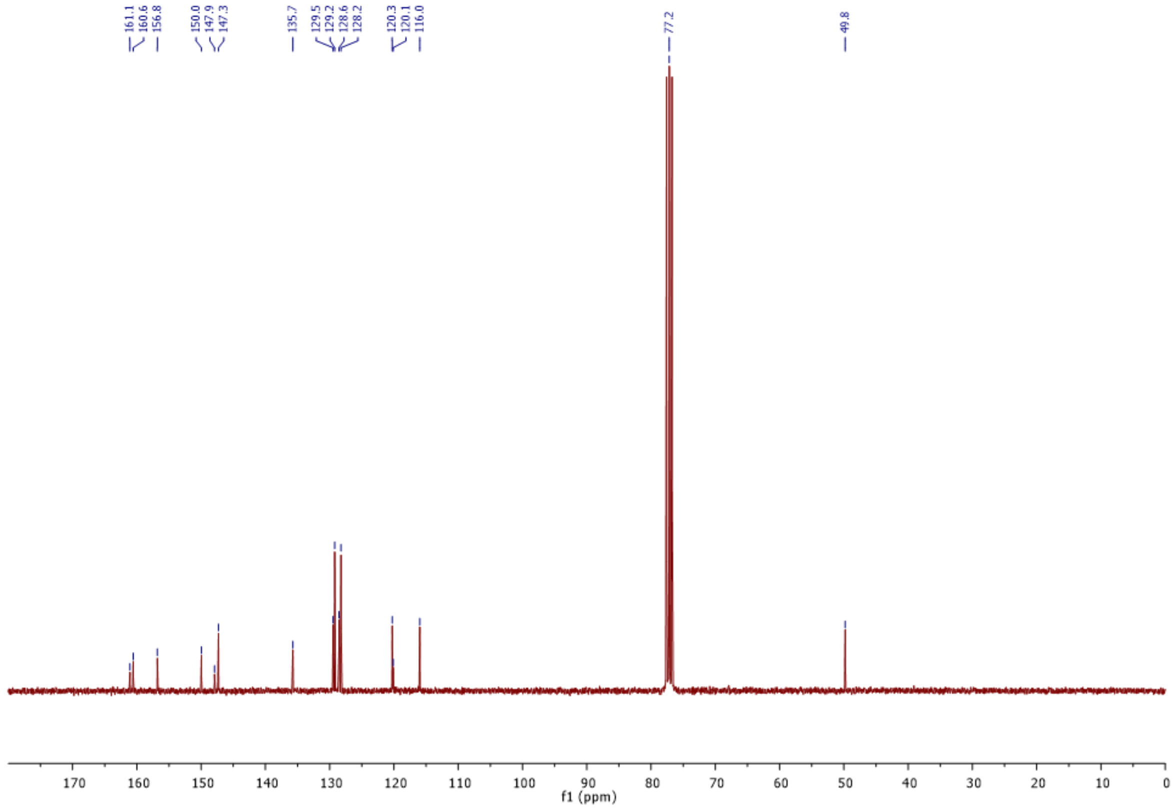

*3-Benzyl-8-[(4-chloro-5H-1,2,3-dithiazol-5-ylidene)amino]quinazolin-4(3H)-one (**3c**).*

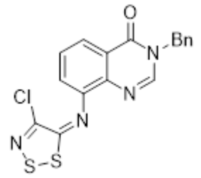


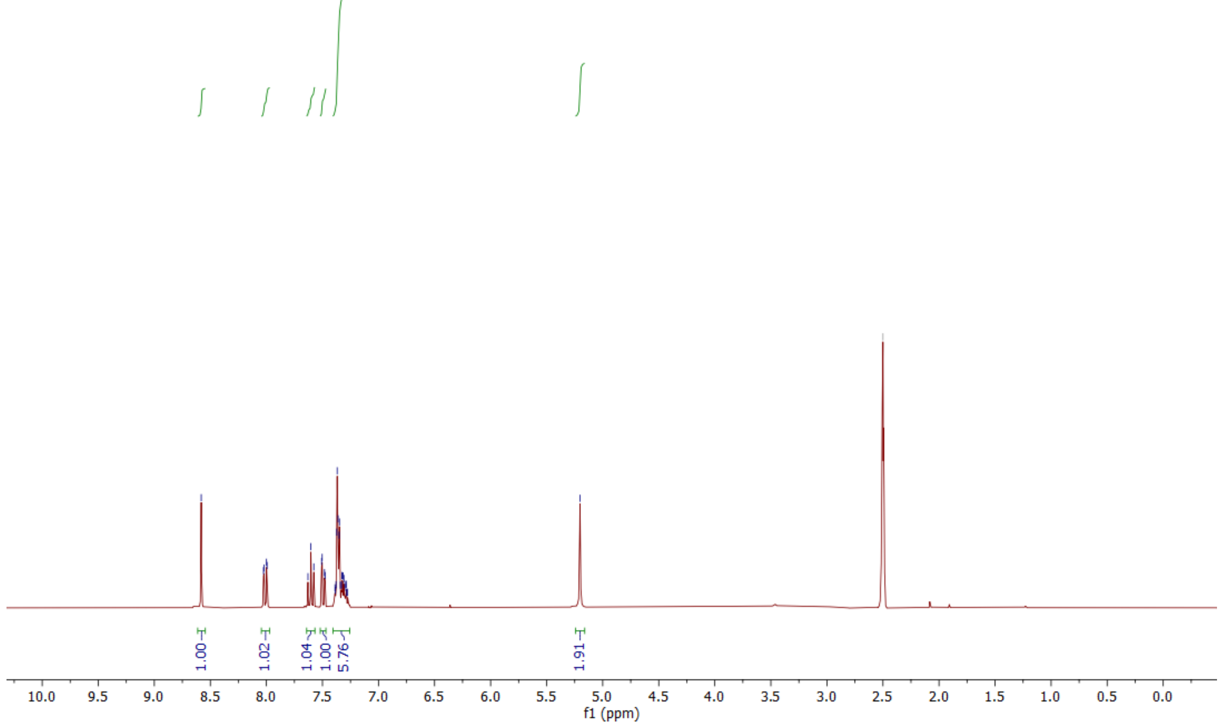


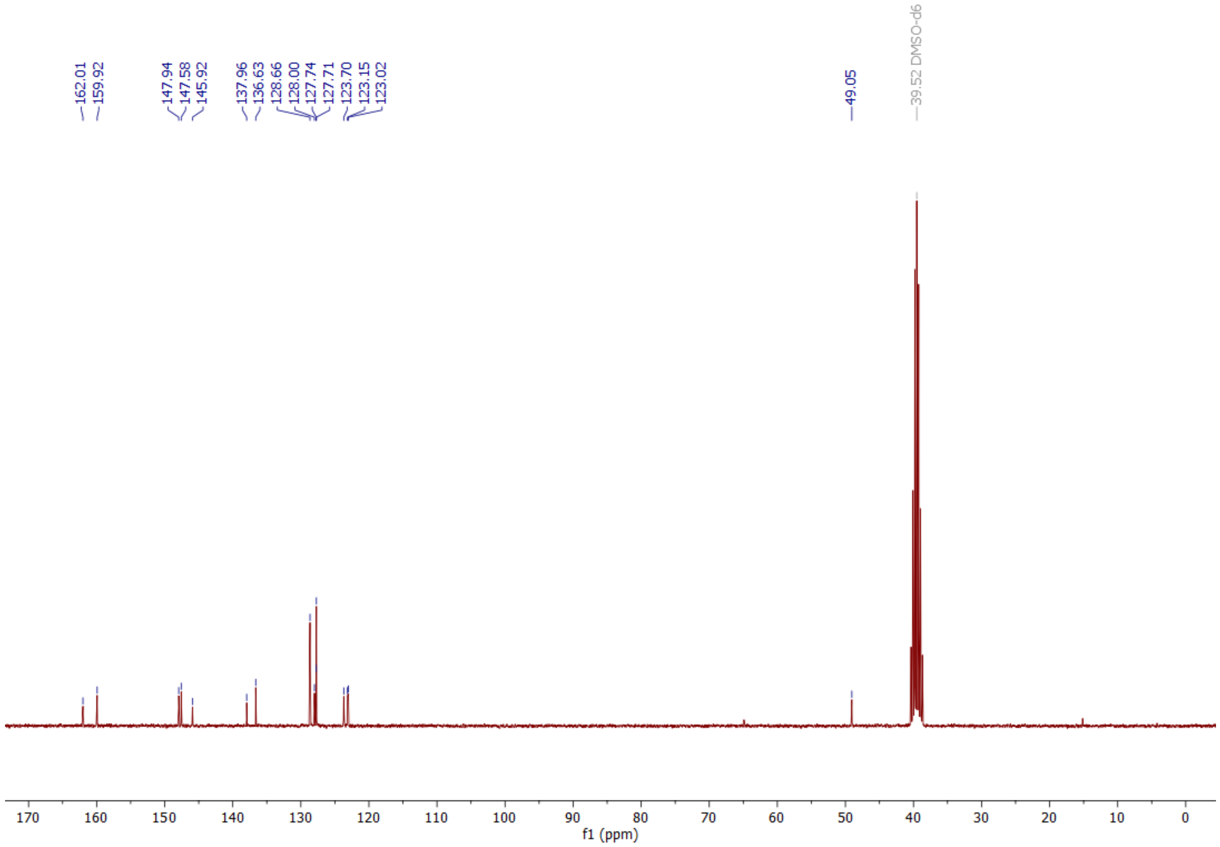

*(3-Cyclopropyl-4-oxo-3,4-dihydroquinazolin-6-yl)carbamothioyl cyanide (**4a**).*

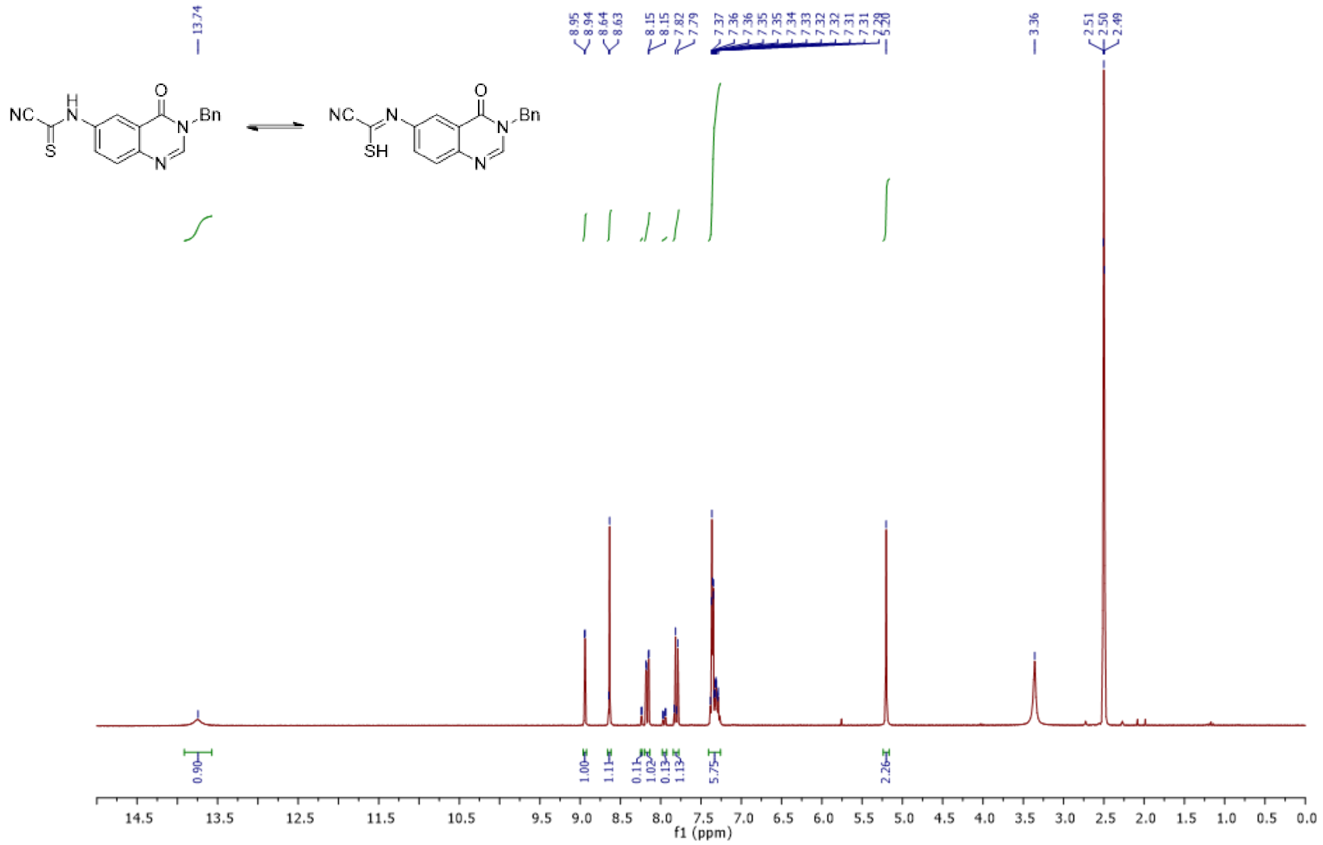


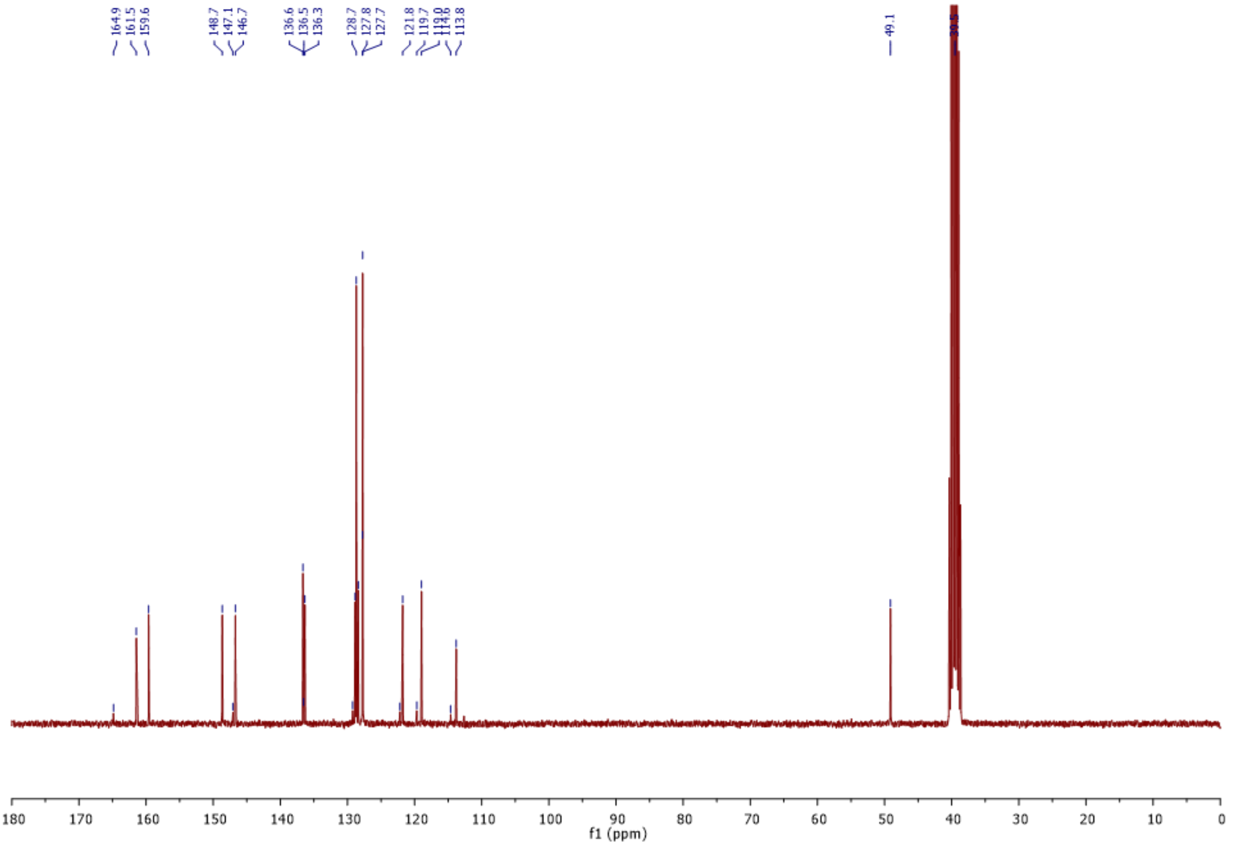

*(3-Benzyl-4-oxo-3,4-dihydroquinazolin-7-yl)carbamothioyl cyanide (**4b**).*

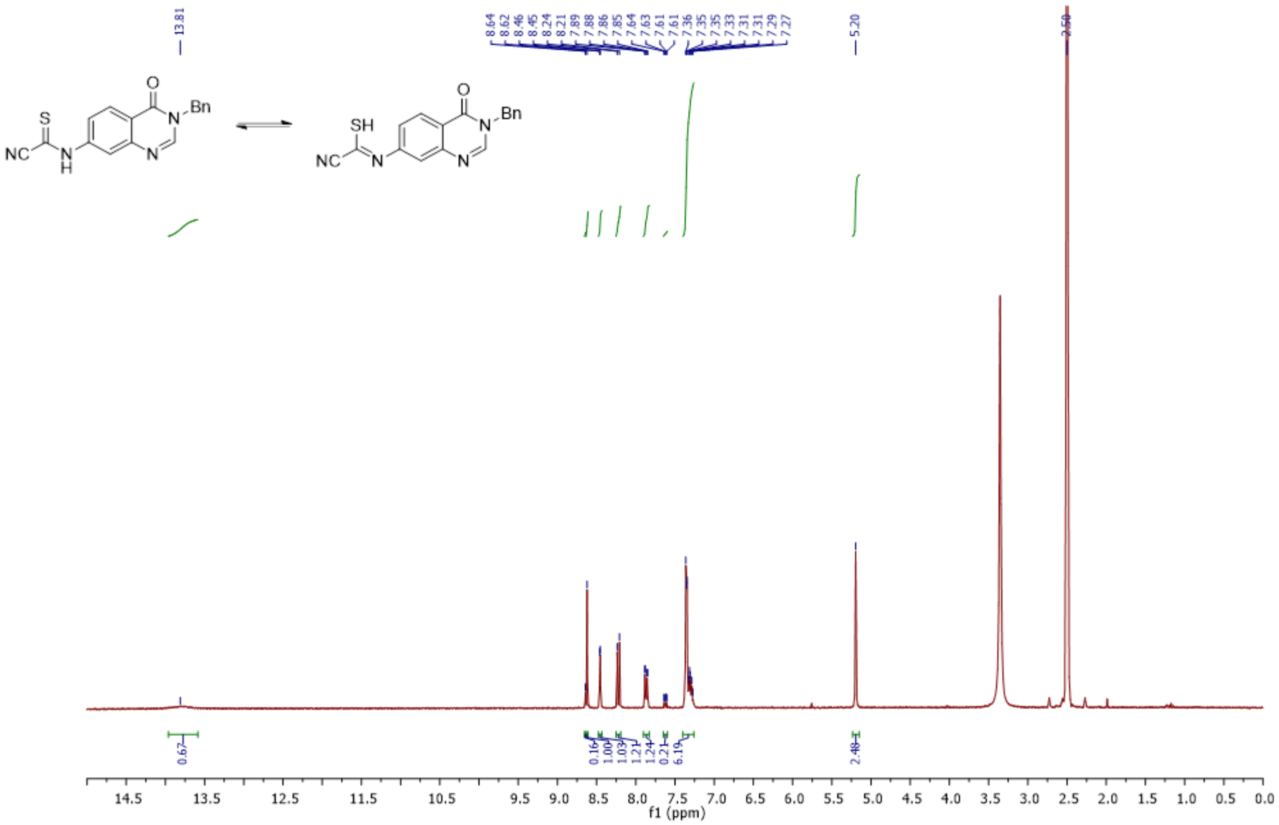


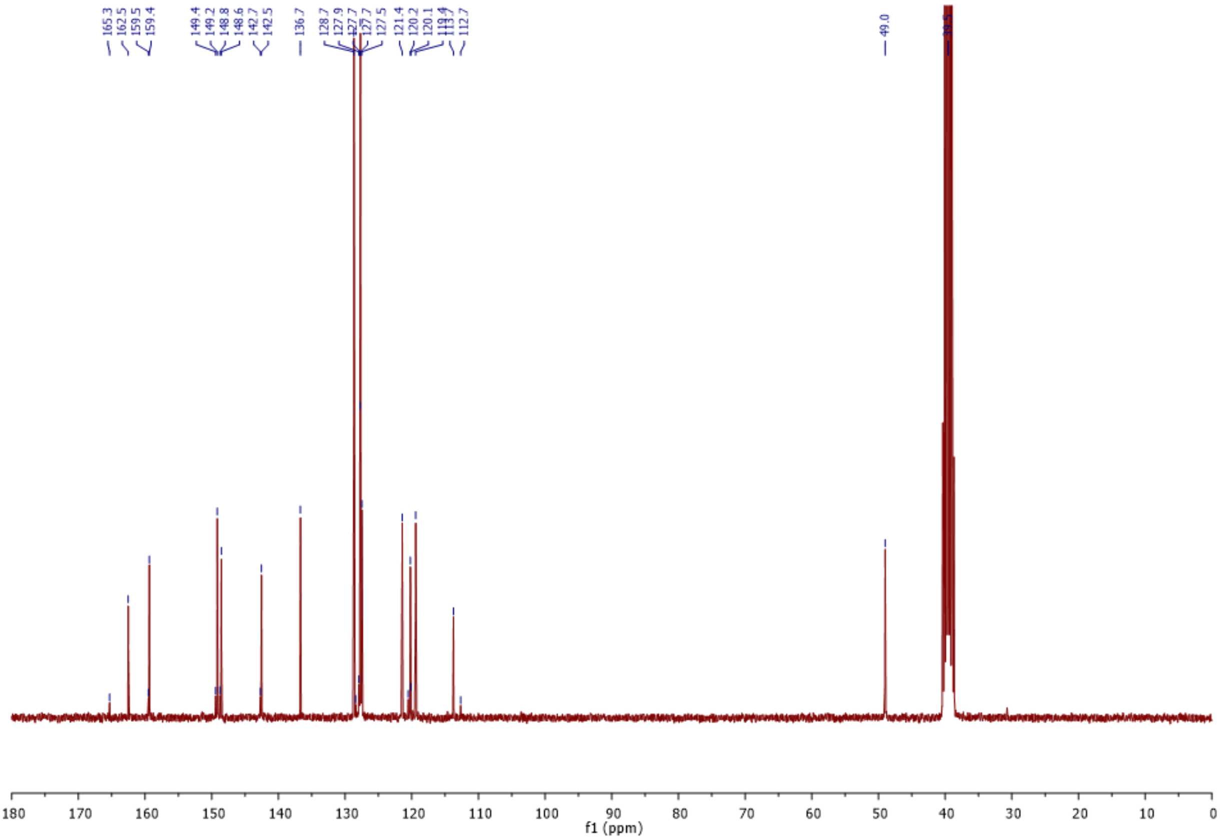

*(3-Benzyl-4-oxo-3,4-dihydroquinazolin-8-yl)carbamothioyl cyanide (**4c**).*

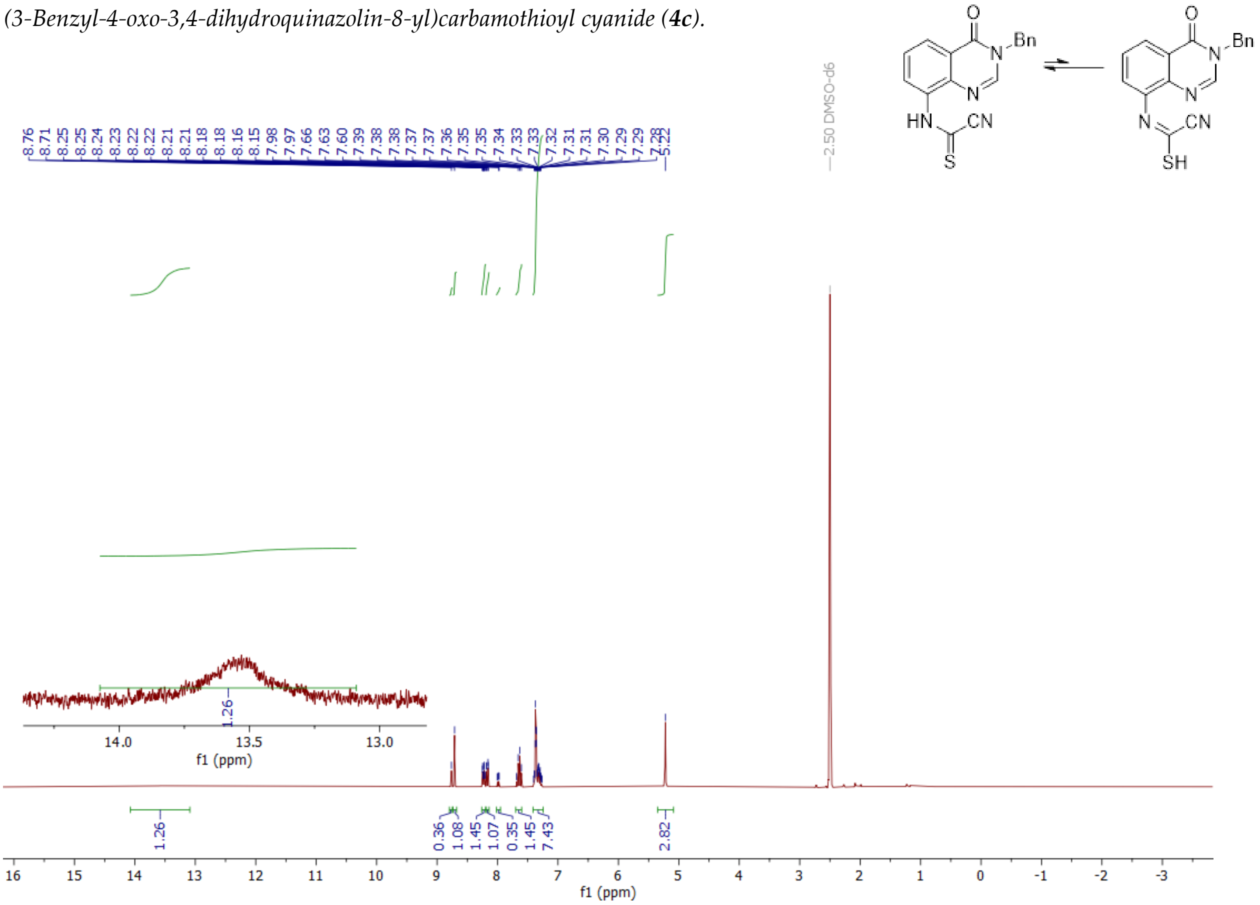


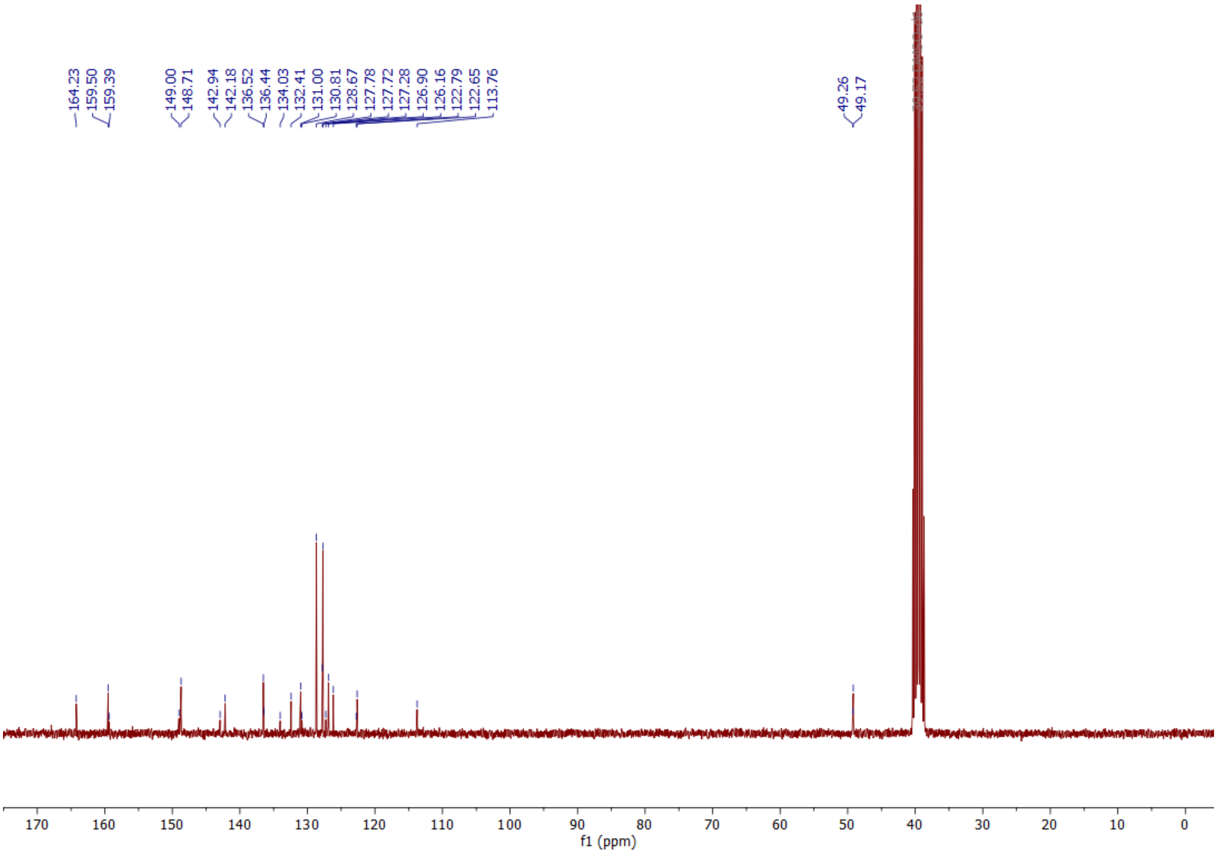

*7-Benzyl-8-oxo-7,8-dihydrothiazolo[4,5-g]quinazoline-2-carbonitrile (**5a**).*

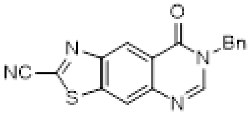


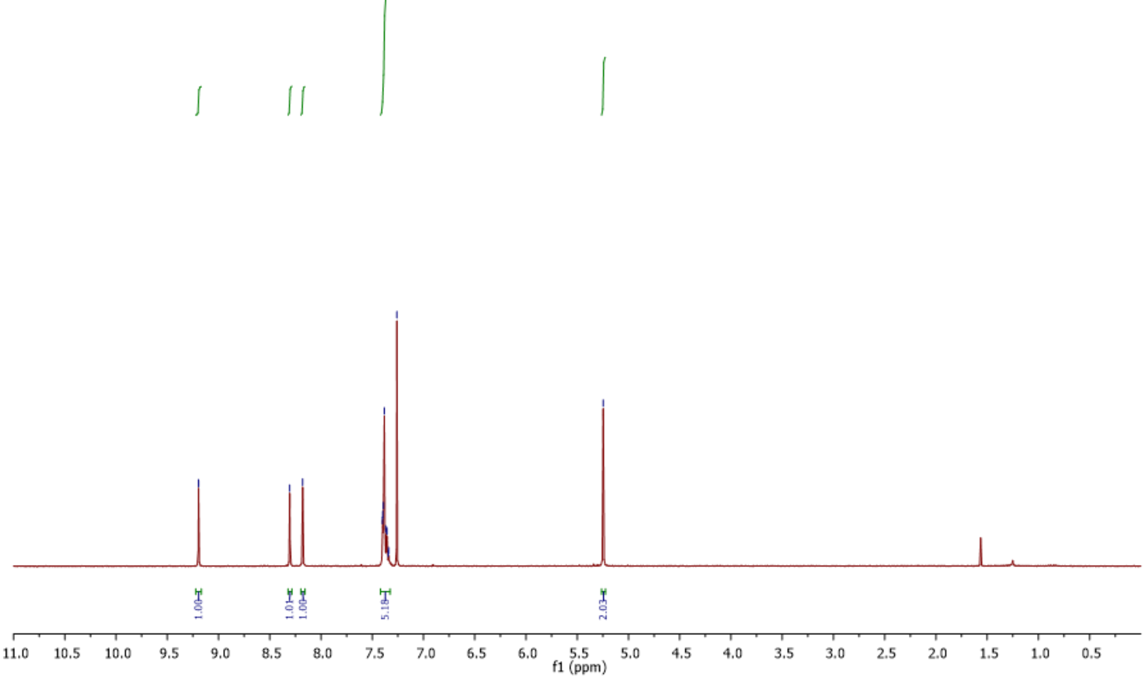


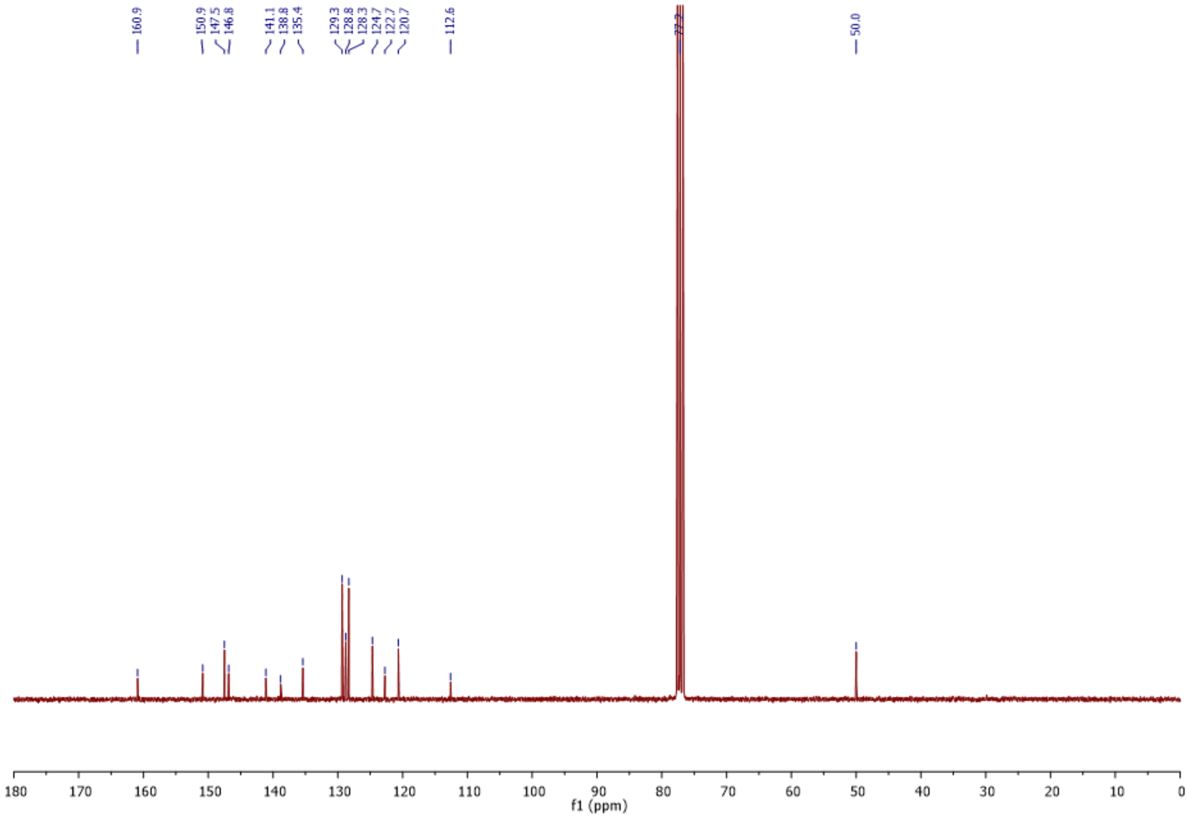

*7-Benzyl-8-oxo-7,8-dihydrothiazolo[5,4-g]quinazoline-2-carbonitrile (**5b**).*

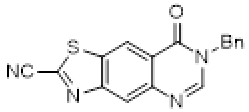


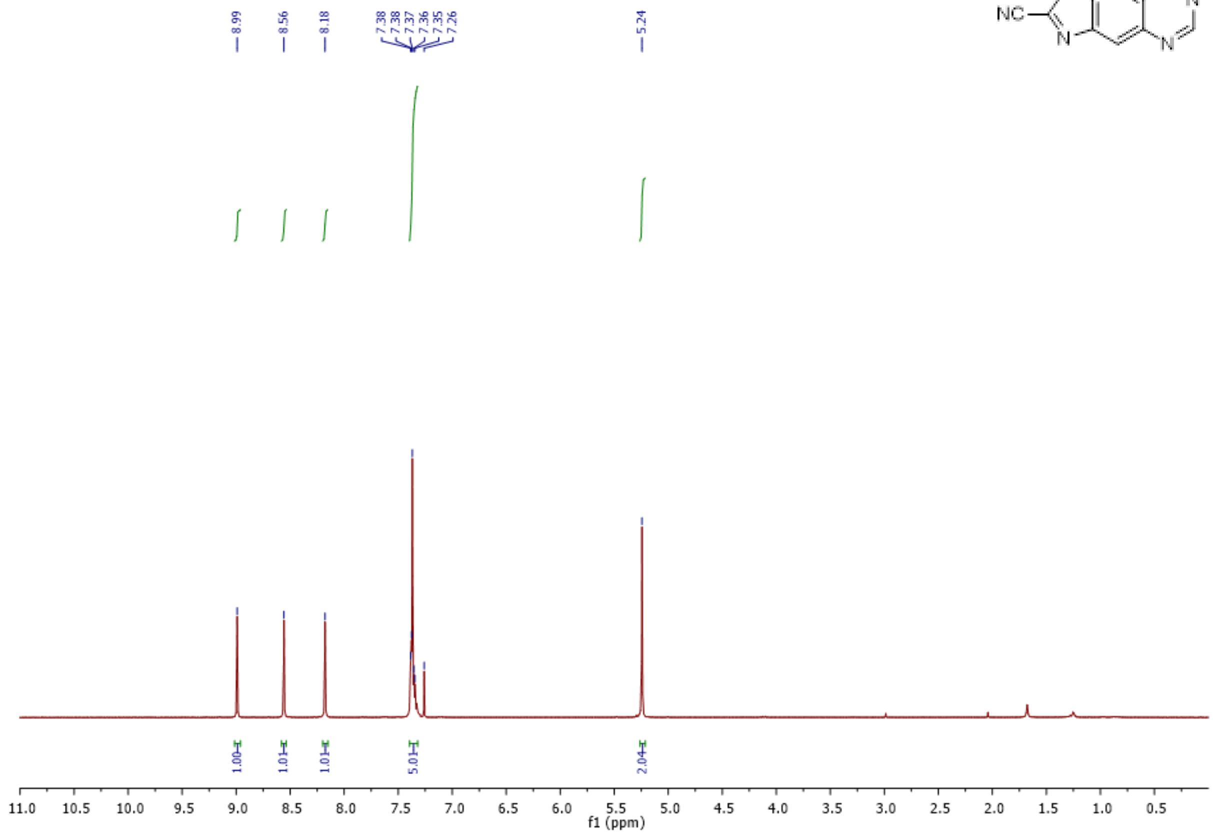


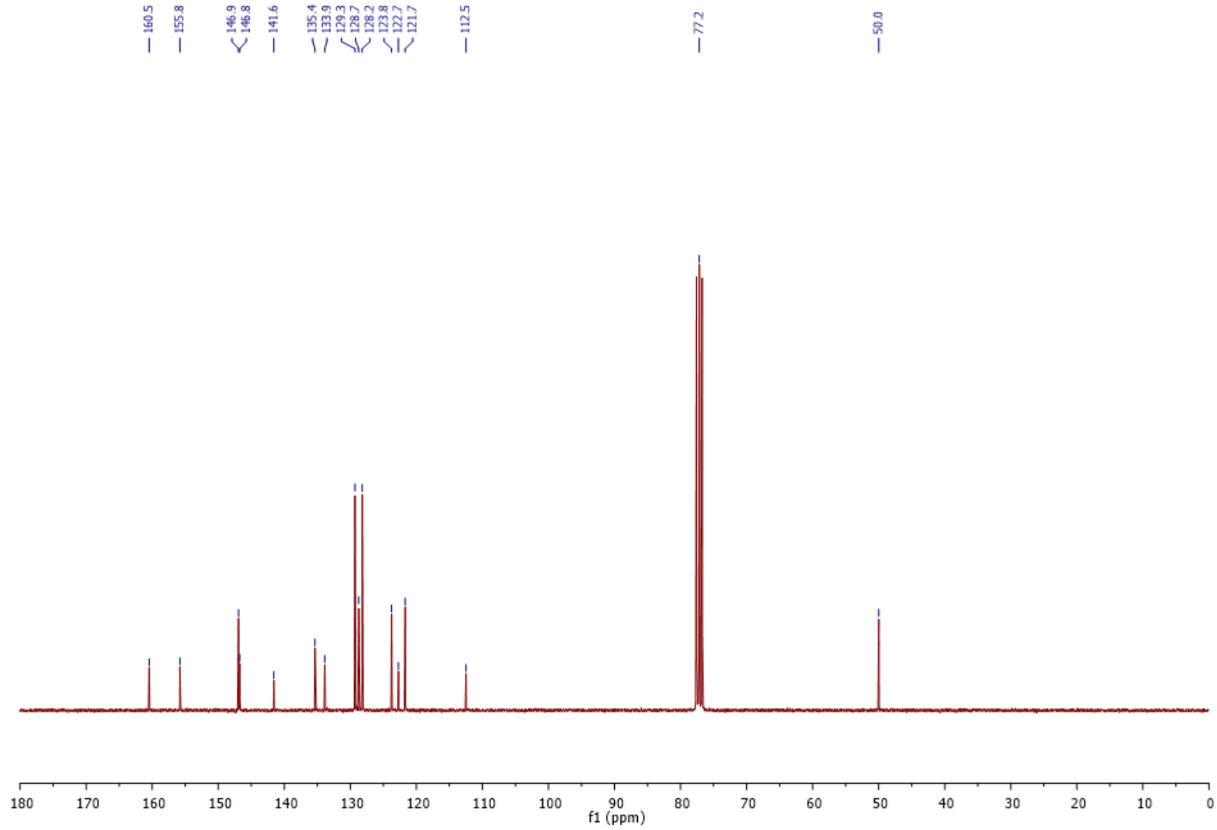

*7-Benzyl-6-oxo-6,7-dihydrothiazolo[5,4-h]quinazoline-2-carbonitrile* ***(5c).***

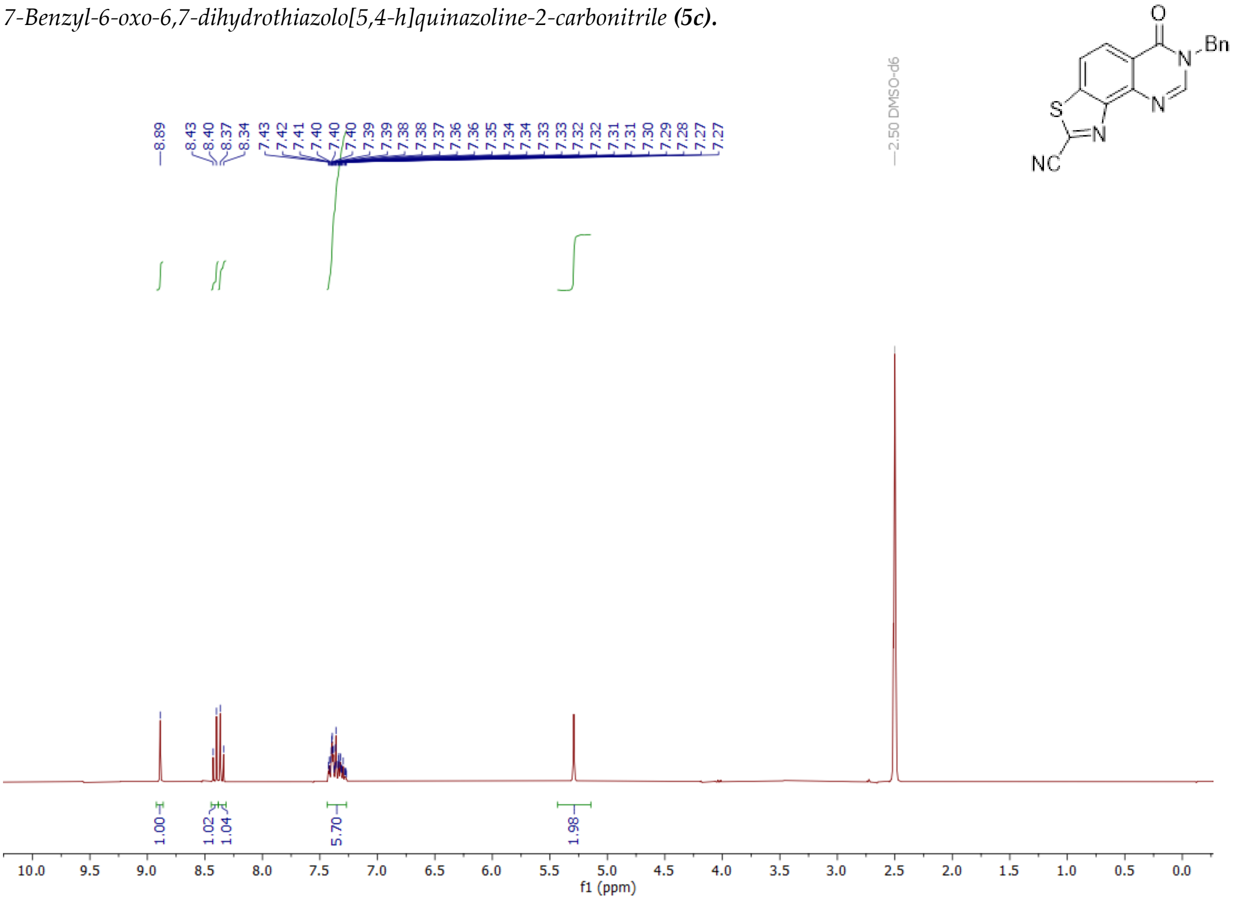


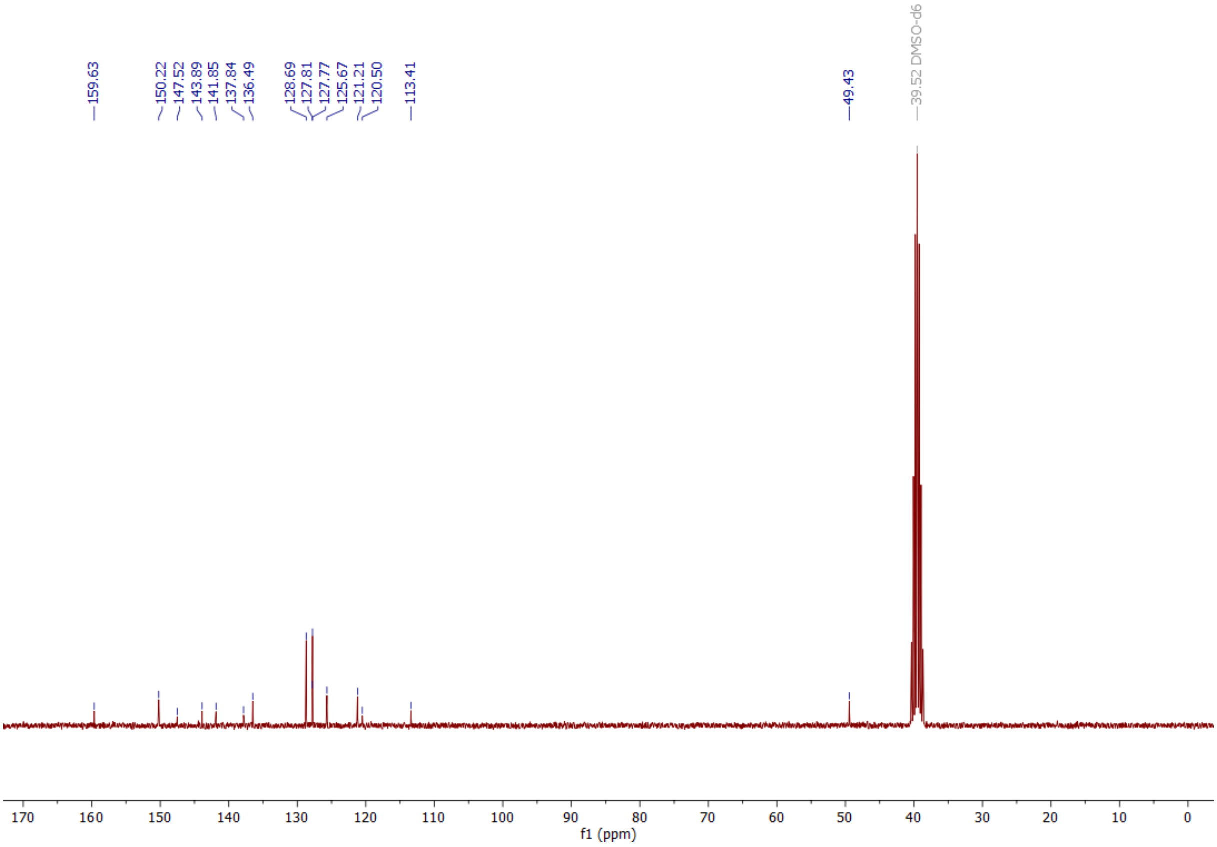

*Methyl 7-benzyl-8-oxo-7,8-dihydrothiazolo[4,5-g]quinazoline-2-carbimidate (**6a**).*

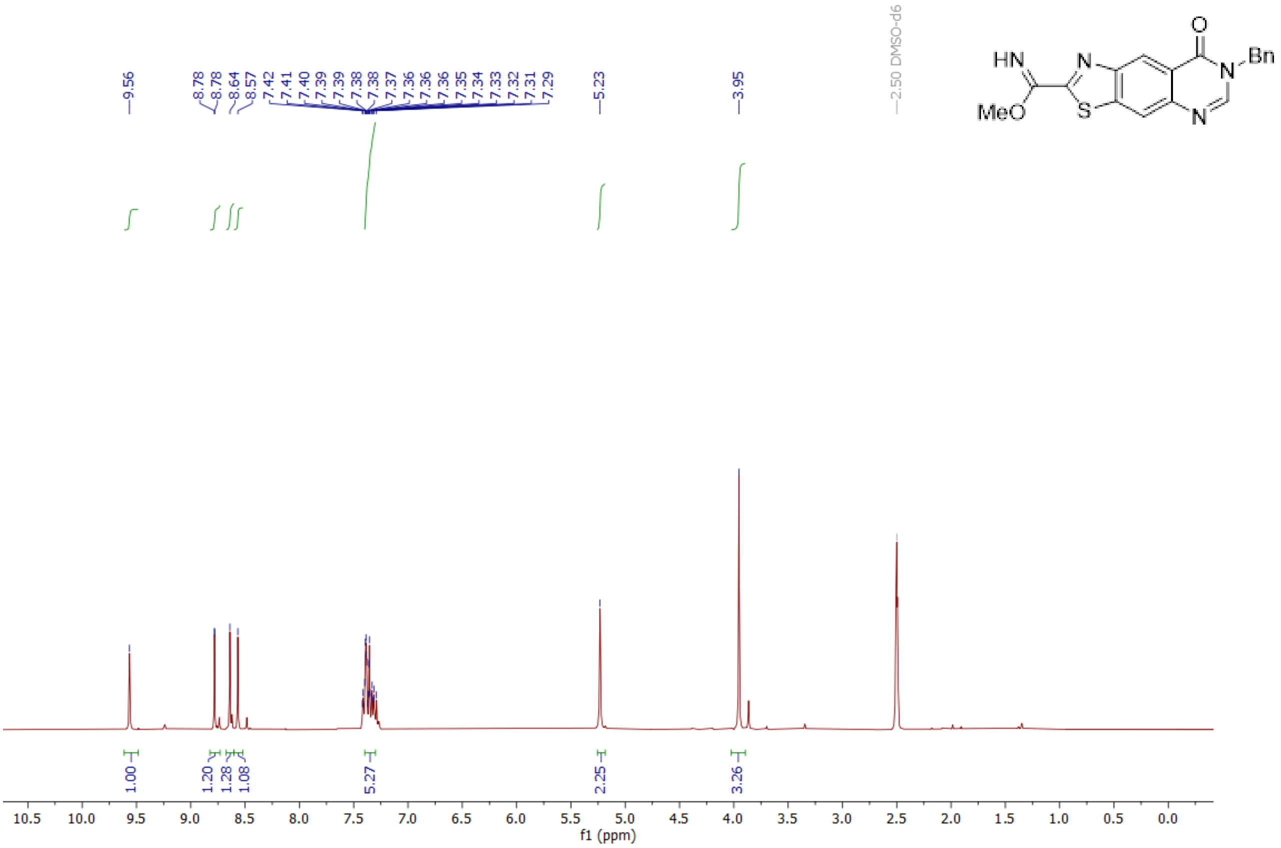


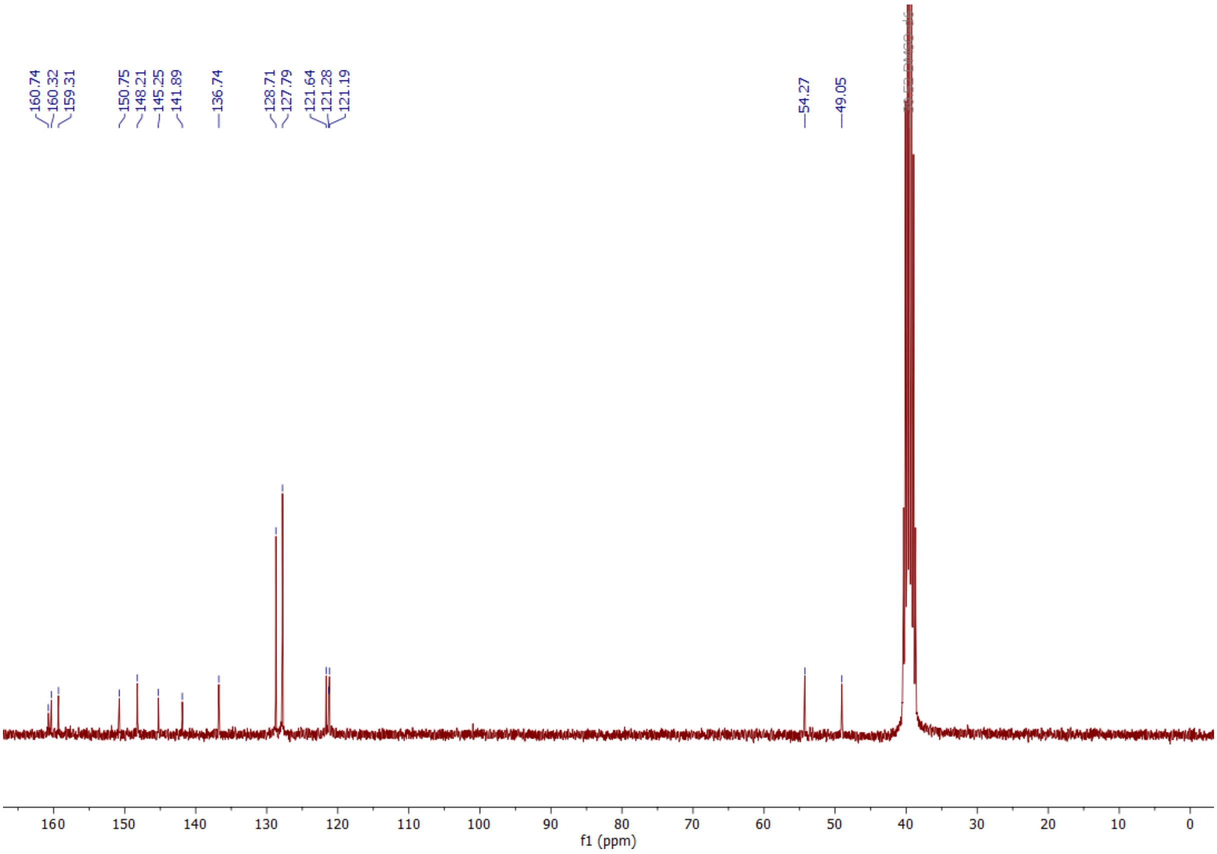

*Methyl 7-benzyl-8-oxo-7,8-dihydrothiazolo[5,4-g]quinazoline-2-carbimidate* ***(6b).***

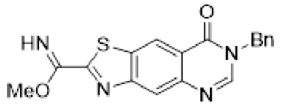


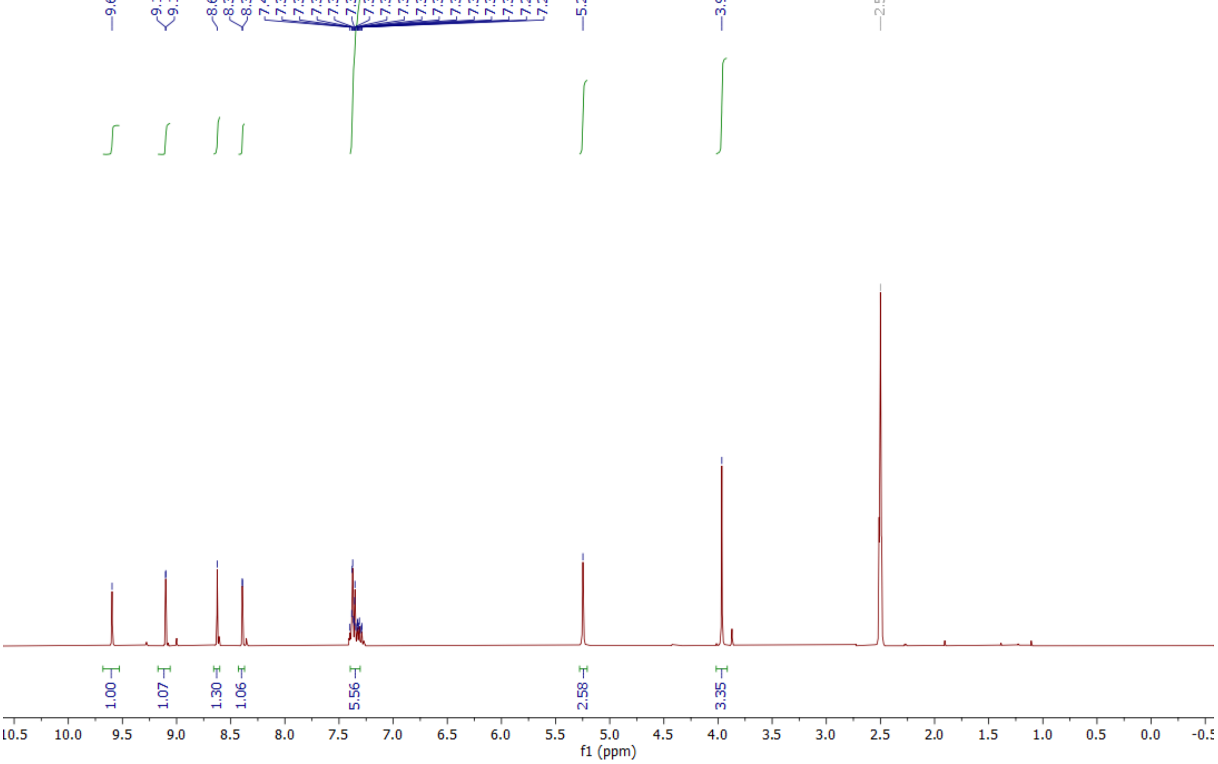


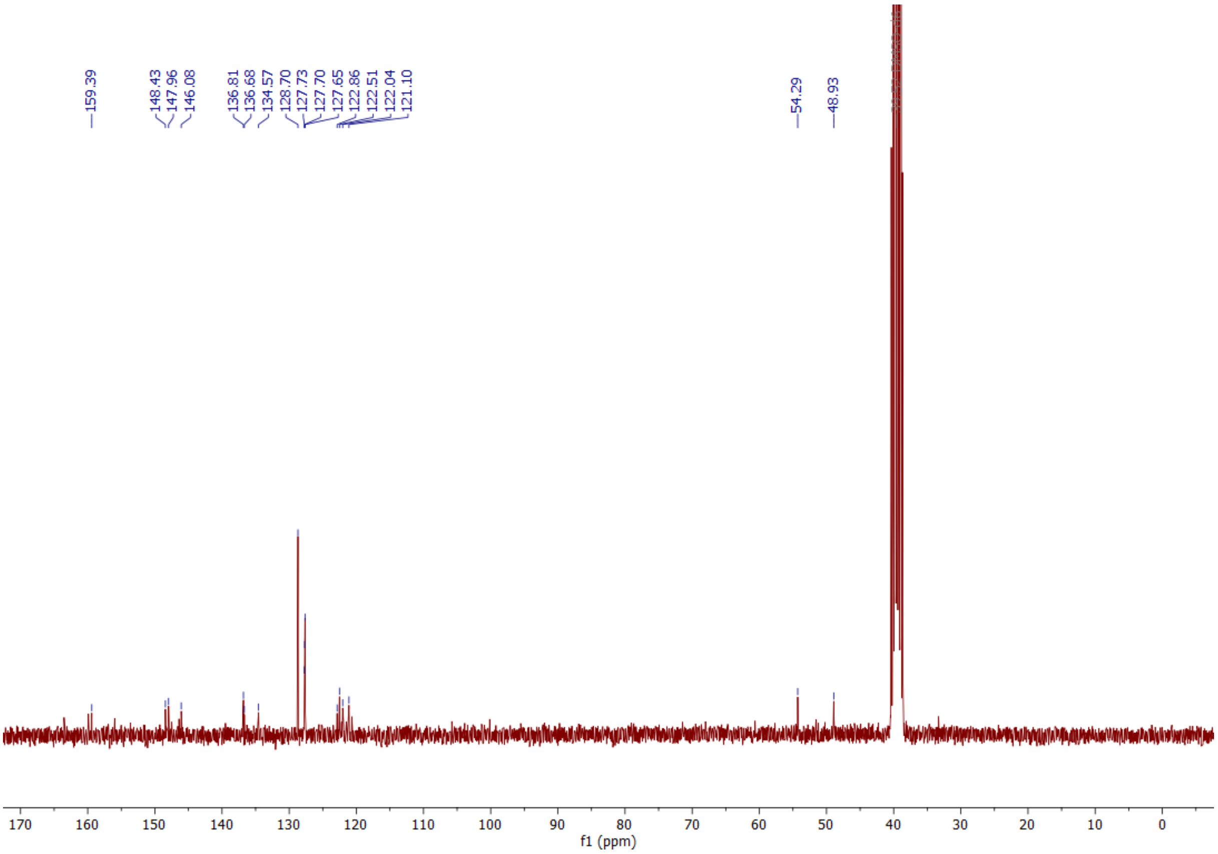

*Methyl 7-benzyl-6-oxo-6,7-dihydrothiazolo[5,4-h]quinazoline-2-carbimidate* ***(6c).***

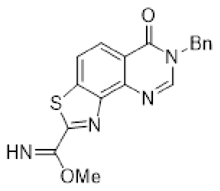


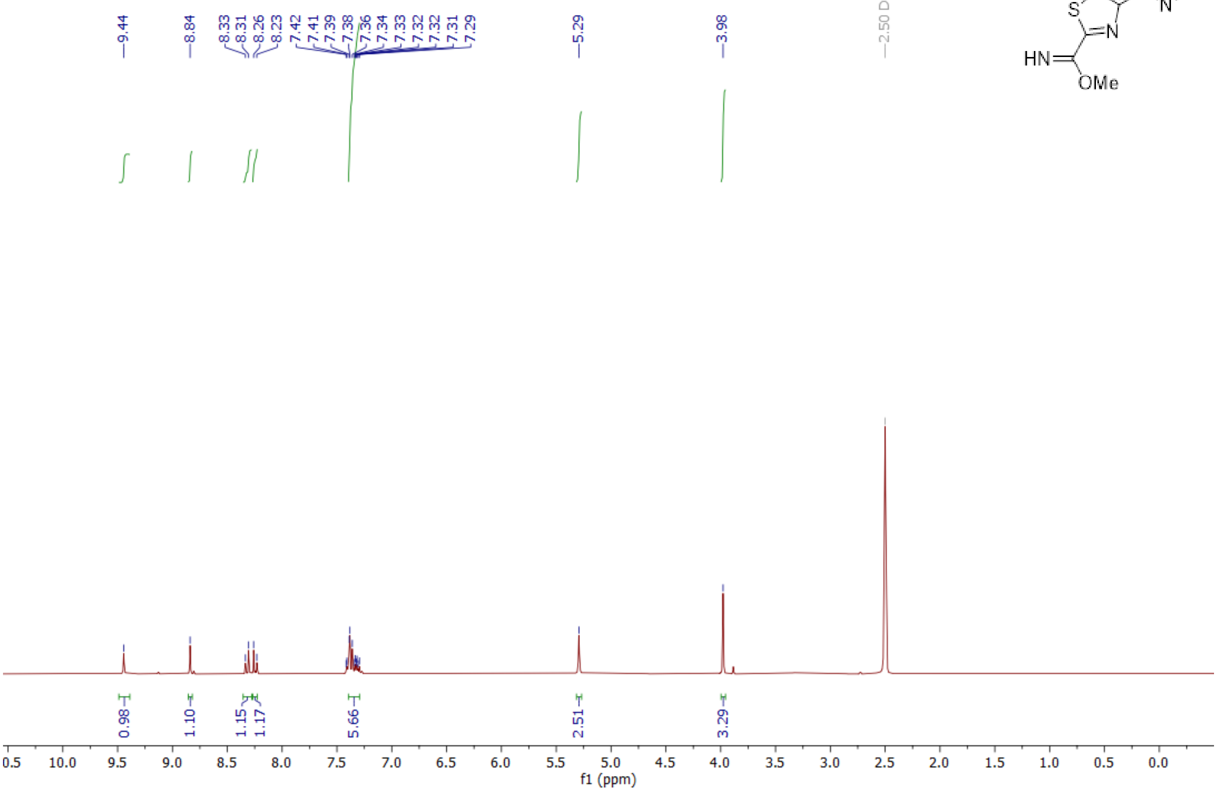


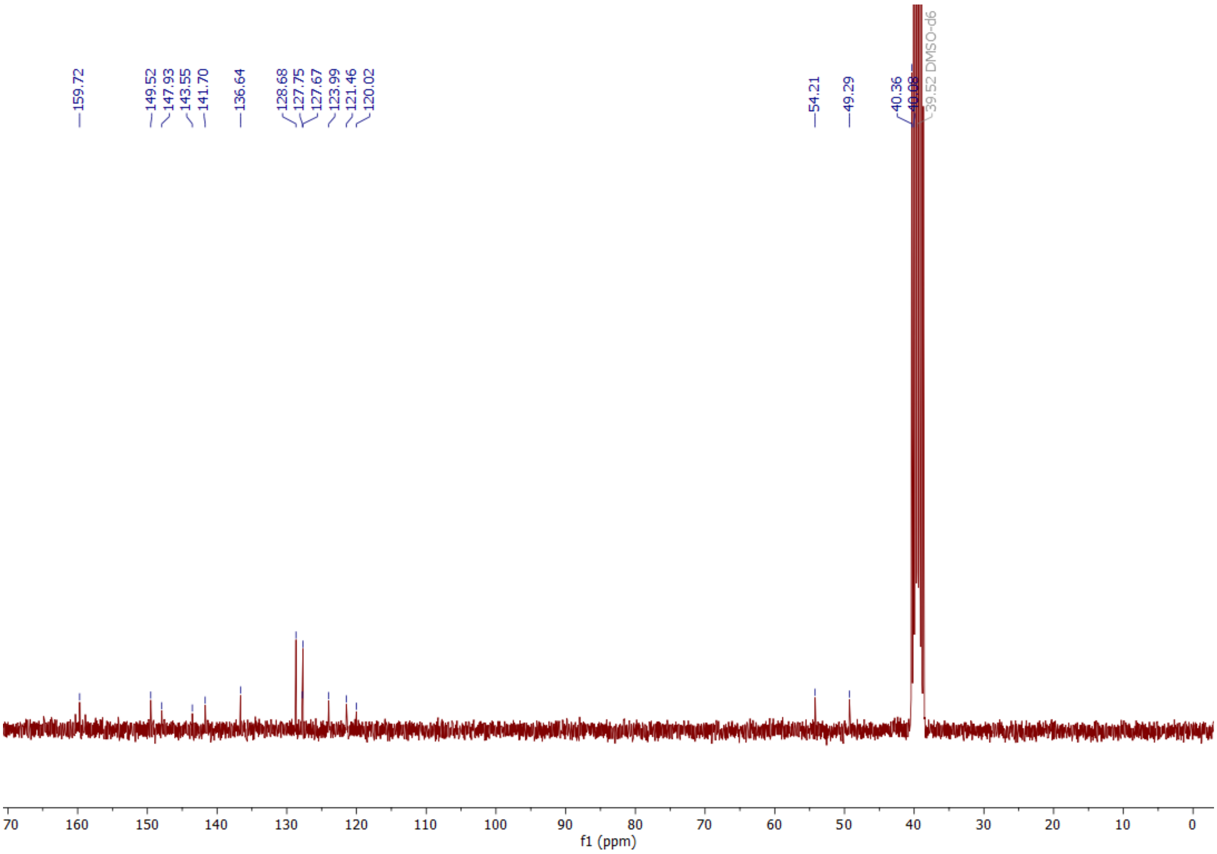

*Methyl 7-benzyl-6-oxo-6,7-dihydrothiazolo[4,5-h]quinazoline-2-carbimidate* ***(6d).***

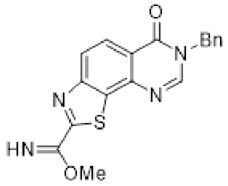


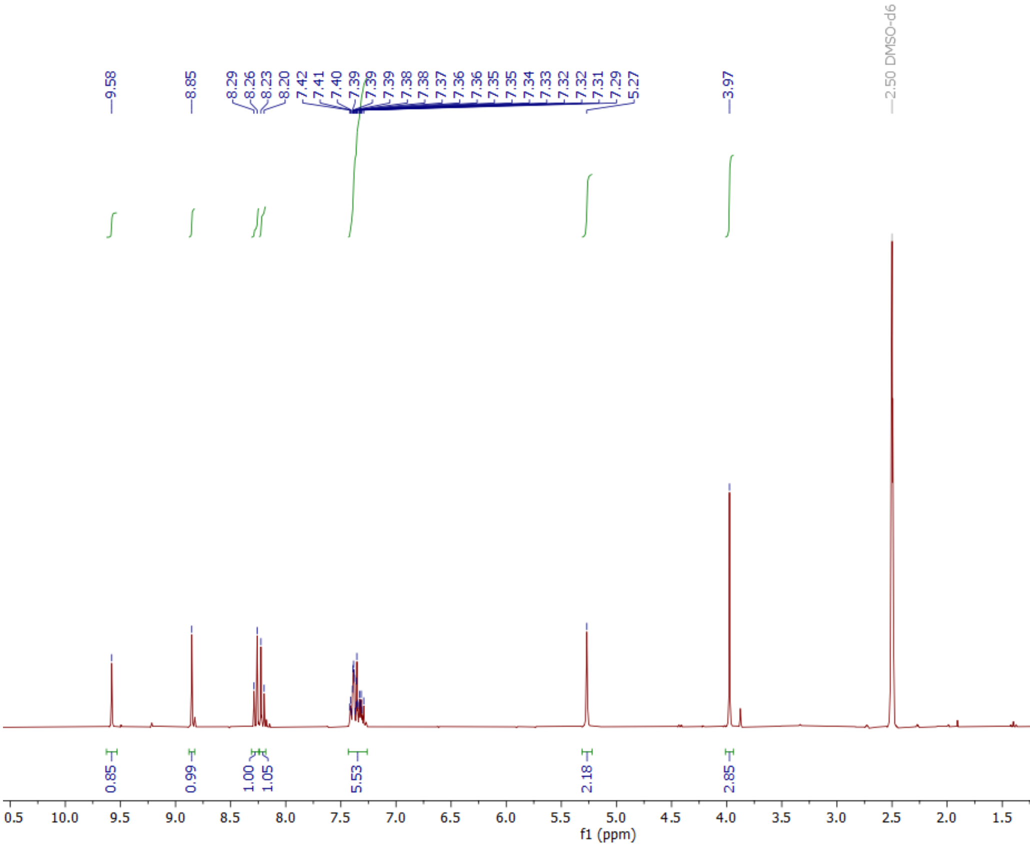


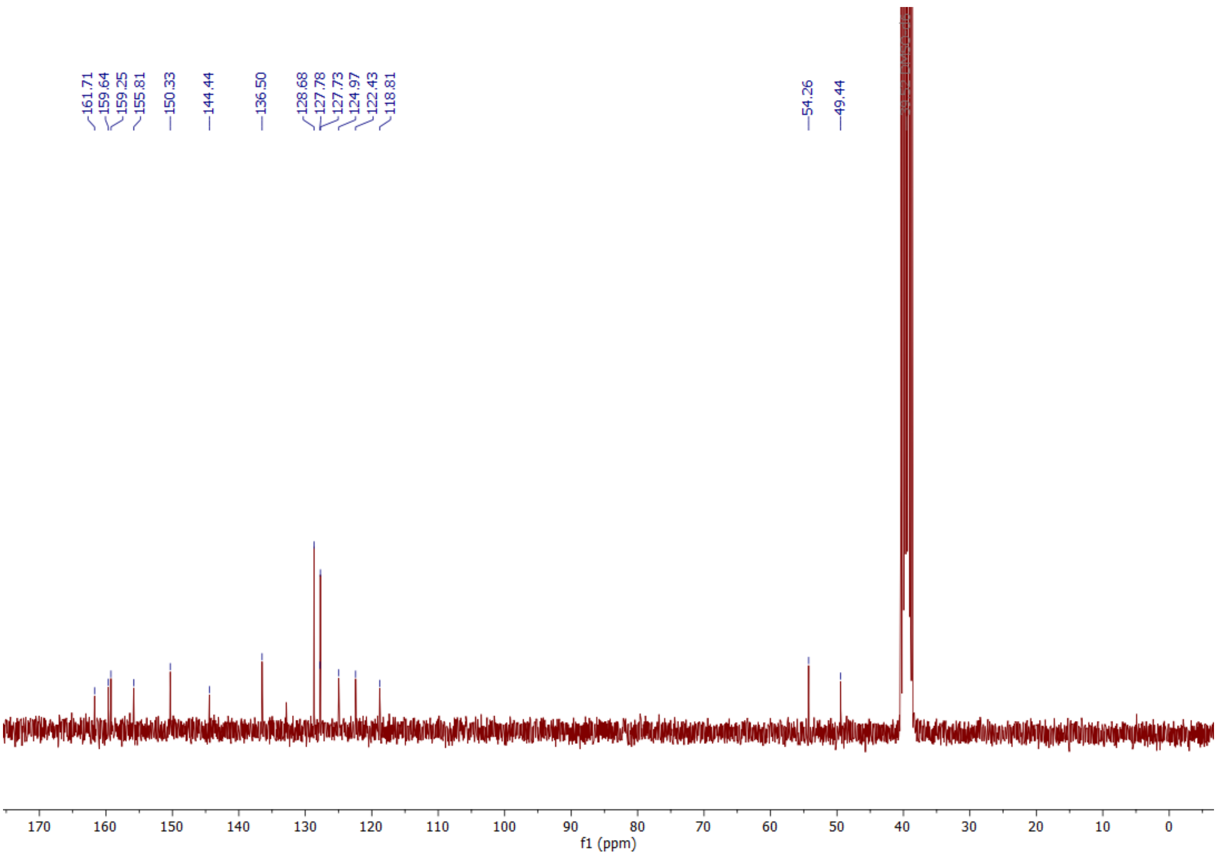

*8-Amino-3-benzyl-7-bromoquinazolin-4(3H)-one (**7a**).*

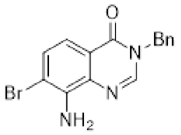


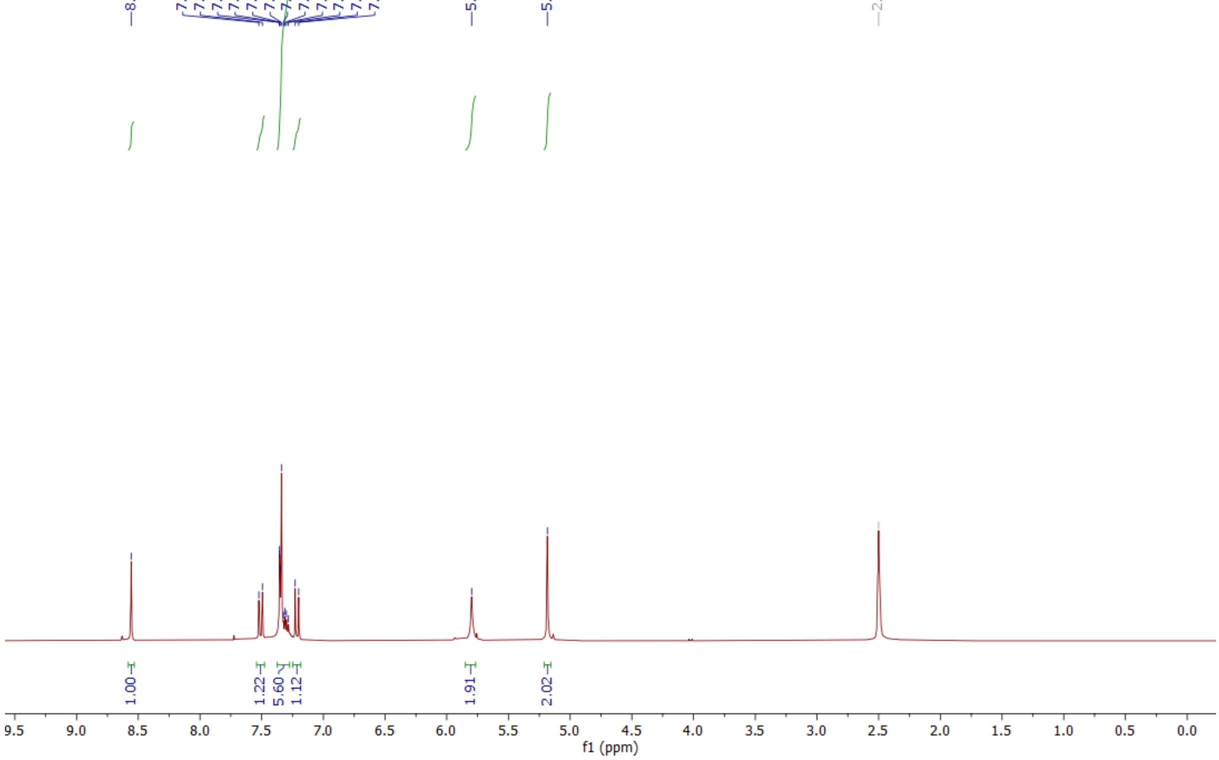


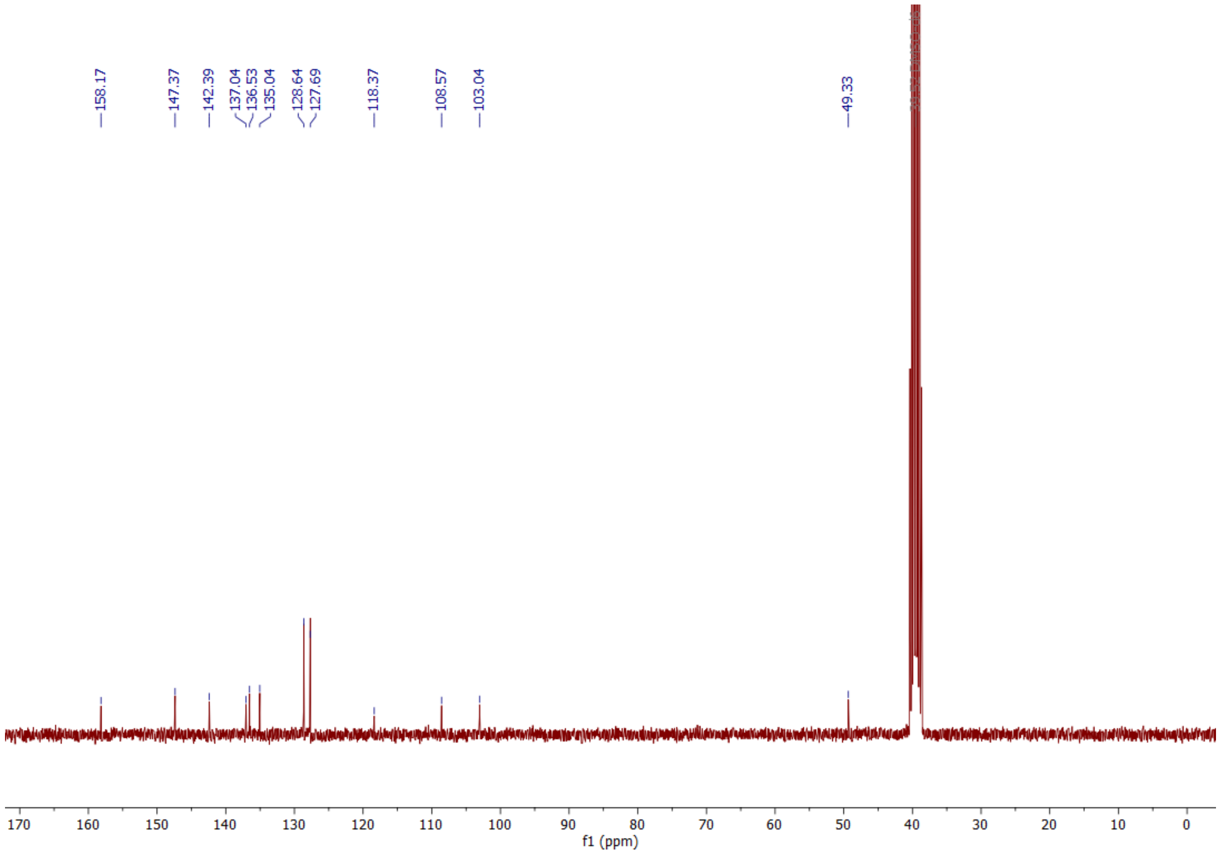

*3-Benzyl-7-bromo-8-[(4-chloro-5H-1,2,3-dithiazol-5-ylidene)amino]quinazolin-4(3H)-one(* ***7b).***

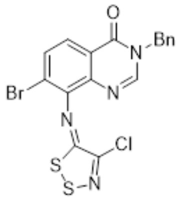


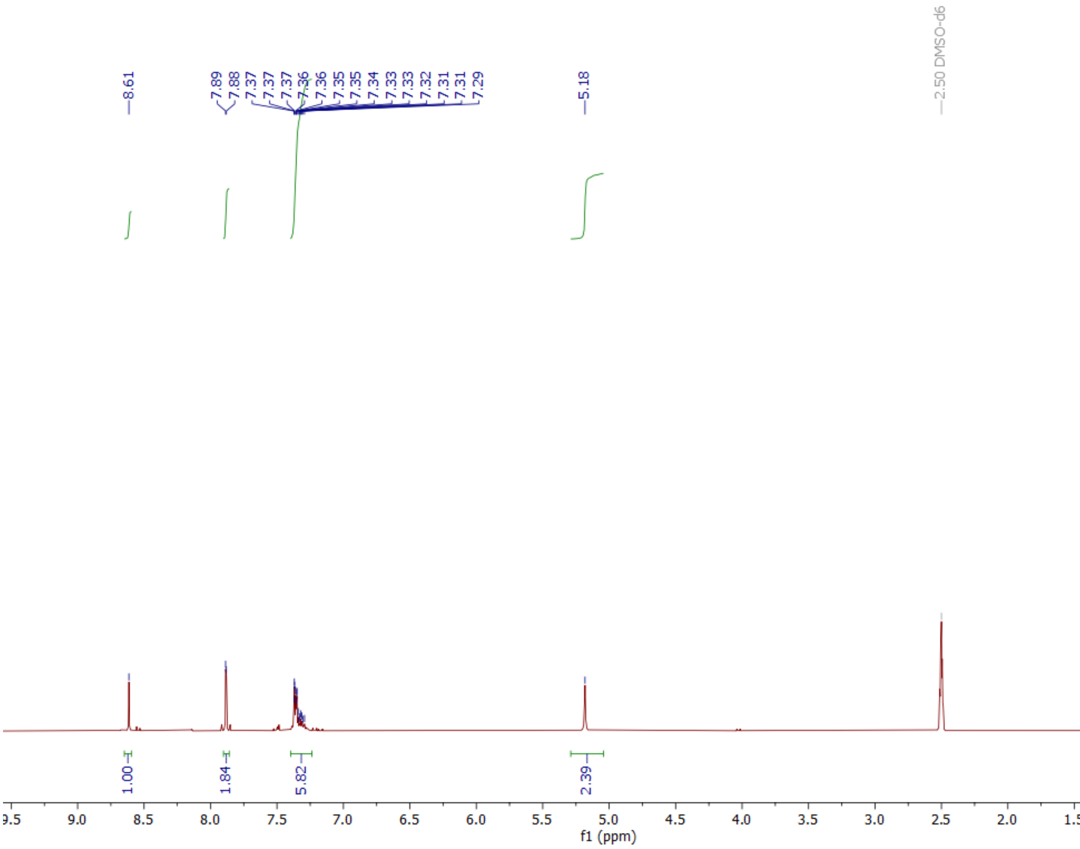


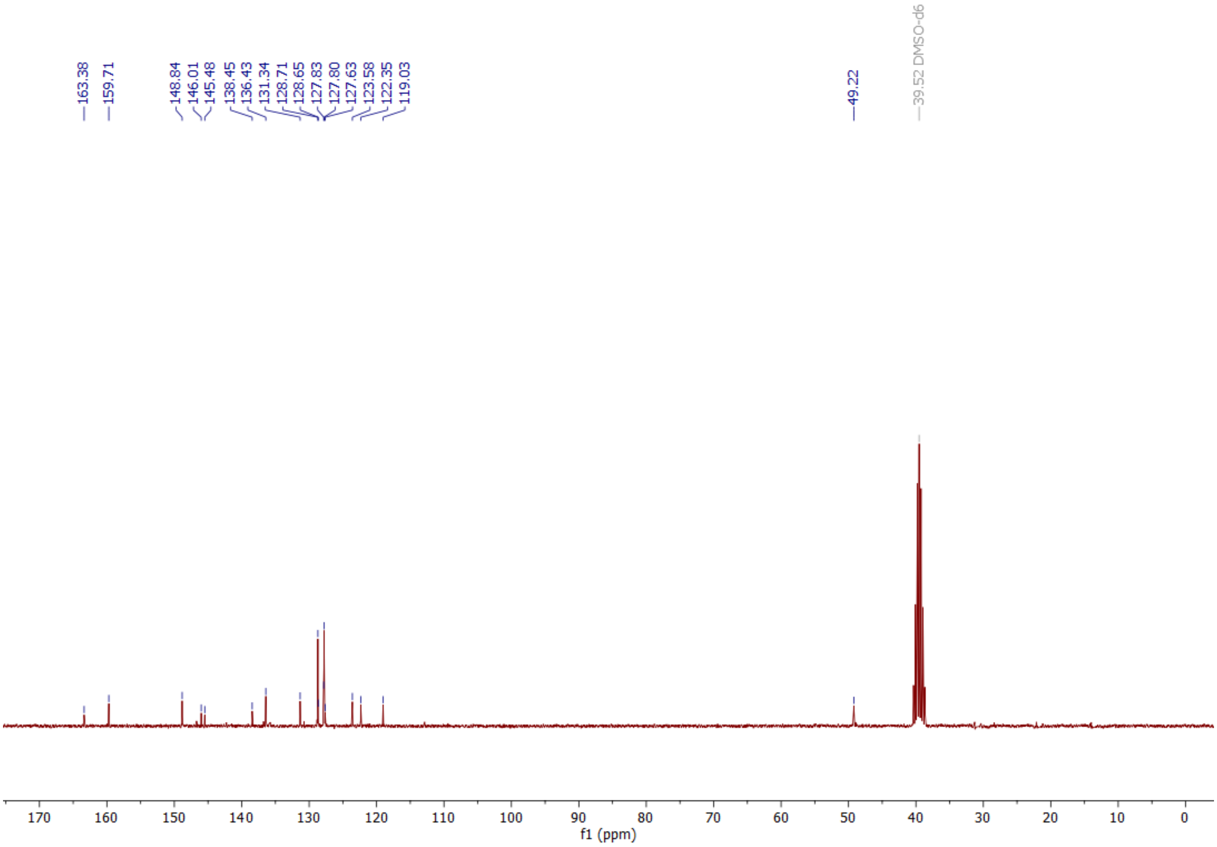

*(E)-N′-(2-cyano-5-nitrophenyl)-N,N-dimethylformimidamide (**9b**)*

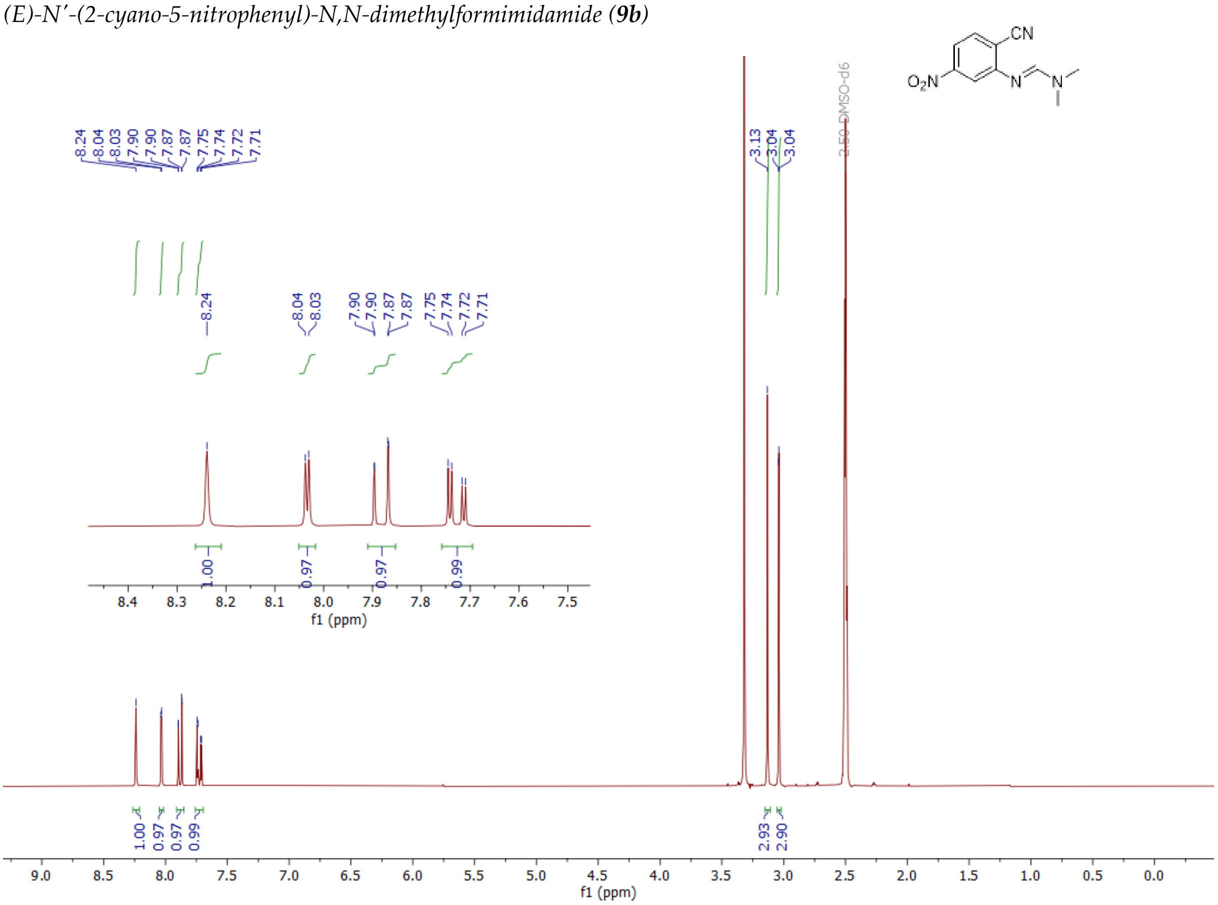


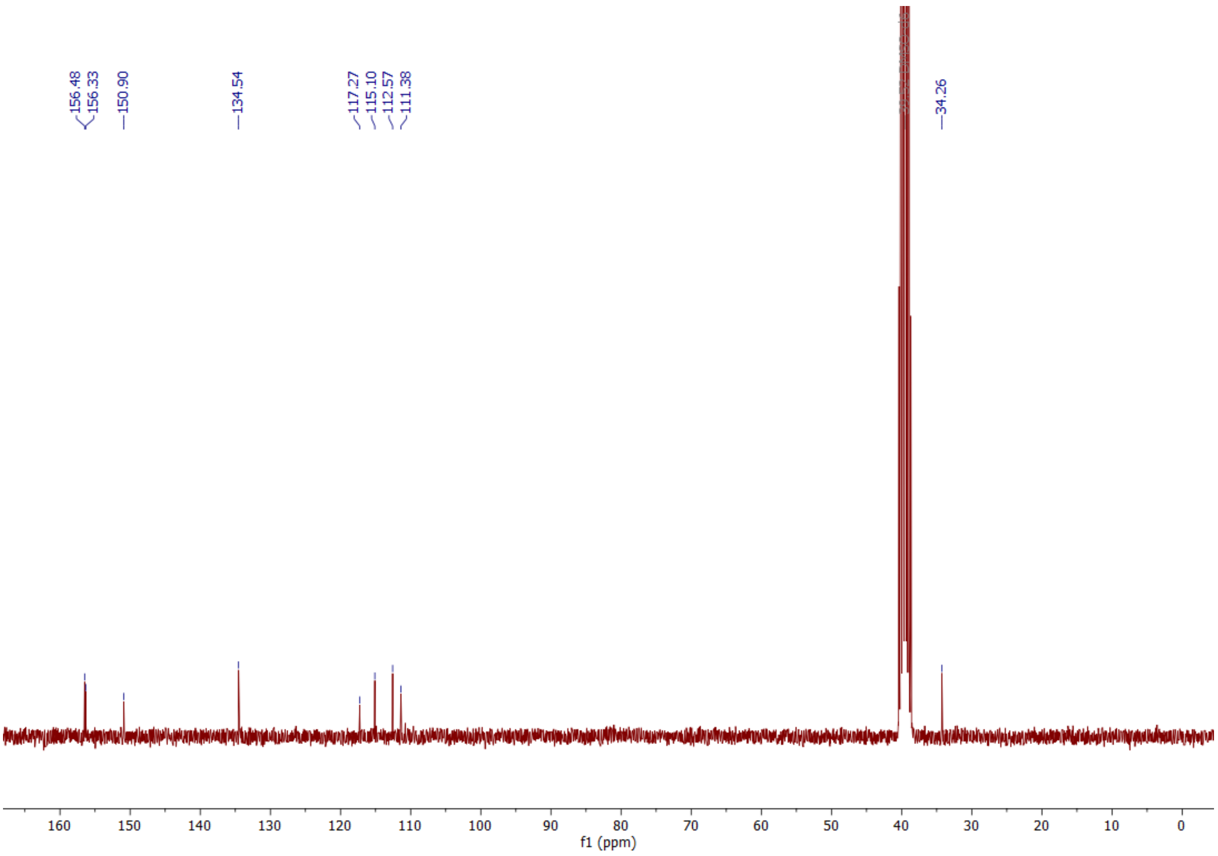

*N-(2-Fluoro-4-methoxyphenyl)-7-nitroquinazolin-4-amine* (***10b***).

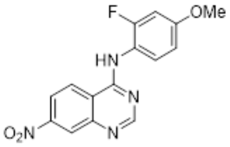


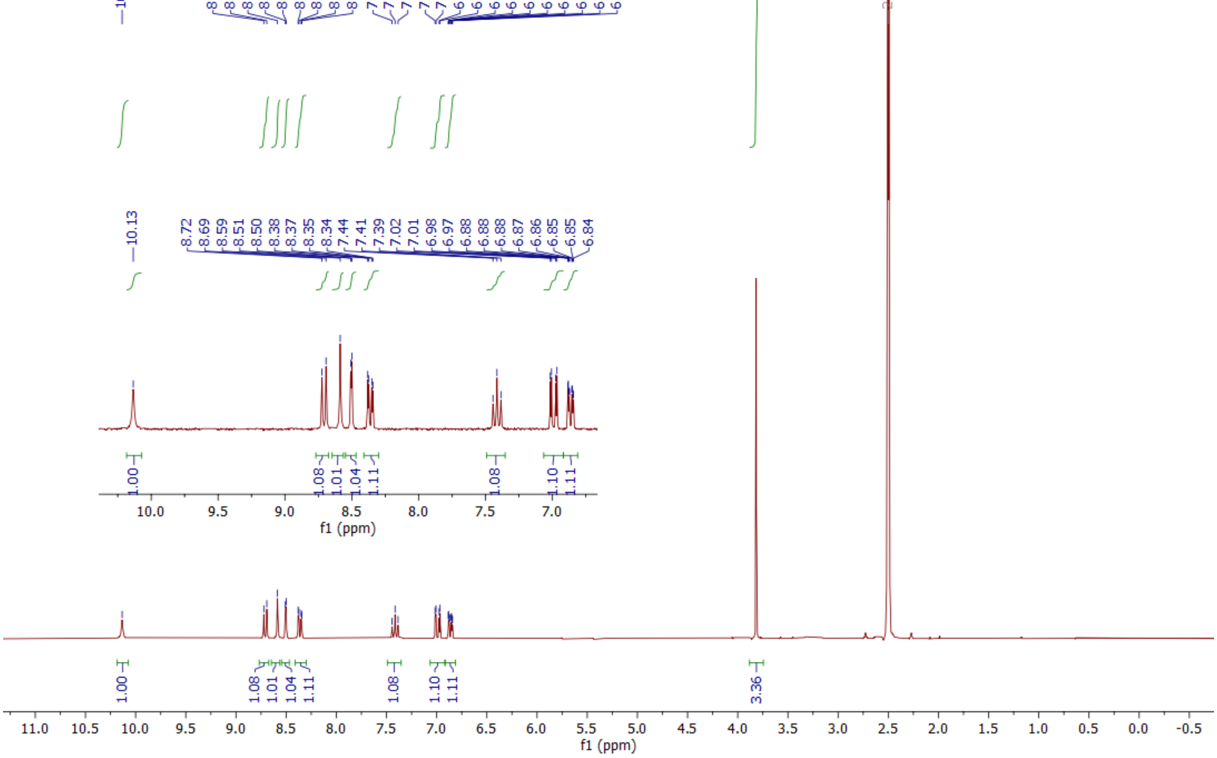


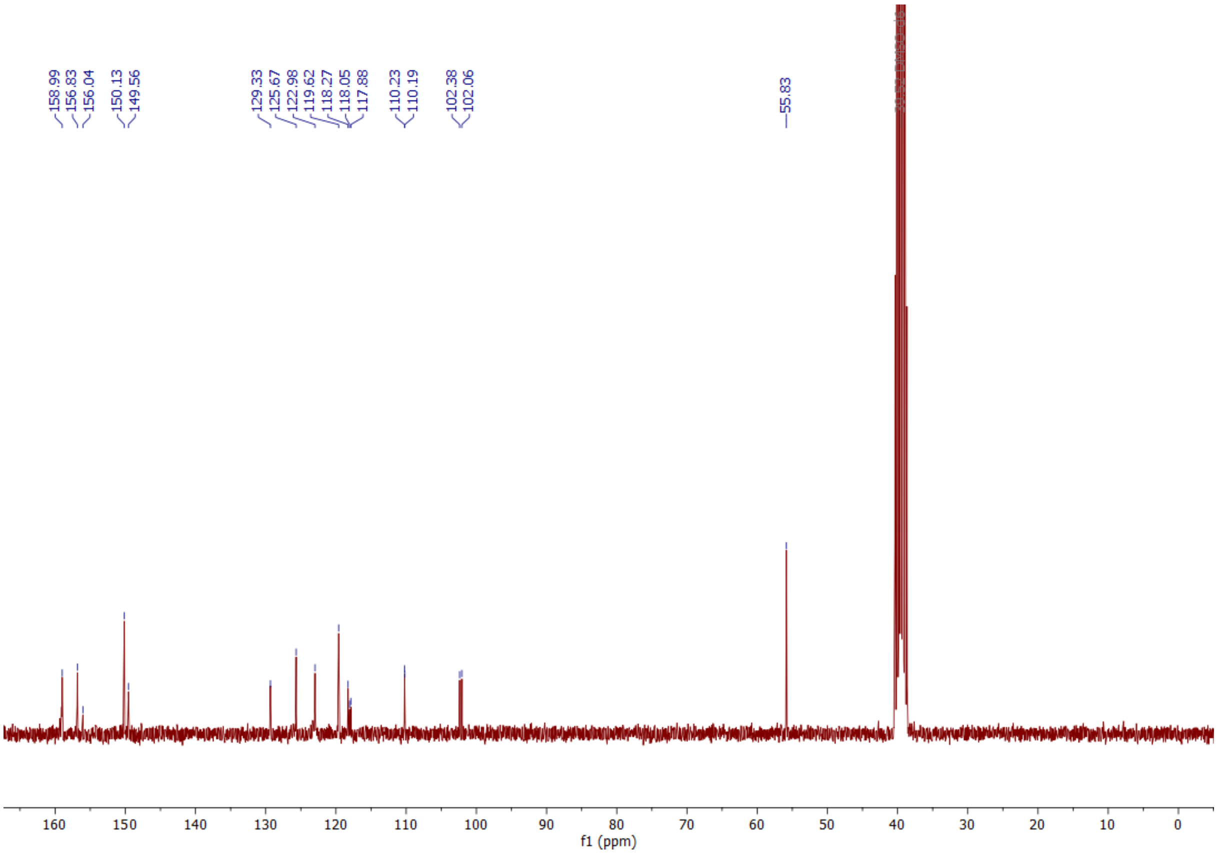

*N*[4]-(2-fluoro-4-methoxyphenyl)quinazoline-4,6-diamine (***11a***).

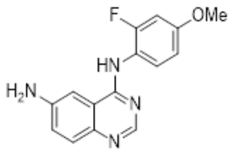


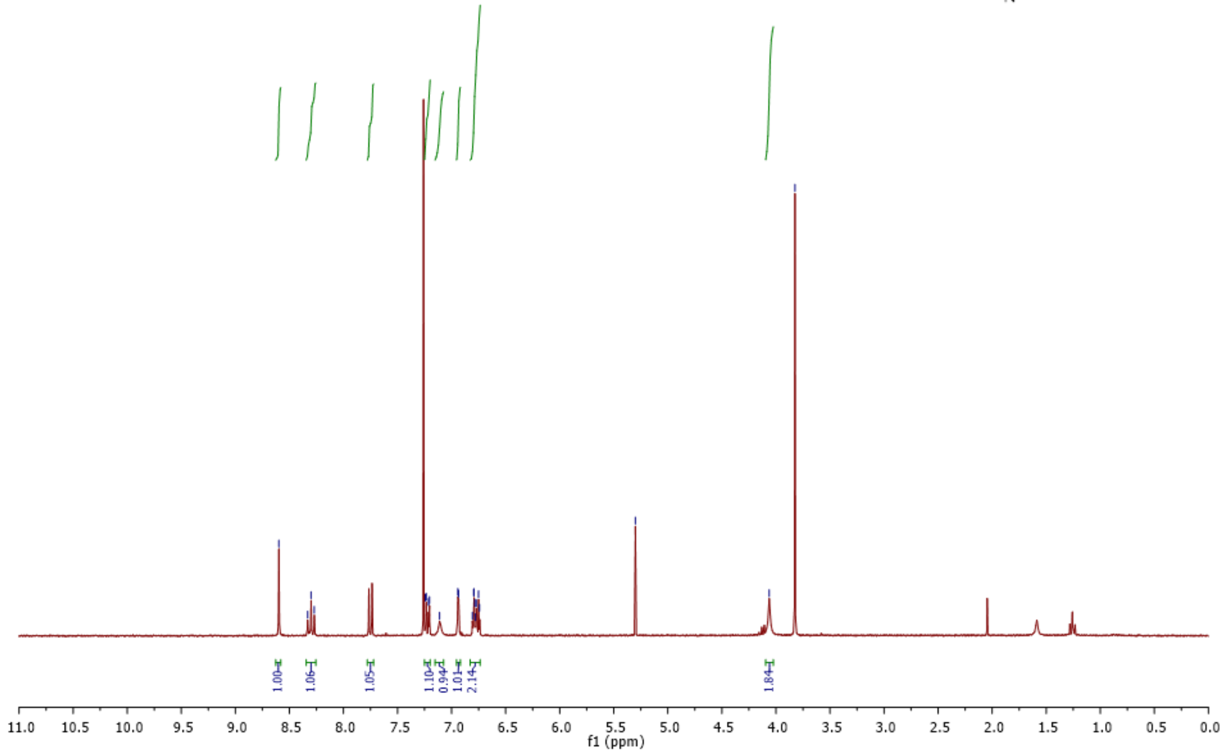


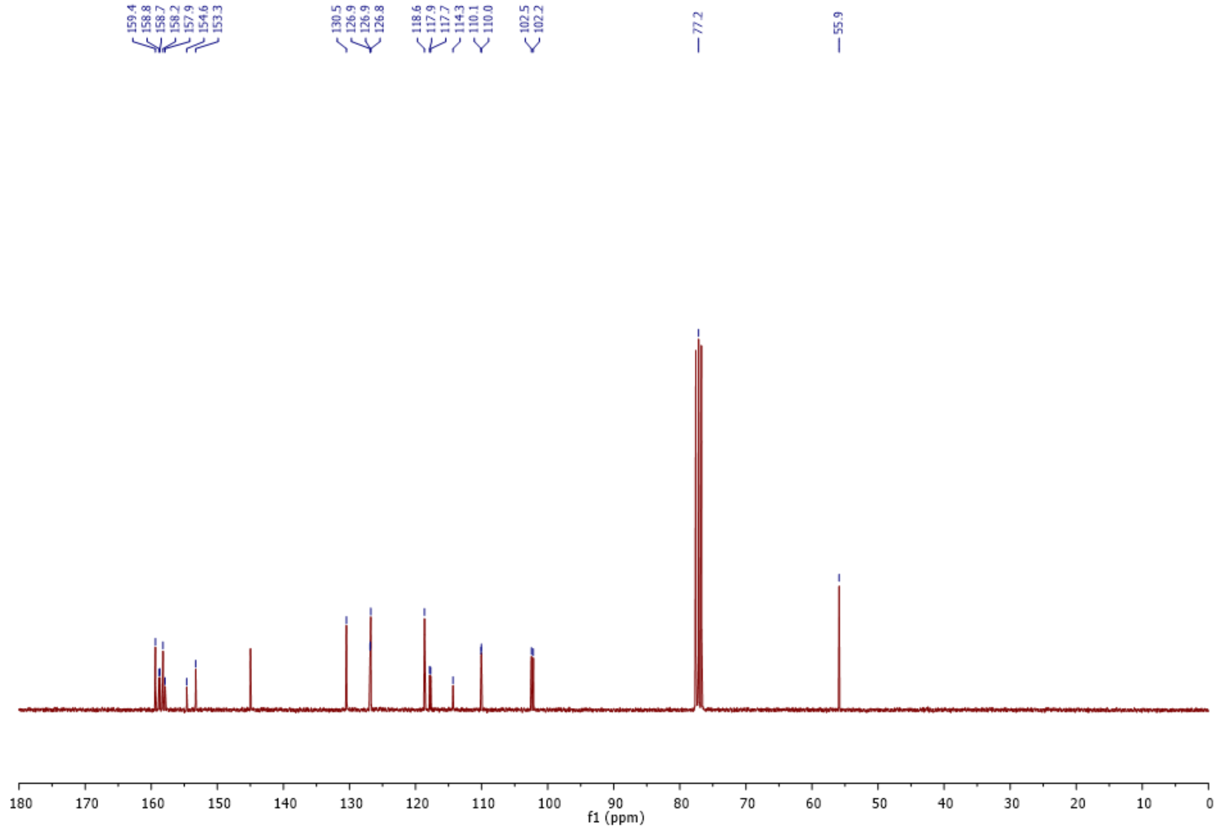

*N*[4]-(2-fluoro-4-methoxyphenyl)quinazoline-4,7-diamine (***11b***).

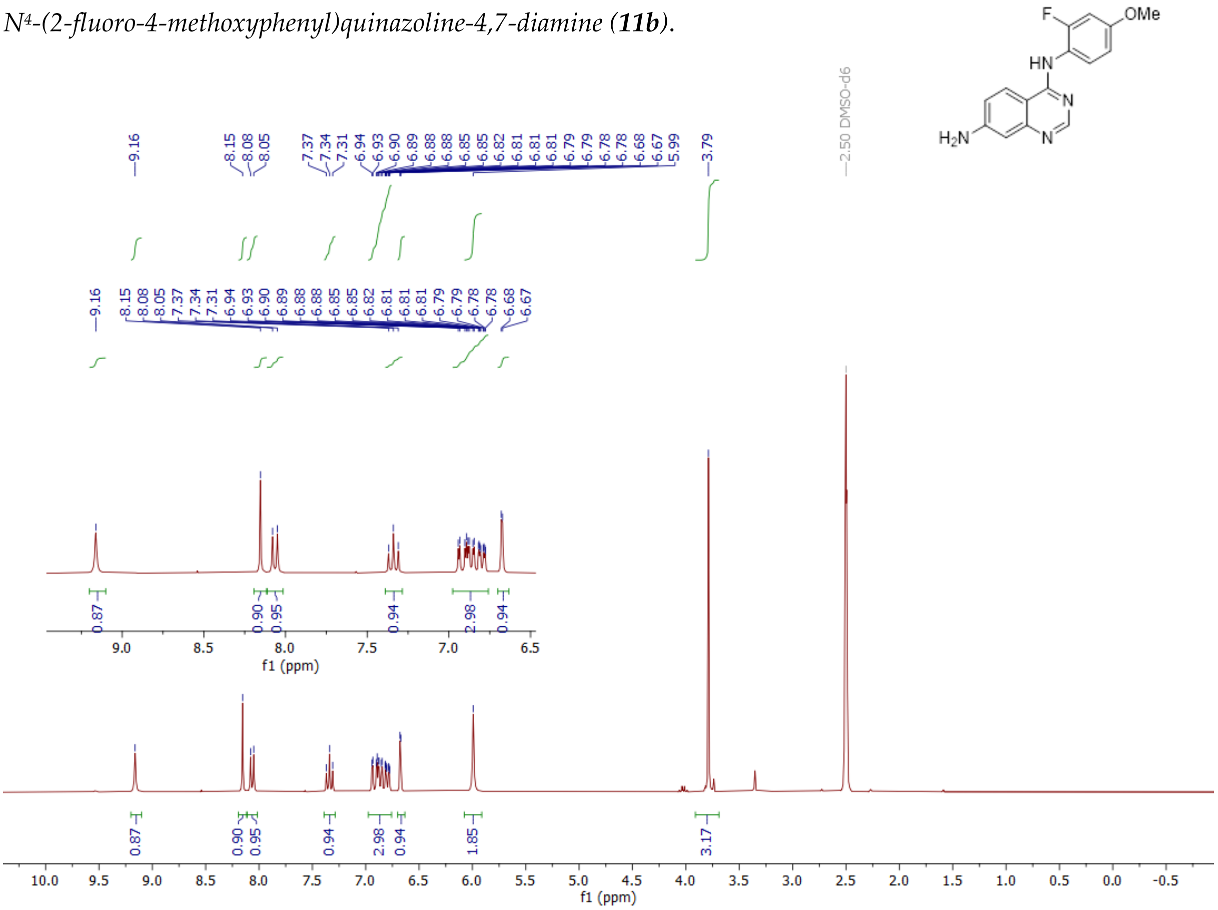


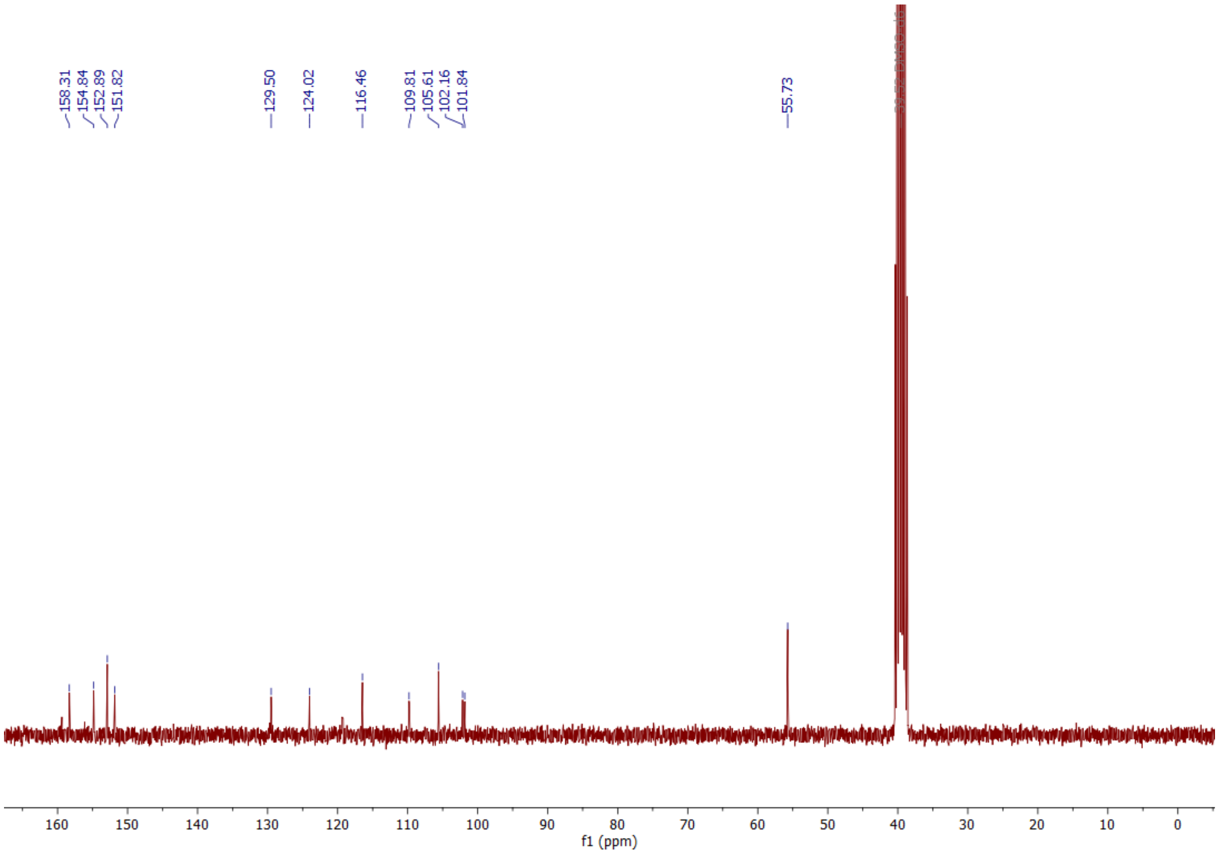

*(4-[(2-Fluoro-4-methoxyphenyl)amino]quinazolin-6-yl)carbamothioyl cyanide* ***(13a).***

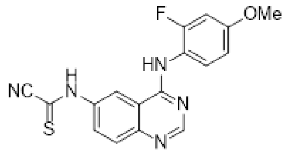

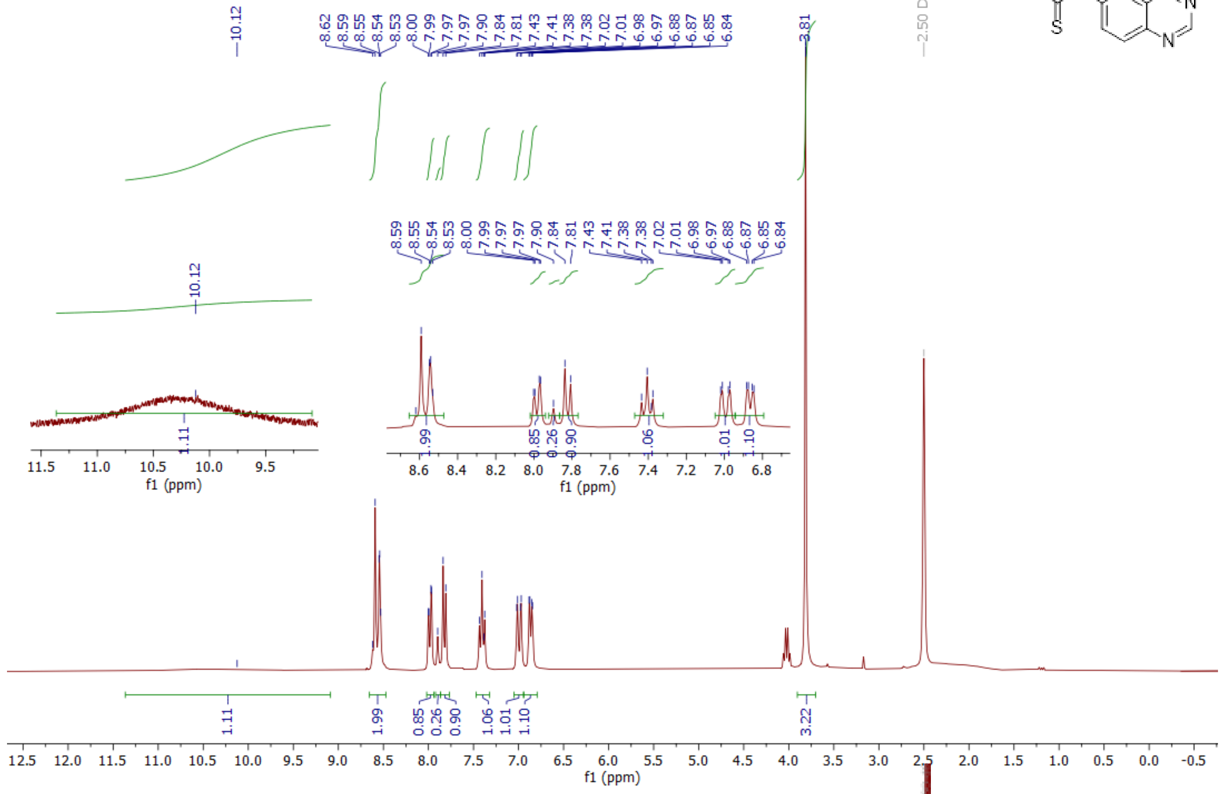

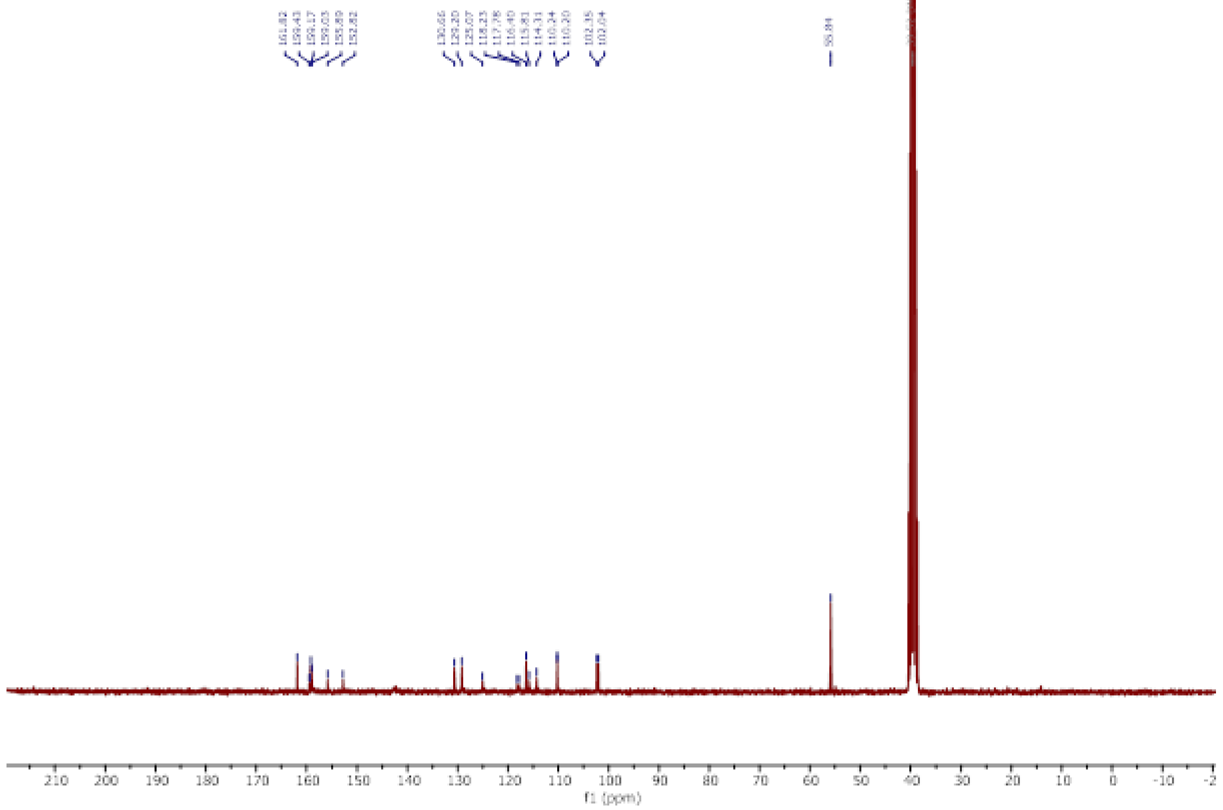

*(4-[(2-Fluoro-4-methoxyphenyl)amino]quinazolin-7-yl)carbamothioyl cyanide* ***(13b).***

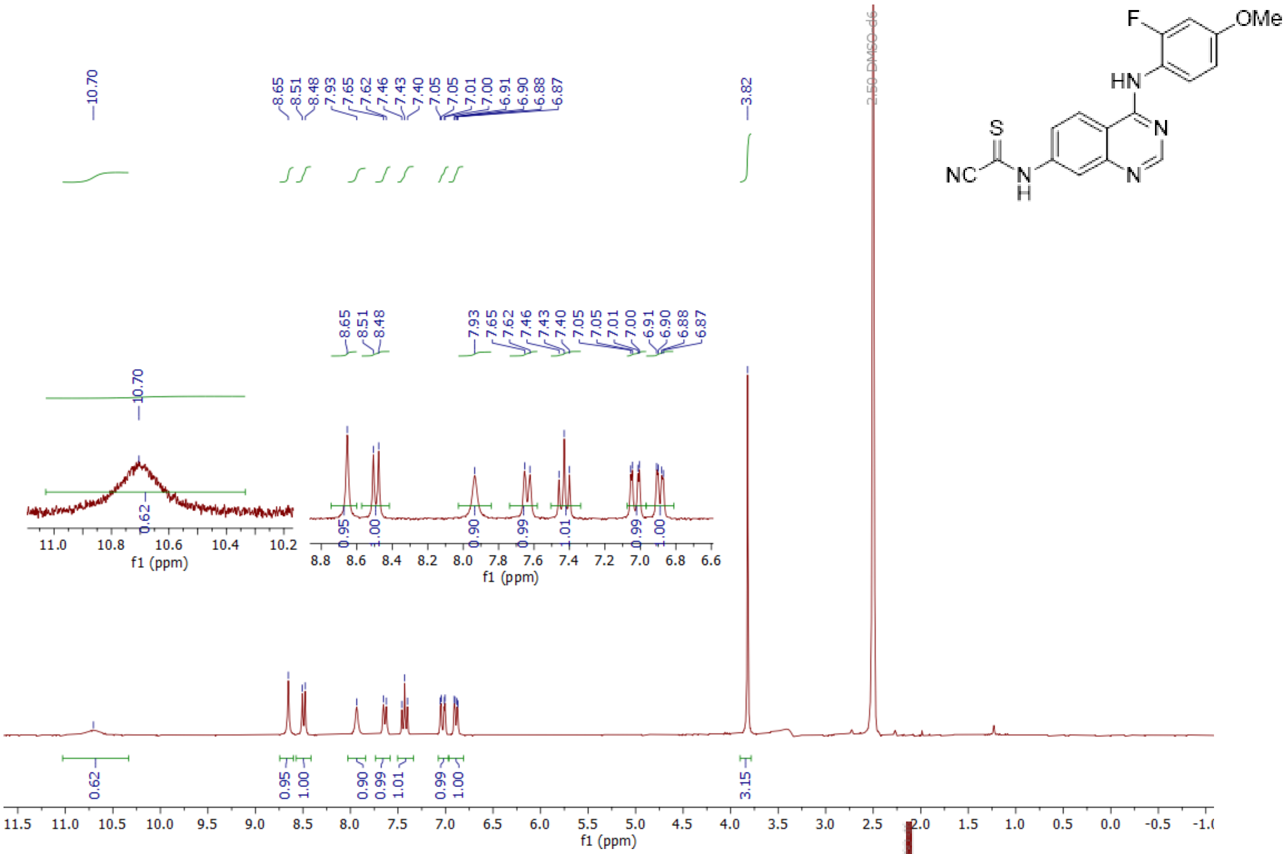


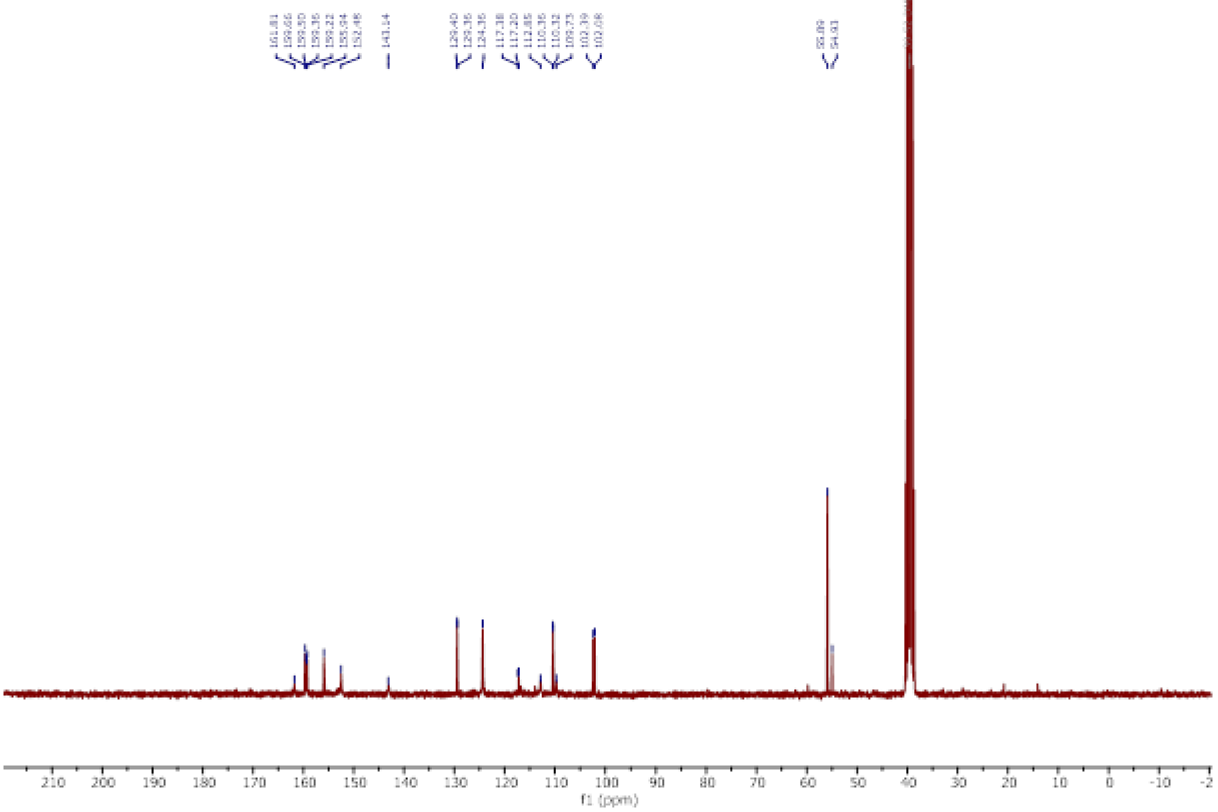

*8-[(2-Fluoro-4-methoxyphenyl)amino]thiazolo[4,5-g]quinazoline-2-carbonitrile (14a).*

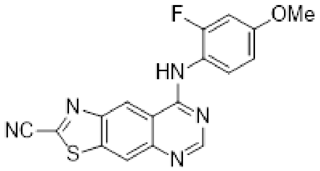


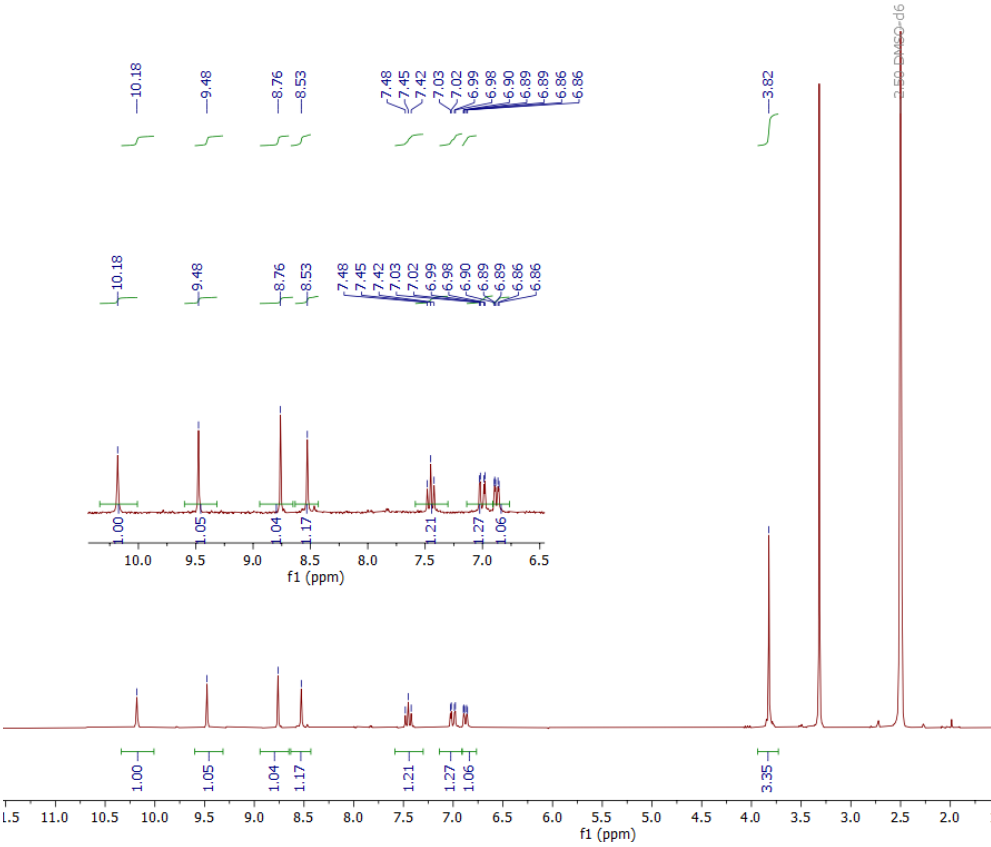


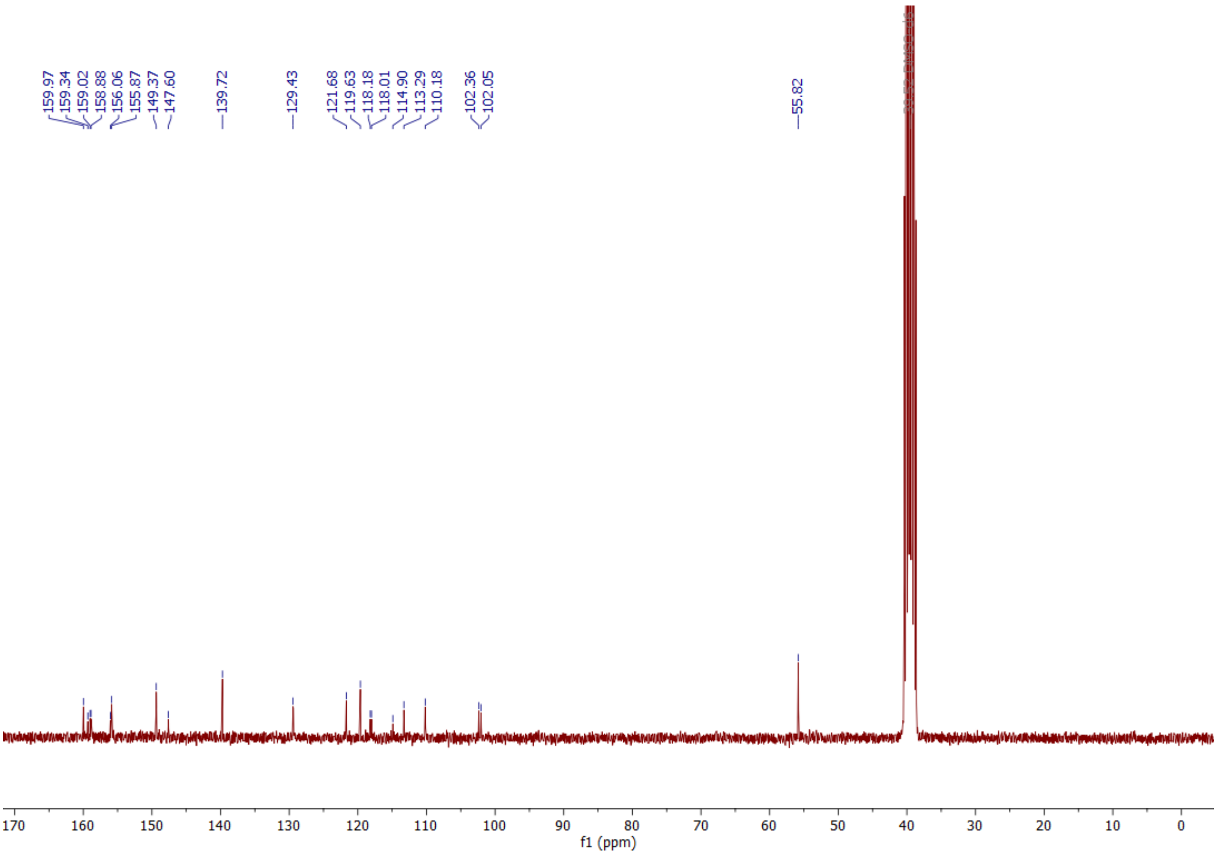

*8-[(2-Fluoro-4-methoxyphenyl)amino]thiazolo[5,4-g]quinazoline-2-carbonitrile* ***(14b).***

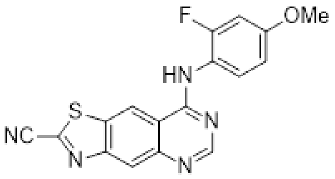


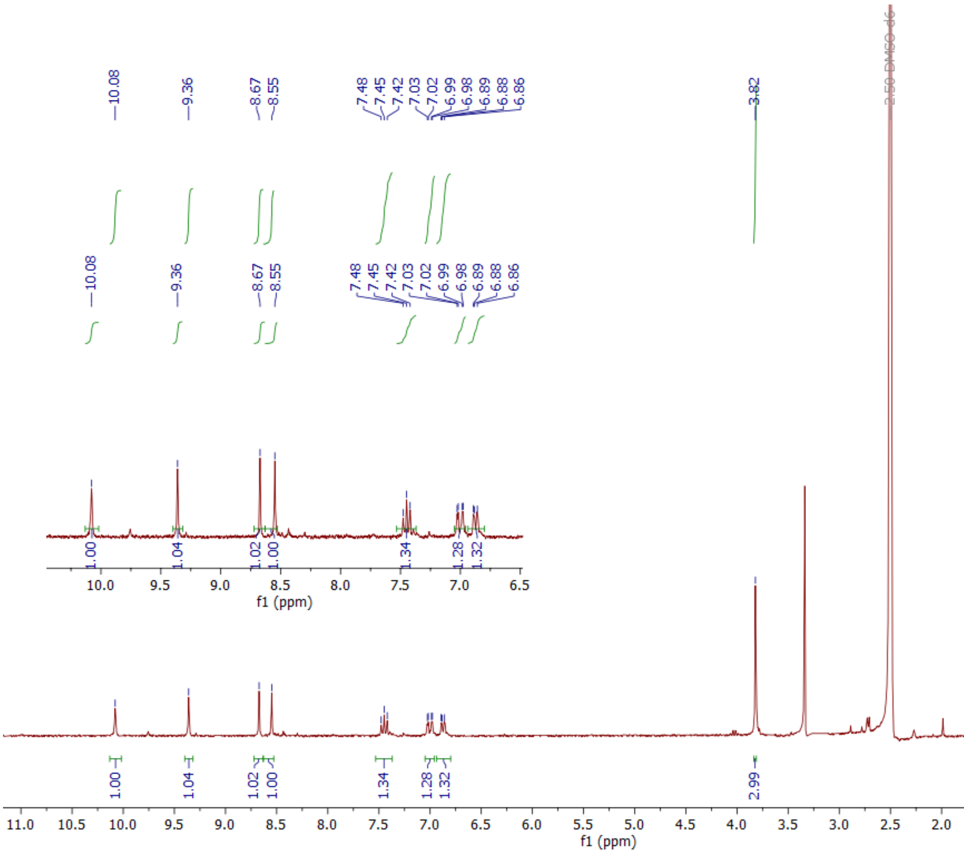


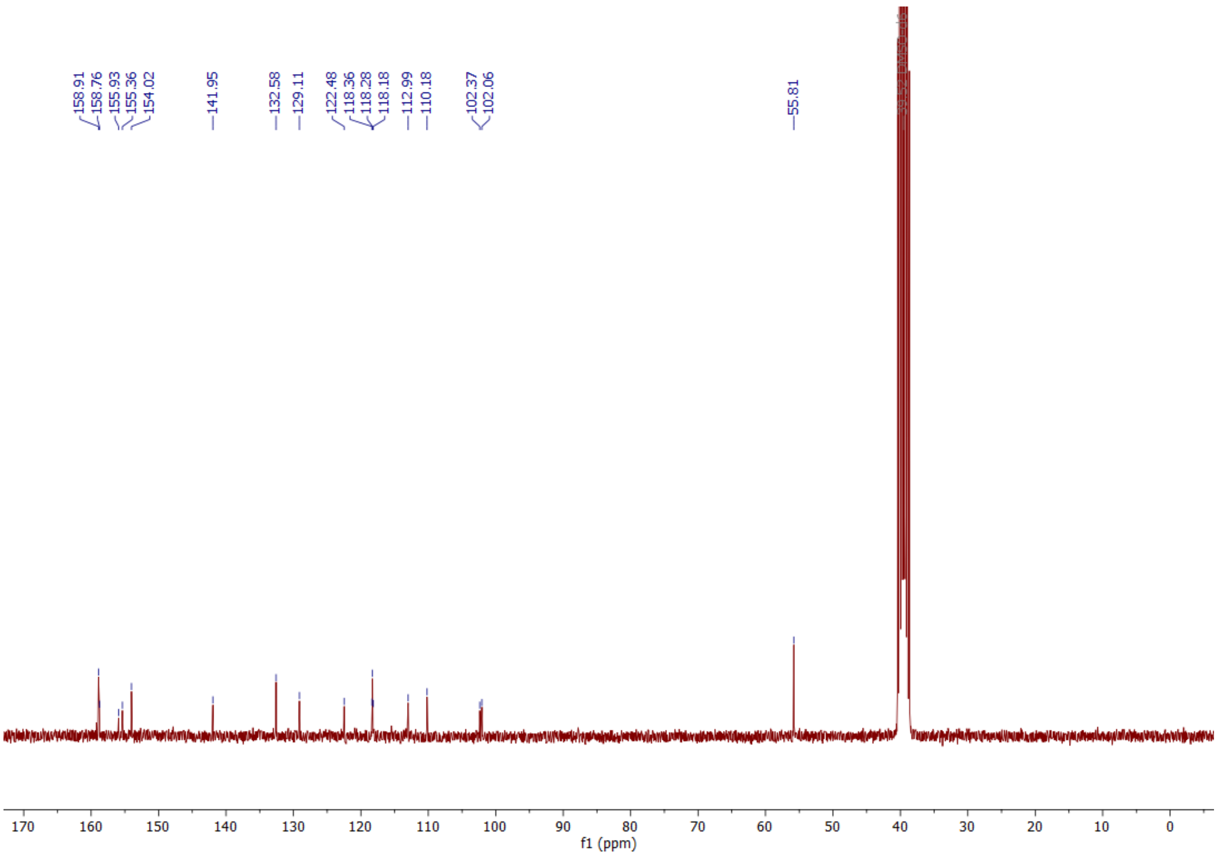

*Methyl 8-[(2-fluoro-4-methoxyphenyl)amino]thiazolo[4,5-g]quinazoline-2-carbimidate* ***(15a).***

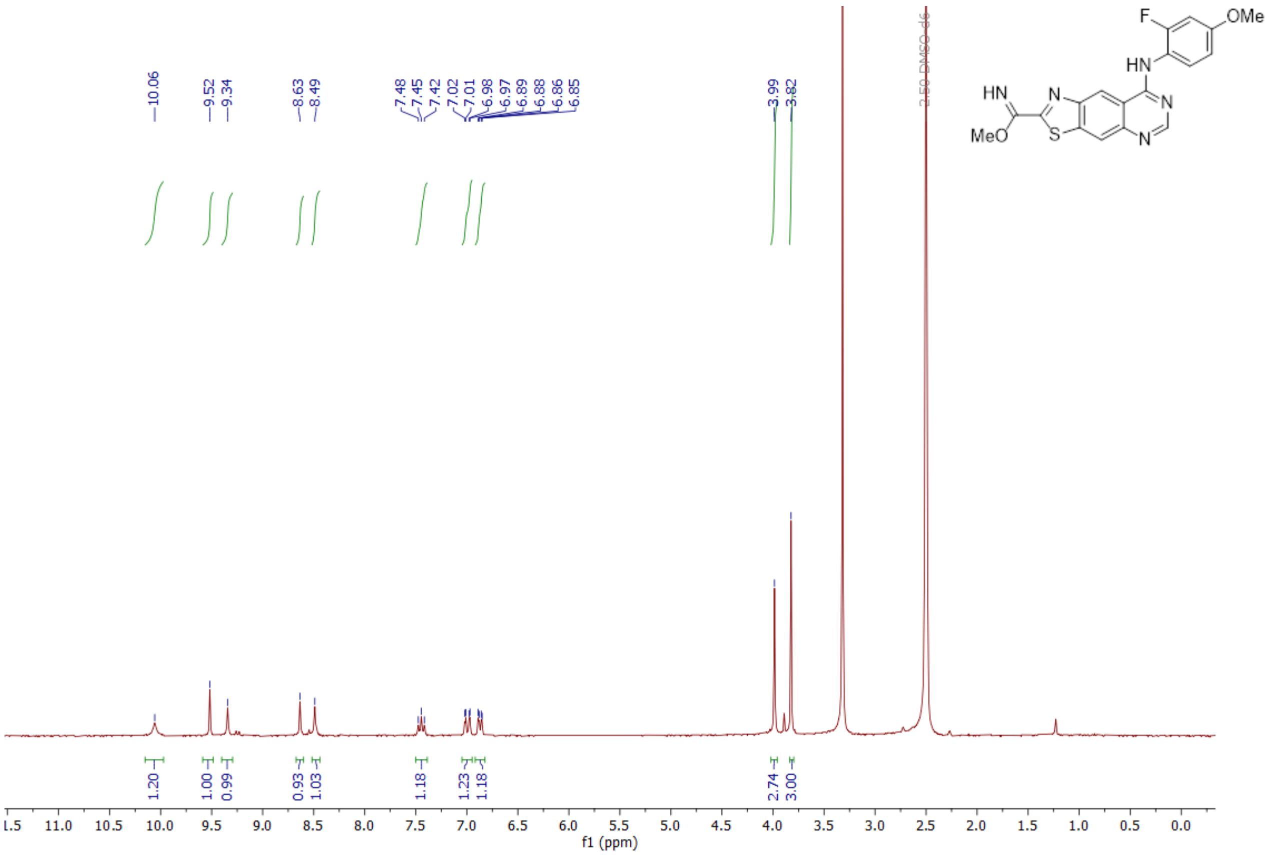

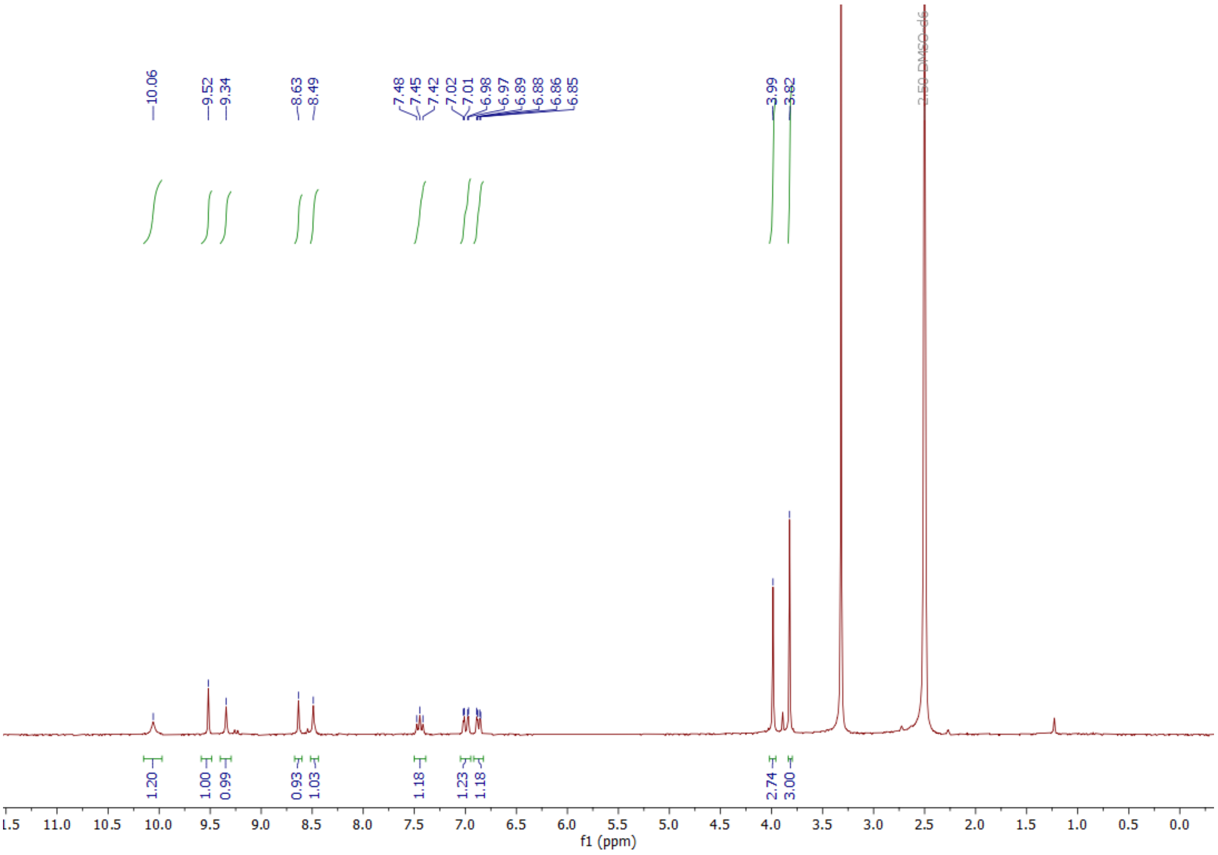

*Methyl 8-[(2-fluoro-4-methoxyphenyl)amino]thiazolo[5,4-g]quinazoline-2-carbimidate* ***(15b)*****.**

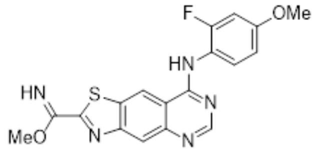

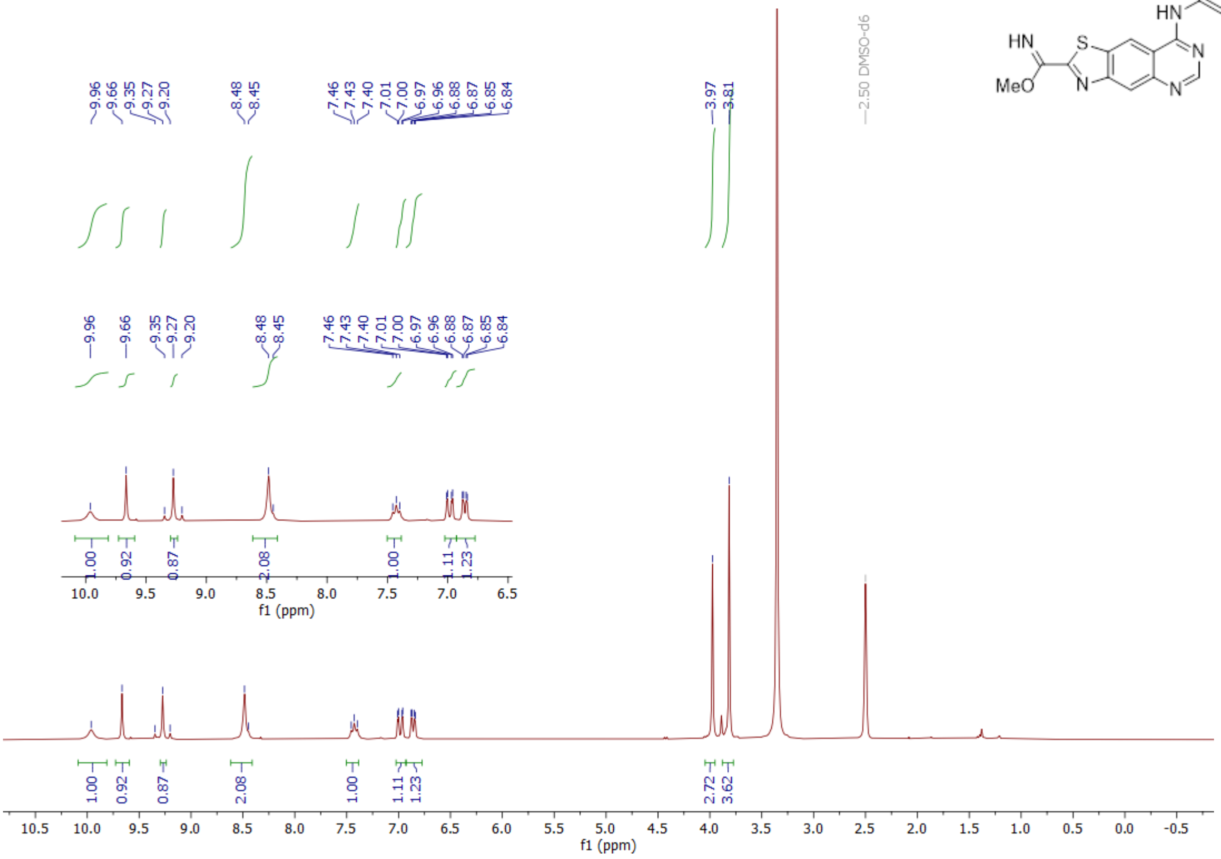

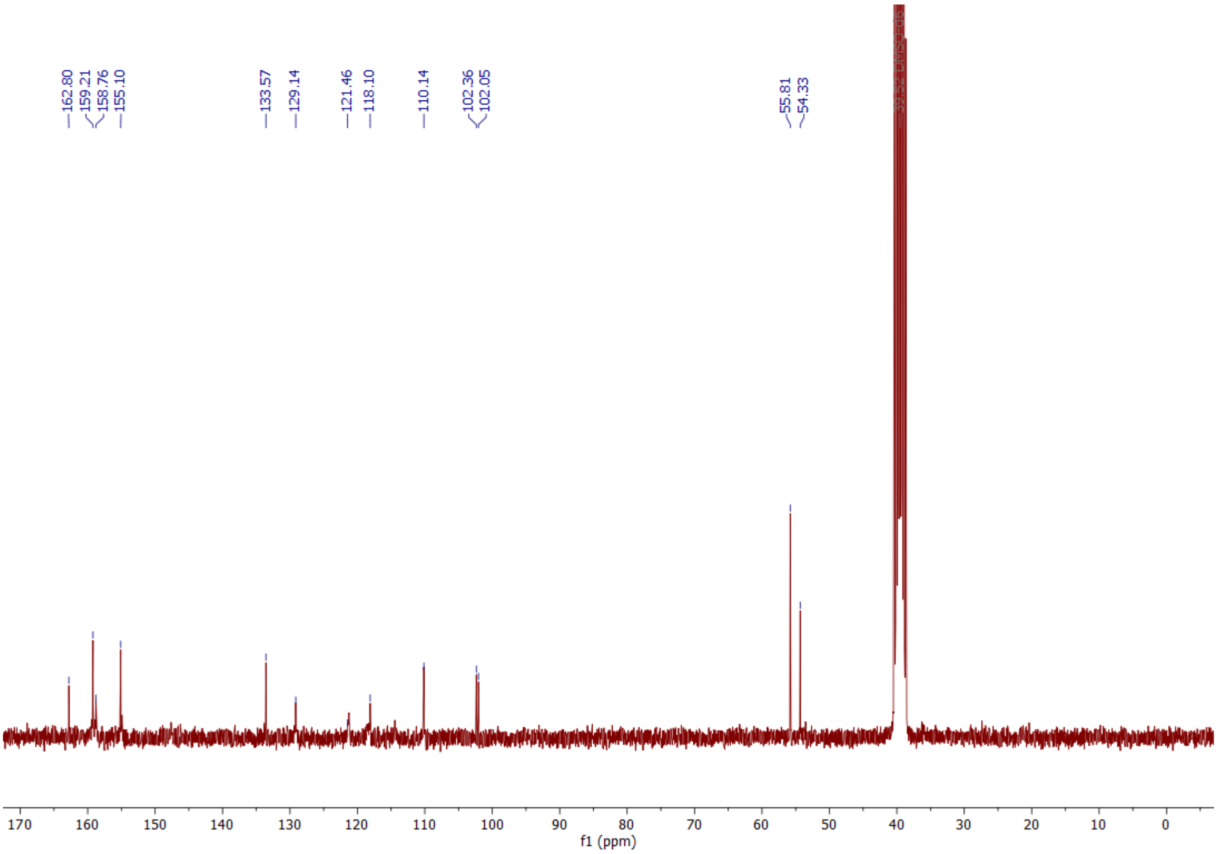

### 3. Kinase inhibition assays

Kinase enzymatic activities were assayed in 384-well plates using the ADP-Glo™ assay kit (Promega, Madison, WI) according to the recommendations of the manufacturer. This luminescent ADP detection assay was described by Zegzouti and co-workers and provides a homogeneous and high-throughput screening method to measure kinase activity by quantifying the amount of ADP produced during a kinase reaction [19]. Briefly, the reactions were carried out in a final volume of 6 µl for 30 min at 30°C in appropriate kinase buffer (10 mM $MgCl_2$, 1 mM EGTA, 1 mM DTT, 25 mM Tris-HCl pH 7.5, 50 µg/mL heparin), with peptide or protein as substrate in the presence of 10 µM ATP. Peptide substrates were obtained from ProteoGenix (Schiltigheim, France) or Sigma for Histone H1, Poly (L-glutamic acid – L-tyrosine) sodium salt, Casein and MBP. After that, 6 µL of ADP-Glo™ Kinase Reagent was added to stop the kinase reaction. After an incubation time of 50 min at room temperature (rt), 12 µL of Kinase Detection Reagent was added for 1 h at rt. The transmitted signal was measured using the Envision (PerkinElmer, Waltham, MA) microplate luminometer and expressed in Relative Light Unit (RLU). Kinase activities are expressed in percentages of remaining kinase activities detected after treatment with 1 or 10 µM of the tested compounds. The maximal kinase activity is measured in the absence of inhibitor but with an equivalent dose of DMSO (solvent of the tested compounds).

**Table.** Human protein kinases tested in the panel: CDK9, HASPIN, PIM1, GSK3b, CK1ε, JAK3, CLK1 et DYRK1A

| **Protein Kinase (Family)** | **Enzyme Description** | **Substrate**[1] *(Working concentration)* |
|---|---|---|
| **CDK9/CyclinT (CMGC)** | Human, recombinant, expressed by baculovirus in Sf9 insect cells | Peptide: YSPTSPSYSPTSPSYSPTSPSKKKK (*83µM*) |
| **CLK1 (CMGC)** | Human, recombinant, expressed by baculovirus in Sf9 insect cells | Peptide: GRSRSRSRSRSR (57.3µM) |
| **DYRK1A (CMGC)** | Human, recombinant, expressed by baculovirus in Sf9 insect cells | Peptide: KKISGRLSPIMTEQ (*10.7µM*) |
| **PIM1 (CAMK)** | Human proto-oncogene, recombinant, expressed in bacteria | Peptide: ARKRRRHPSGPPTA (630 µM) |
| **GSK3⚜ (CMGC)** | Human, recombinant, expressed by baculovirus in Sf9 insect cells | Peptide: YRRAAVPPSPSLSRHSSPHQSpEDEEE [3] (*20µM*) |
| **HASPIN (Other)** | Human, kinase domain, amino acids 470 to 798, recombinant, expressed in bacteria | Histone H3 peptide (1-21): ARTKQTARKSTGGKAPRKQLA (*8µM*) |
| **CK1ε (CK1)** | Human, recombinant, expressed by baculovirus in Sf9 insect cells | Peptide: RRKHAAIGSpAYSITA [2] (*170µM*) |
| **JAK3 (TK)** | Human, recombinant, expressed by baculovirus in Sf9 insect cells | Peptide: GGEEEEYFELVKKKK (94µM) |

[1] Peptide substrates were obtained from ProteoGenix (Schiltigheim, France) or Sigma for Histone H1, Poly (L-glutamic acid – L-tyrosine) sodium salt, Casein and MBP; [2] "Sp" stands for phosphorylated serine.

Controls inhibitors: To validate each kinase assay, the following model inhibitors were used under the same conditions than the tested compounds: Staurosporine from *Streptomyces* sp. (#S5921, Sigma-Aldrich) for CK1ε; Indirubin-3'-oxime (#I0404, Sigma-Aldrich) for CDK9/CyclinT, GSK3◎, DYRK1A and CLK1; CHR-6494 (#SML0648, Sigma-Aldrich) for HASPIN; Tofacitinib (CP-690550, #S2789, Selleckchem) for JAK3; SGI-1776 (#S2198, Selleckchem) for Pim1.

**4. Cytotoxic activity**

4.3.1. Cell culture. Skin normal fibroblastic cells are purchased from Lonza (Basel, Switzerland). Huh7-D12, Caco-2, HCT-116, MDA-MB-231, MDA-MB-468, MCF-7 and PC-3, cancer cell lines were obtained from ATCC (American Type Culture Collection). Cells are grown at 37°C, 5% $CO_2$ in ATCC recommended media: DMEM for Huh7-D12, MDA-MB-231, MDA-MB-468 and fibroblastic cells, EMEM for MCF-7 and Caco-2, McCoy's for HCT-116 and RPMI for PC-3 cells. All culture media are supplemented by 10% of FBS, 1% of penicillin-streptomycin and 2 mM glutamine.

4.3.2. Cytotoxic assay. Chemicals were solubilized in DMSO at a concentration of 10 mM (stock solution) and diluted in culture medium to the desired final concentrations. The dose effect cytotoxic assays ($IC_{50}$ determination) were performed by increasing the concentrations of each chemical (final well concentrations: 0.1 μM, 0.3 μM, 0.9 μM, 3 μM, 9 μM and 25 μM). Cells were plated in 96-well plates (4000 cells/well). Twenty-four hours after seeding, cells were exposed to chemicals. After 48h of treatment, cells were washed in PBS and fixed in cooled 90% ethanol/5% acetic acid for 20 min and the nuclei were stained with Hoechst 33342 (B2261 Sigma). Image acquisition and analysis were performed using a Cellomics ArrayScan VTI/HCS Reader (ThermoScientific). The survival percentages were calculated as the percentage of cell number after compound treatment over cell number after DMSO treatment. The relative IC50 were calculated using the curve fitting XLfit 5.5.0.5 (idbs) integrated in Microsoft Excel as an add-on. The 4 Parameter Logistic Model or Sigmoidal Dose-Response Model describing the sigmoid-shaped response pattern was used (fit = (A+[(B-A)/(1+[(C/x)^D])]) where fit is the response and X is the tested concentration of the compound. The lower asymptote is A (also referred to as the bottom of the curve or lower plateau or the min) and the upper asymptote is B (also referred to as the top of the curve or upper plateau or the max). The slope of the linear part of the curve is described by the factor D. The parameter C is the concentration corresponding to the response midway between A and B. C is called "relative IC50" because it refers to the maximum response obtained by a compound for which a complete dose-response curve has been realized. The results are represented as the mean ± standard error (SE) calculated from at least three independent experiments.
